# RNA-SeqEZPZ: a point-and-click pipeline for comprehensive transcriptomics analysis with interactive visualizations

**DOI:** 10.1093/gigascience/giaf133

**Published:** 2025-11-12

**Authors:** Cenny Taslim, Yuan Zhang, Galen Rask, Genevieve C Kendall, Emily R Theisen

**Affiliations:** Center for Childhood Cancer Research, The Abigail Wexner Research Institute, Nationwide Children’s Hospital, Columbus, OH 43215, USA; High Performance Computing Center, The Abigail Wexner Research Institute, Nationwide Children’s Hospital, Columbus, OH 43215, USA; Center for Childhood Cancer Research, The Abigail Wexner Research Institute, Nationwide Children’s Hospital, Columbus, OH 43215, USA; Center for Childhood Cancer Research, The Abigail Wexner Research Institute, Nationwide Children’s Hospital, Columbus, OH 43215, USA; Department of Pediatrics, The Ohio State University College of Medicine, Columbus, OH 43210, USA; Center for Childhood Cancer Research, The Abigail Wexner Research Institute, Nationwide Children’s Hospital, Columbus, OH 43215, USA; Department of Pediatrics, The Ohio State University College of Medicine, Columbus, OH 43210, USA

**Keywords:** RNA sequencing, RNA-seq analysis pipeline, nextflow, singularity, differential analysis, user friendly interface, pathway analysis, reproducible bioinformatics

## Abstract

**Background:**

RNA sequencing (RNA-seq) analysis has become a routine task in numerous genomic research labs, driven by the reduced cost of bulk RNA sequencing experiments. These studies generate billions of reads that require easy-to-run, comprehensive, and reproducible analysis. However, many labs rely on in-house scripts, which can be challenging for bench scientists to use and hinder standardization and reproducibility. While existing RNA-seq pipelines attempt to address these challenges, they often lack a complete end-to-end user interface.

**Findings:**

To bridge this gap, we developed RNA-SeqEZPZ, an automated pipeline with a user-friendly point-and-click interface, enabling rigorous and reproducible RNA-seq analysis without requiring programming or bioinformatics expertise. For advanced users, the pipeline can also be executed from the command line, allowing customization of steps to suit specific applications. The innovation of this pipeline lies in the combination of 3 key features: (i) all software is packaged within a Singularity container, eliminating installation issues; (ii) it offers a graphical, point-and-click interface from raw FASTQ files through differential expression and pathway analysis; and (iii) it includes a Nextflow implementation, enabling scalability and portability for seamless execution across various platforms, including job submission in the cloud and cluster computing. Additionally, RNA-SeqEZPZ generates a comprehensive statistical report and offers an option for batch adjustment to minimize effects of noise due to technical variation across replicates. Reports can also be reviewed by a bioinformatician to ensure the overall quality of the analysis.

**Conclusions:**

RNA-SeqEZPZ is a robust, accessible, and scalable solution for comprehensive RNA-seq analysis, enabling researchers to focus on biological insights rather than computational challenges.

Key Points

- RNA-SeqEZPZ is a user-friendly pipeline with a point-and-click interface starting from raw FASTQ files for comprehensive RNA sequencing analysis, enabling both novice and experienced users to perform complex analyses with ease.
- RNA-SeqEZPZ enables researchers to analyze and compare differential gene expression across varying experimental conditions, with intuitive visualization tools for exploring and interpreting results.
- RNA-SeqEZPZ provides a containerized image and uses bioinformatics systems managers, ensuring straightforward installation, seamless deployment across environments, and reproducibility of the analyses performed.
- RNA-SeqEZPZ is freely available and can be downloaded from https://github.com/cxtaslim/RNA-SeqEZPZ and https://github.com/yzhang18/RNA-SeqEZPZ-NF (Nextflow version).

## Introduction

Data analysis of RNA sequencing (RNA-seq) consists of a set of successive stages that are repetitive and routinely executed using a wide variety of tools. Typically, analysis starts with quality control of raw sequence reads or FASTQ files, followed by alignment of reads to a reference genome, filtering of low-quality reads, counting reads that align to a specific feature/gene, differential analysis of genes in different conditions, and finally visualization of the results [1]. In-house analysis usually involves a bioinformatician creating step-by-step scripts for specific datasets that will need to be modified for different datasets. With each modification and customization, it is notoriously challenging to keep the analysis fully reproducible, primarily due to differences in scripts, hardware, operating systems, and software versions. Reproducibility is critical for a rigorous analysis to ensure reliable validation of scientific findings and has long been a challenging issue in biomedical research [2]. A recent publication found that most existing Jupyter notebooks (a popular format for documenting and sharing computational workflow) could not be executed automatically and failed to reproduce the results [3] . Reproducibility issues have even led to a retraction of an epidemiological paper [4].

Furthermore, wet lab scientists who conduct the RNA-seq experiments and generate libraries often have limited programming and bioinformatics experience, making it challenging for them to analyze their own data efficiently while ensuring statistical rigor and reproducibility. This creates a strong demand for an easy-to-use, comprehensive pipeline that expedites routine RNA-seq analysis without sacrificing the quality and reproducibility of the results. Here, we describe RNA-SeqEZPZ [5, 6], a point-and-click tool for comprehensive analysis of RNA-seq experiments from FASTQ to result visualization. RNA-SeqEZPZ is primarily designed to empower bench scientists to do their own analyses and explore their results while also providing bioinformaticians with the flexibility for further customization.

Several RNA-seq pipelines exist, with ENCODE [7] and nf-core [8] among the most widely used in the community. In comparison to these pipelines, a notable feature of RNA-SeqEZPZ is its point-and-click interface, starting from FASTQ files up to differential gene analysis and interactive visualization capabilities. ENCODE does not perform differential gene analysis and has no interactive visualization. The nf-core RNA-seq pipeline itself does not include built-in interactive visualization and differential gene analysis. However, it provides output files that can be used as input to a separate visualization and differential analysis pipeline that must be run independently using a command line after the completion of the RNA-seq pipeline. Several shiny [9] apps providing a graphical interface for RNA-seq analysis, such as ROGUE [10], Shiny-Seq [11], and bulkAnalyseR [12], have also been previously published. However, these tools do not support analyzing RNA-seq experiments starting from raw FASTQ files. Furthermore, at the time of writing, Shiny-Seq appears to be no longer accessible, as its official website [13] redirects to a “not found” page on FastGenomics. Access to ROGUE online [14] was repeatedly interrupted by server issues, which may impact its usability for analysis. We found that Partek flow™ and RaNA-seq [15] offer functionalities most similar to RNA-SeqEZPZ. However, both require users to upload FASTQ files to their server, which can be complicated by connection and firewall restrictions or create privacy concerns if analyzing patient data. In addition, neither pipeline provides access to full source code, limiting customization. RASflow [16] supports analysis from FASTQ files but lacks a graphical interface for selecting these files, which may hinder usability for nontechnical users. Regarding comparative analysis, only bulkAnalyseR and RaNA-seq appear to support such feature. However, bulkAnalyseR restricts comparisons to a maximum of 2 groups, whereas RNA-SeqEZPZ supports comparisons across up to 7 groups. In RaNA-seq, comparative analysis is limited to a Venn diagram of significant gene overlap. In contrast, RNA-SeqEZPZ offers an expanded suite of analysis, including Venn diagrams, gene overlap analysis, and pathway comparisons across groups. A comparison of these tools is provided in Supplementary  Table S1.

To the best of our knowledge, RNA-SeqEZPZ is the first open-source tool to offer a point-and-click interface with interactive plots, starting from raw FASTQ reads and providing analytical capabilities from differential gene analysis to pathway analysis. This pipeline can potentially accelerate research progress by simplifying a complex process, enhancing reproducibility within and across labs, and empowering researchers with the tools to interpret their own results. With the extensive reports generated by the pipeline, a bioinformatician can supervise the entire process by reviewing the reports to ensure accuracy and proper execution.

## Methods

**Figure 1:**
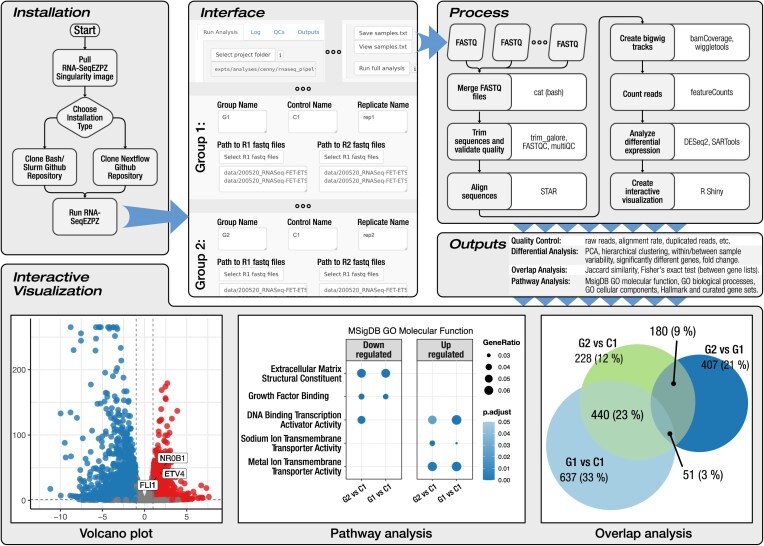
Overview of RNA-SeqEZPZ workflow, interface, and analysis outputs. Installation begins with pulling a Singularity image and cloning a Git repository. After installation, the software can be run with a single command, which launches a web interface, allowing users to select parameters and FASTQ files. Clicking “Run full analysis” triggers multiple processes that generate statistical outputs and provide interactive visual interfaces.

RNA-SeqEZPZ can be started using a single command after downloading a Singularity image and cloning the Git repository (Fig. 1 and Supplementary Fig. S1). It encompasses multiple steps, utilizes various tools, and generates statistical reports, visualization, and diverse output files. The pipeline accepts gzipped paired-end FASTQ files as input and supports analysis for 20 genomes, including human, zebrafish, and mouse. Users can select all the inputs through a point-and-click interface implemented using a shiny [9] app and shinyFiles [17], allowing them to initiate a comprehensive analysis effortlessly.

### Software implementation

RNA-SeqEZPZ is a combination of a shiny [9] app with either bash scripts and SLURM [18] (a cluster resource management system) or Nextflow [19], a workflow management system (Fig. 1). The shiny app at the front end provides an interface for users to run the entire analysis. As SLURM is the most widely used workload manager in high-performance computing (HPC) [20], using it in bash scripts will enable users to easily modify the scripts as needed and leverage their existing familiarity with the system. Nextflow is a modern workflow management system designed to simplify the development and deployment of complex data analysis pipelines. Nextflow enhances the flexibility of this pipeline to run on diverse computation infrastructures with workload managers other than SLURM. The required R packages and all other tools needed for analysis, including Firefox (the browser used for the interface), are enclosed inside a Singularity [21] container, removing any potential difficulties involved in the installation of all the required software. Altogether, this promotes the reproducibility, standardization, and portability of the RNA-SeqEZPZ pipeline. Further, because the shiny app and analysis can be run locally on a cluster, there is no need to transfer gigabytes to terabytes of data to an external server over the Internet.

### Installation and usage

Installation instructions are provided in detail at the GitHub repository. Briefly, a Singularity image, either a Nextflow-based or a bash/SLURM-based version, depending on user preference, containing all the necessary scripts, is cloned from a repository (Fig. 1). To use the pipeline, users need to connect to their HPC cluster and run a 1-line command—“bash run_shiny_analysis.sh”—which will bring up a Firefox browser (provided by the container), where the user will be able to select the sample FASTQ file path, output path, resource requirements, and various settings. Options are also available for running the steps of the pipeline individually (see the manual on the website for details). To assist users running this for the first time, we have provided example datasets that can be downloaded from the GitHub repository, along with an easy-to-follow step-by-step tutorial. A video tutorial is available in Supplementary  File 5.

### Workflow overview

RNA-SeqEZPZ performs multiple steps. The process begins with merging FASTQ files from different sequencing lanes using the cat command in Bash. Raw reads quality control is assessed using default metrics provided by FASTQC [22], and the quality control reports are compiled using MultiQC [23]. For guidance on interpreting FASTQC metrics to identify and remove low-quality files, users may refer to thresholds commonly applied in variant calling analysis [24]. Low-quality bases and adapter sequences are removed using trim_galore [25]. Specifically, bases with a Phred [26] quality score below 20 are trimmed from the 3′ end of the reads. Paired-end reads that become shorter than 20 bp after trimming are discarded. Following quality control and trimming, reads are aligned to the reference genome using the 2-pass approach of STAR [27], which enhances mapping accuracy. Subsequently, gene-level read quantification is carried out using featureCounts [28]. BigWig tracks are generated using bamCoverage [29] and WiggleTools [30] for visualization. Differential expression analysis is performed using DESeq2 [31] with batch adjustment, and statistical reports are generated by SARTools [32]. By default, differentially expressed genes are identified using a false discovery rate (FDR) [33] threshold of 0.05, with no fold-change cutoff applied. These thresholds, along with the minimum difference in normalized count, can be adjusted by users through the graphical interface (see Supplementary Fig. S6). The model incorporates replicates as a covariate to correct for batch effects. Users also have the option to disable batch adjustment directly within the interface (see Supplementary Fig. S2). In the principal component analysis (PCA) plot generated by the pipeline, the effect of batch adjustment is estimated using limma [34].

### Interactive visualization

To provide additional insights into gene expression analysis, RNA-SeqEZPZ includes several interactive visualization tools. Volcano plots are generated using ggplot2 [35] to highlight differentially expressed genes. Area-proportional Euler and Venn diagrams, along with UpSet plots, are generated using Eulerr [36], venn [37], and UpSetR [38] to visualize gene overlaps. The significance of overlap is assessed by testing the independence of 2 variables using Fisher’s exact test [39]. Additionally, the Jaccard index [40], which quantifies the similarity between gene lists, is computed using the GeneOverlap [41] package. For pathway analysis, overrepresentation analysis is conducted using clusterProfiler [42], utilizing gene set annotations from MSigDB via the msigdbr [43] package. These interactive tools provide deeper insights into gene expression functions and biological significance.

### Rationale for tool selection

RNA-SeqEZPZ is designed as an easy-to-use and accessible pipeline for researchers with no prior experience in RNA-seq analysis. To ensure simplicity, a single tool is selected for each step based on best practices and recommendations from the Hitchhikers’ Guide [1]. For advanced users, the code is fully accessible, allowing customization, tool substitution, and modifications as needed.

For read alignment, STAR [27] was chosen due to its high-performance RNA-seq mapping capabilities [44]. The alignment process occurs in 2 stages: first, initial mapping identifies potential novel splice junctions, followed by a refined alignment using both known annotations and the newly detected junctions. This 2-step approach enhances read mapping accuracy and improves sensitivity.

In our pipeline, we focus on quantifying reads at the gene level, as all isoforms of the same gene typically share the same pathway annotations. To achieve this, we selected featureCounts [28], a fast and efficient quantification of mapped RNA-seq based on genome alignment. Additionally, a comparative evaluation of 7 widely used quantification algorithms demonstrated that featureCounts [28] has higher sensitivity in detecting single-isoform genes while delivering comparable performance on real datasets [45, 46].

For differential expression analysis, DESeq2 was selected based on findings by Rapaport et al. [47], who demonstrated its superior specificity and sensitivity, as well as good control of false-positive errors. More recently, the bestDEG [48] study further supports DESeq2’s enhanced sensitivity compared to other tools when applied to human RNA-seq datasets from the MicroArray Quality Control (MAQC) project. In addition, DESeq2 addresses batch effects by incorporating batch variables as covariates within its generalized linear model (GLM) design formula, thereby removing unwanted technical variation.

### Reproducibility

Reproducibility has long been a key issue in bioinformatics analysis [4, 49]. Ensuring the ability to execute an existing workflow and reproduce the same exact results is crucial for advancing scientific research [2]. To achieve this goal, we employed several solutions following best practices [50–52] to ensure RNA-SeqEZPZ is highly reproducible.

#### Software containerization

To prevent dependency mismatches and ensure consistency across computational environments, we encapsulated all software dependencies within a Singularity [21] container. This guarantees that RNA-SeqEZPZ can be used across multiple environments, including local machines, cloud platforms, or an HPC cluster eliminating issues caused by dependency mismatches. Unlike Docker [53], another popular containerization platform that requires root privileges, Singularity [21] operates without the need for elevated permission, making it ideal in shared environments such as HPC clusters. Additionally, using Singularity [21] eliminates manual installation of software on different systems and ensures it yields the same results on different machines.

#### Workflow documentation

Beyond software dependencies, Kim et al. [52] emphasize the importance of comprehensive documentation and readable code for ensuring reproducibility. Documenting analysis steps and software can be challenging, as bioinformatics workflows often consist of a multitude of tools and steps that are chained together to create a complex analysis workflow. Additionally, minimizing manual steps that are required to execute an analysis workflow is crucial, which is why computational pipelines are needed to automate the integration and execution of these tools.

RNA-SeqEZPZ, implemented as a Bash-based pipeline, is designed for readability and ease of use. It automates workflow execution, supports the reanalysis of failed runs, and generates comprehensive documentation on data processing, ensuring transparency, code sharing, and long-term reproducibility. However, tasks such as reanalysis of failed runs and documentation must be implemented manually. To further enhance flexibility, automation, and resource management, RNA-SeqEZPZ leverages Nextflow [19], a powerful bioinformatics workflow manager. Nextflow has been recognized as a key solution for achieving reproducibility by standardizing execution, tracking inputs, and setting consistent runtimes [51]. Beyond ensuring reproducibility, Nextflow enables easy parallelization, job scheduling, reanalysis of failed runs, seamless integration of software containerization, and efficient resource management. Additionally, it automates the generation of an execution report with detailed information, such as input parameters to the pipeline, software versions, and resource usage information, further optimizing workflow efficiency and reproducibility [51].

#### Code sharing and accessibility

To promote transparency and reproducibility, we ensure that all code is publicly accessible via an online repository such as GitHub. This allows other researchers to review, modify, and extend RNA-SeqEZPZ, fostering collaboration and long-term sustainability. The integration of these solutions collectively ensures that RNA-SeqEZPZ maintains a high level of reproducibility.

Below, we describe in more detail the components of the RNA-SeqEZPZ interface, including interactive plots implemented using Shiny [9].

### User-friendly interface and generated outputs

A primary design goal of RNA-SeqEZPZ is to accelerate full analysis of RNA-seq datasets and provide interactive analysis of the results. As such, the pipeline is designed to be run with a 1-line command in the terminal that loads a user-friendly interface implemented as a Shiny [9] app (Fig. 1).

The interface is accessed through a Firefox browser, allowing users to easily zoom in or out, enlarge text, and adjust the window size for better visibility. To run the analysis, users simply select their FASTQ files and provide the necessary information through an intuitive file browser interface (Supplementary Fig. S2). After entering all sample information, clicking “Run full analysis” will automatically execute the full analysis, as described above (Fig. 1).

During the analysis, users can monitor progress through the “Log” tab (Supplementary Fig. S3). Upon completion, the run_rnaseq_full.out log file will display the message “Done running RNA-seq full analysis.” The files in the “Log” tab display the current step being processed by the pipeline. Once the analysis is completed, users will be able to click on the “QC” tab and see all the quality control (QC) metrics compiled by MultiQC [23] (Supplementary Fig. S4). The HTML files generated by MultiQC [23] are interactive as well, which allows for some customization of the plots (Supplementary File 1). The QC report includes metrics for raw reads, alignment rate, number of duplicated reads, percentage of reads aligned to genomic features, and so on. A statistical report of the differential gene analysis can be viewed in the “Outputs” tab (Supplementary Fig. S5). This report is generated using a modified version of SARTools [32]. The report contains a description of raw data, a PCA plot, and hierarchical clustering of samples to explore the variability within and between samples. The statistical report also describes the steps performed in the differential analysis using DESeq2 [31], along with the statistical assumptions and validation of the choices used (Supplementary File 2).

**Figure 2:**
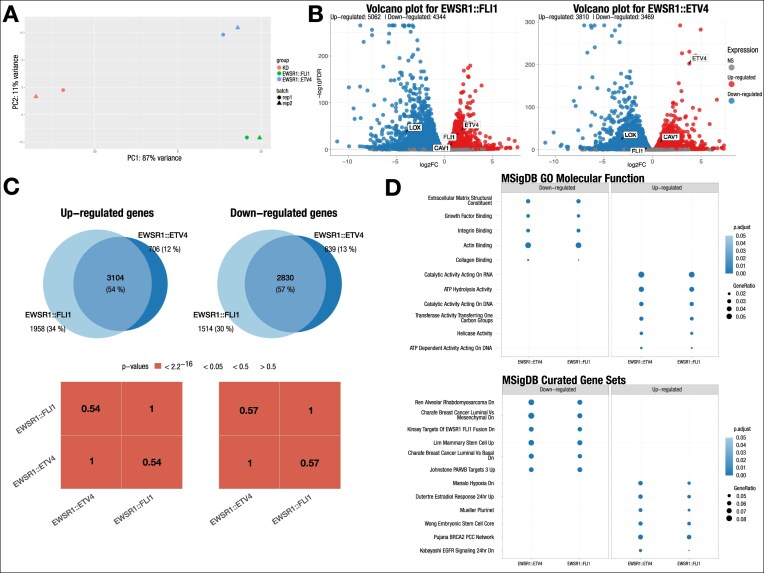
Analysis results of samples rescued with EWSR1::FLI1 and EWSR1::ETV4 constructs. (A) PCA plot showing good separation between the 3 different conditions. (B) Volcano plots for the 2 rescue constructs showing highlighted known targets of EWSR1::FLI1 in addition to FLI1 and ETV4, indicative of the rescue conditions. (C) Overlap analysis reveals a significant overlap between genes regulated by the 2 constructs. Box colors indicate *P* values of overlaps, while the number inside the boxes represents the Jaccard similarity index. (D) Pathway analysis of differentially expressed genes showing enriched Gene Ontology (GO) Molecular function terms and MSigDB Curated Gene Sets. The results indicate that the two constructs regulate similar pathways. Dot color reflects adjusted p-value, and dot size represents gene ratio.

Under the “Plots” tab, users can adjust the cutoffs for significant differential genes, and in the table, they can find the log_2_ fold-change of their gene of interest (Supplementary Fig. S6), create volcano and UpSet plots (Supplementary Fig. S7 and Supplementary Fig. S8), and perform overlap (Supplementary Fig. S9) and pathway analysis (Supplementary Fig. S10). The GeneOverlap [41] package is utilized to compute the Jaccard similarity index [40] and Fisher’s exact test [39] to evaluate the significance of overlap between the gene lists (Fig. 2C). The overlaps between genes in different conditions were visualized using proportional Euler and Venn diagrams, as well as an UpSet plot, created using eulerr [54], Venn [37], and UpSetR [55] packages. Pathway analysis or overrepresentation analysis was conducted using clusterProfiler [42] and msigdbr [43] packages. All other plots were generated using the ggplot2 [56] package.

Additionally, since the files, including intermediate ones generated by the pipeline, can accumulate to terabytes in size, we provide users a simple way to delete projects and files they no longer require (Supplementary Fig. S11). To assist in this process, we provide explanations to help users determine whether to keep or delete these files.

Furthermore, in our Nextflow version, we provide an interface to view the report generated by Nextflow (Supplementary Fig. S12 and Supplementary File 6).

### Public dataset analysis

To demonstrate the utility of our pipeline, we reanalyzed the RNA-seq experiments in the study of novel Ewing sarcoma fusion proteins [57]. RNA-SeqEZPZ was run on 2 biological replicates from a knockdown/rescue experiment in the A673 Ewing sarcoma human cell line, where the endogenous fusion oncogenic transcription factor EWSR1::FLI1 was depleted by short hairpin RNA and then rescued with either EWSR1::FLI1 or EWSR1::ETV4 constructs. These samples were compared to control knockdown cells with no rescue (KD). The FASTQ files can be downloaded from GEO (GSE173185).

As shown in the QC report, for EWSR1::ETV4 rescued sample replicate 1 (iEF_EE4_rep1), there are 48.5 million aligned reads (83.2% alignment rate), and 53.7% of these reads are assigned to a feature (Supplementary File 1). The PCA plot in the statistical report shows that the 6 samples cluster first by replicates and then by rescue condition. This suggests that experimental conditions significantly influence the observed variability and that the samples within each replicate group are highly similar, indicating good reproducibility (Fig. 2). Differential genes were identified with a FDR <0.05. In samples where EWSR1::FLI1 was rescued, FLI1 was correctly upregulated, serving as a surrogate for the EWSR1::FLI1 fusion. In samples where EWSR1::FLI1 was knocked down and then rescued with an EWSR1::ETV4 construct, it shows FLI1 as downregulated and ETV4 as upregulated genes compared to knockdown control (Fig. 2). Well-known targets of EWSR1::FLI1, such as LOX1 and CAV1 [58, 59], are shown as down- and upregulated in both EWSR1::FLI1 and EWSR1::ETV4 rescued samples. There is significant overlap between genes upregulated (3,104 genes, *P* < 0.05) and genes downregulated (2,830 genes, *P* < 0.05) by both EWSR1::FLI1 and EWSR1::ETV4, suggesting that EWSR1::ETV4 regulates genes similar to EWSR1::FLI1. Consistent with overlap analysis that shows a significant overlap between genes, the pathway analysis indicates that genes regulated by EWSR1::ETV4 and EWSR1::FLI1 are involved in many similar functions (Fig. 2 and Supplementary File 3). EWSR1::FLI1 downregulated genes are consistent with those identified in a previous study by Kinsey et al. [60] (Supplementary File 3). The QC report (Supplementary File 1) and statistical report of the differential analysis (Supplementary File 2) are saved as HTML files. All the plots created in RNA-SeqEZPZ can be exported as a pdf file (Supplementary File 3). One of the widely used outputs for downstream analysis is the list of differentially expressed genes. These tables list genes that are defined as significant, along with their Ensembl ID, raw and normalized read count, fold-changes, *P* values adjusted for multiple testing, and other statistics generated by the DESeq2 models (Supplementary File 4). Video tutorial on the analysis of this dataset is included in Supplementary File 5.

#### Portability, scalability, and reproducibility of results

By using RNA-SeqEZPZ instead of in-house scripts, users can more easily run analyses across diverse computational infrastructures with a range of hardware architectures and CPU configurations, while also handling large datasets efficiently. To demonstrate this, we ran RNA-SeqEZPZ on 3 independent datasets across 2 distinct computing environments: (i) the HPC Facility at the Abigail Wexner Research Institute (AWRI) and (ii) the Ohio Supercomputer Center (OSC) [61]. First, we reanalyzed the knockdown/rescue experiments of EWSR1::FLI1 and EWSR1::ETV4, each with 2 replicates and approximately 50 to 60 million paired-end reads (~134 GB total), as previously described (GEO GSE173185). The analysis ran on 2 different HPC clusters with the same cutoffs that produced identical results, identifying 5,062 upregulated and 4,344 downregulated genes by EWSR1::FLI1. Notably, these runs were performed over a year apart, on 30 April 2024 at AWRI and 11 June 2025 at OSC (see Supplementary File 2 for the AWRI run and Supplementary File 7 for the OSC run). Second, we analyzed RNA-seq data from A673 cells treated with either vehicle control (dimethyl sulfoxide [DMSO], *n* = 3) or HCI 2509, an KDM1A inhibitor that has been shown to reverse the transcriptional activity of EWSR1::FLI1 (*n* = 3) [62]. Additionally, we included EWSR1::FLI1 knockdown cells (iEF) cells and RNAi luciferase controls (iLuc), each in quadruplicates (*n* = 4) [63]. The dataset comprises approximately 130 GB of raw FASTQ files, which are available for download from GEO under accession numbers GSE98787 and GSE94503. Both analyses identified a total of 9,797 differentially expressed genes with a FDR ≤0.05, including downregulation of KDM1A in cells treated with HCI 2509 compared to control cells (see Supplementary Fig. S13). The analyses were executed at OSC using 30 CPUs and completed in 1 hour 59 minutes. In comparison, the same analysis at AWRI, run with 20 CPUs, finished in 2 hours 7 minutes (see Supplementary File 8 for the AWRI run and Supplementary File 9 for the OSC run). Finally, we analyzed RNA-seq from 6 hours post fertilization zebrafish embryos injected with human PAX3::FOXO1 compared to control injected embryos, each in quadruplicate [64] (GEO accession: GSE270325), to investigate the *in vivo* activity of the fusion gene. The dataset is approximately 60 GB. Reads were aligned to a custom reference genome that included the PAX3::FOXO1 sequence, enabling quantification of its expression. In both runs, PAX3::FOXO1 was identified as the most highly expressed gene, with a log_2_ fold-change of 14.59 relative to control, followed by tyrp1b, irx4a, and pdia2 (Supplementary Fig. S14).

We have run 3 RNA-seq datasets (from human and zebrafish samples), ranging from 60 GB to 130 GB, across 2 distinct computing environments with varying infrastructures and CPU configurations. All runs produced identical results, emphasizing the scalability and portability of RNA-SeqEZPZ while ensuring the reproducibility of the results.

#### Effects of batch adjustment

**Figure 3:**
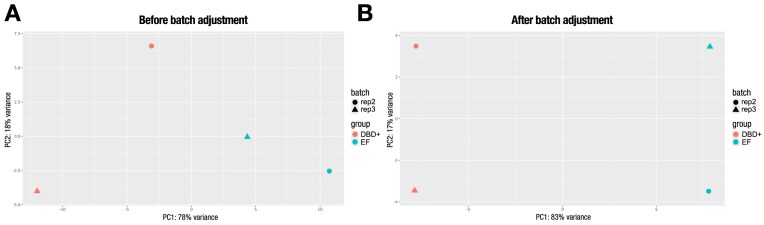
PCA plot demonstrating the impact of batch adjustment. (A) PCA plot before batch adjustment and (B) PCA plot after batch adjustment, showing the improved separation achieved through batch correction.

In order to highlight the benefits of adjusting for batch effects, we reanalyzed RNA-seq experiments from the article, “The DBD-α4 helix of EWSR1::FLI1 is required for GGAA microsatellite binding that underlies genome regulation in Ewing sarcoma” [65]. The FASTQ files were obtained from GEO (GSE268944). RNA-SeqEZPZ was used to analyze 2 biological replicates of knockdown/rescue experiments in the TTC-466, an Ewing sarcoma cell line. Following knockdown of EWSR1::FLI1, rescue was performed using either a mutant construct (DBD+) or the full-length EWSR1::FLI1 construct (EF). Figure 3A presents the PCA plot before batch adjustment. Based on the plot, it is difficult to definitively determine whether the biological replicates cluster together. However, after adjusting for batch effect, the DBD+ replicates cluster together, separating from EWSR1::FLI1 samples along principal component 1 (PC1), which accounts for 83% of the variance (Fig. 3B). Furthermore, batch adjustment increased the variance explained by PC1 from 78% to 83%, further clarifying sample separation.

To further demonstrate the benefit of batch correction on a larger dataset, we reanalyzed RNA-seq data from A673 Ewing sarcoma cells reported in “Seclidemstat blocks the transcriptional function of multiple FET-fusion oncoproteins” [66]. Cells were treated with vehicle (DMSO) or seclidemstat at IC50 or IC90, and RNA-seq was performed in biological triplicate. These samples are available in the GEO (GSE306637). Seclidemstat is currently in clinical trials for FET-rearranged sarcomas (NCT03600649). Prior to batch correction, IC50 and IC90 samples were intermixed. Following batch correction, distinct clusters emerged, yielding clear dose-specific clusters and increasing the variance explained by PC1 by 21% (Supplementary Fig. S20).

RNA-SeqEZPZ offers adjustment to correct for technical differences introduced by processing replicates in batches. By default, the replicate name is treated as a batch variable and added to the GLM as a covariate to be adjusted by DESeq2. Including replicates in the model allows DESeq2 to account for unwanted variation between replicates, effectively adjusting the read counts for each gene or feature. This adjustment helps reduce noise due to technical variability and increases the sensitivity for detecting differentially expressed genes between biological conditions [67]. When the data exhibit significant batch effects and the samples cluster primarily by batch rather than by biological condition of interest in the PCA plot (e.g., different instruments, sequencing runs, technical variations between replicates), it is recommended to perform batch adjustment. In general, when you have a balanced design where the number of replicates is equal across conditions, adjusting for replicate variation can enhance both the sensitivity and precision of the estimate. However, in the case of the A673 cell line, which includes DMSO control (*n* = 3), HCI 2509 treatment (*n* = 3), iLuc control (*n* = 4), and EWSR1::FLI1 knockdown (iEF, *n* = 4), the unadjusted PCA plot already shows clear clustering by condition. Notably, the 2 control groups (DMSO and iLuc) cluster together as expected (Supplementary Fig. S15). After applying replicate-based adjustment, replicate 5 of the iLuc group shifts closer to the DMSO cluster, and replicate 5 of the iEF group becomes more distant from the rest of the iEF replicates. Therefore, for this dataset, it may be best to do the analysis without adjusting for replicates, due to the lack of representation of replicate 5 across all conditions. Users can easily turn off this adjustment by unchecking the “Replicates batch adjustment” option in the user interface (Supplementary Fig. S2). Batch correction for other variables or experimental factors can be carried out by modifying the scripts provided using either a multifactor design in DESeq2 [31] or in combination with ComBat-seq [67].

### Side-by-side comparison with RaNA-seq

RNA-SeqEZPZ was created to enable bench scientists to run their own analysis from beginning processing of the raw FASTQ files to the differential gene analysis. Although other pipelines exist (see Supplementary Table 1), RaNA-seq [15] will be the most comparable to RNA-SeqEZPZ in terms of user-friendliness. RaNA-seq does not require preprocessing of the FASTQ files, provides a user interface for FASTQ file selection, and requires no intensive installation, while also performing differential gene analysis. Its main limitation, however, is the need to upload FASTQ files to a remote server, which can be challenging due to institutional firewalls or other security constraints.

To enable a side-by-side comparison between RNA-SeqEZPZ and RaNA-seq, we analyzed RNA-seq data from nuclear factor (erythroid-derived 2) knockout (Nrf2 KO) mice, which develop lung tumors earlier than wild-type (WT) mice (GEO GSE99338) [68].

#### QC reports

In RaNA-seq, the QC report contains boxplots of the normalized expression values , a bar plot of the estimated number of expressed genes, a heatmap of expression similarity, and a PCA plot, which are similar to the outputs report generated by RNA-SeqEZPZ (see Supplementary File 10 for RaNA-seq and Supplementary File 11, a similar report generated by RNA-SeqEZPZ). One notable difference between our pipeline and theirs is the use of batch adjustment. As a result of this adjustment, PC1 in our analysis explained 75% of the variance, compared to 46.4% in their analysis. This suggests that accounting for variation in the biological replicates allowed PC1 to capture a higher proportion of the variance in the data. Furthermore, the PCA plot shows that samples in our analysis cluster clearly by experimental condition (Supplementary Fig. S16). RNA-SeqEZPZ also provides alignment rate, percentage of duplicates, percent reads that are assigned to a feature, and other raw reads statistics that were not included in RaNA-seq (Supplementary File 12).

#### Differential gene analysis

RaNA-seq identified 375 significant genes while RNA-SeqEZPZ identified 336 significant genes, using an FDR threshold of 0.05. Both pipelines identified Nfe2l2 as the most downregulated gene, along with a set of immune response genes (Cxcl1, Csf1, Ccl9, Cxcl12) that are known to promote tumorigenesis. This upregulation was observed in Nrf2 KO mice, consistent with the previous finding [68] (Supplementary Fig. S17). RNA-SeqEZPZ provides an interface to change the fold-change, FDR, and mean normalized count difference cutoffs, while RaNA-seq only provides FDR cutoff change.

#### Pathway analysis

Both RNA-SeqEZPZ and RaNA-seq show enrichment of immune response. RNA-SeqEZPZ specifically indicates an upregulation of cytokine and chemokine activity. Most importantly, RNA-SeqEZPZ’s curated gene set analysis revealed that genes downregulated in Nrf2 KO compared to WT mice are significantly enriched in the NRF2 pathway [69], highlighting the potential relevance of these findings to human biology (Supplementary Fig. S18).

#### Comparative analysis

In RaNA-seq, 2 analyses can be compared after they are individually analyzed. In contrast, in RNA-SeqEZPZ, all samples need to run together. This way, all samples will be normalized together to correct for library size, and dispersion will incorporate the within-group variability across all groups, which will make their expression values comparable and minimize batch effects such as GC content, length, or other technical biases. To enable comparative analysis, we reanalyzed the Nrf2 KO dataset using an FDR threshold of 0.1 (Nrf2_KO2) and compared the results to the previous Nrf2 KO versus WT analysis with an FDR threshold of 0.05. A Venn diagram illustrating the overlap of significant genes between the 2 analyses is provided as the sole comparison output by RaNA-seq (Supplementary Fig. S19A). In RNA-SeqEZPZ, we added a second Nrf2 KO sample and performed a similar comparative analysis, evaluating the overlap of significant genes identified at FDR ≤0.05 and ≤0.1. Area-proportional Euler diagrams illustrate the overlap of significant genes, stratified by direction of regulation. Additionally, a gene overlap analysis and a Jaccard similarity index were conducted to quantify the degree of similarity (Supplementary Fig. S19B). The analysis also includes pathway enrichment results for both comparison groups (Supplementary Fig. S19C).

Some of the figures for the analyses of public datasets were generated using RNA-SeqEZPZ and modified using graphic editing software. ChatGPT [70] was utilized to assist in checking grammar and improving the clarity of the manuscript draft.

## Discussion

In summary, RNA-SeqEZPZ provides an easy point-and-click comprehensive analysis of RNA-seq data, which enables biologists to analyze and explore the nuances of their own experiments. The implementation of RNA-SeqEZPZ ensures reproducible analysis and is broadly flexible for running in various computational infrastructures. RNA-SeqEZPZ also provides an entry point analysis for more advanced users, who can download the results and do additional downstream analysis or modify the pipeline to include more features. Thus, RNA-SeqEZPZ represents a valuable easy-to-use tool for the scientific community, enabling the analysis, interpretation, and discovery of insights about gene function and regulation through RNA-seq experiments. By integrating the Singularity container with workflow management systems and offering an end-to-end user interface, the codebase provides a flexible and extensible framework. It can be easily expanded to support additional interactive visualizations and more advanced analyses, such as single cell RNA-seq, spatial transcriptomics, and multiomics integration. For example, to enhance the precision and specificity of differential gene detection in future iterations, we may incorporate a consensus-based approach as implemented in bestDEG [48].

## Availability of Source Code and Requirements

Project name: RNA-SeqEZPZ

Project homepage: https://github.com/cxtaslim/RNA-SeqEZPZ

Nextflow pipeline repository: https://github.com/yzhang18/RNA-SeqEZPZ-NF

License: CC BY-NC-ND 4.0 (RNA-SeqEZPZ), CC BY-NC-ND 4.0 (RNA-SeqEZPZ-NF)

Operating system: Platform independent

Programming language: R, Nextflow, Shell

Package management: Singularity container

Hardware requirements: depends on samples analyzed

WorkflowHub: https://doi.org/10.48546/WORKFLOWHUB.WORKFLOW.1813.2 (RNA-SeqEZPZ), https://doi.org/10.48546/WORKFLOWHUB.WORKFLOW.1814.2 (RNA-SeqEZPZ-NF)

## Supplementary Material

giaf133_Supplemental_Files

giaf133_Authors_Response_To_Reviewer_Comments_Original_Submission

giaf133_Authors_Response_To_Reviewer_Comments_Revision_1

giaf133_GIGA-D-25-00067_Original_Submission

giaf133_GIGA-D-25-00067_Revision_1

giaf133_GIGA-D-25-00067_Revision_2

giaf133_Reviewer_1_Report_Original_Submission Unitsa Sangket, Ph.D. -- 5/2/2025

giaf133_Reviewer_2_Report_Original_Submission Yang Yang -- 5/6/2025

giaf133_Reviewer_2_Report_Revision_1 Yang Yang -- 8/21/2025

## Data Availability

New data for A673 cells treated with DMSO or seclidemstat at IC50 and IC90 have been uploaded to the NCBI Gene Expression Omnibus (GEO) repository under accession number GSE306637. Additional datasets used in this study are available in GEO as follows: GSE173185 (knockdown/rescue of EWSR1::FLI1 and EWSR1::ETV4), GSE98787 and GSE94503 (knockdown/rescue of EWSR1::FLI1, iLuc control, and cells treated with DMSO or HCI 2509), GSE268944 (TTC-466 samples), and GSE270325 (zebrafish RNA-seq). Supplemental Files 1, 2, 5–9, and 11–12, along with the archived software and input files, are stored in the GigaDB [72].

## Additional Files

**Supplementary Table S1**. A comparison of existing RNA-seq pipelines with RNA-SeqEZPZ.

**Supplementary Fig. S1**. RNA-SeqEZPZ workflow showing output files generated. Icons were adapted from images created by Regan Hayward under the MIT license [71].

**Supplementary Fig. S2**. A screenshot of the run analysis interface where users will be able to click-and-select their FASTQ files, reference genome, and other inputs. The “i” icon provides more information when hovered over in the interface.

**Supplementary Fig. S3**. A snapshot of the log file providing information on the current progress of the RNA-seq analysis.

**Supplementary Fig. S4**. A screenshot of the quality control report in interactive HTML format that can be viewed by users by clicking the “QCs” tab.

**Supplementary Fig. S5**. A screenshot of the interface in RNA-SeqEZPZ to view and interact with the statistical report generated automatically. The left navigation bar makes it easy to move to different section of the HTML file.

**Supplementary Fig. S6**. A screenshot of the table interface showing the sorted log_2_ fold-change, false discovery rate (FDR), and read counts difference between treatment and control samples. Users can type in gene names to find their expressions.

**Supplementary Fig. S7**. A screenshot of the interface where users can highlight specific genes in the volcano plot and change their significance cutoffs.

**Supplementary Fig. S8**. A screenshot of the interface to create the UpSet plot.

**Supplementary Fig. S9**. A screenshot of the interface to perform overlap analysis, which includes generation of an area-proportional Euler diagram, a Venn diagram up to a 7-group overlap and a heatmap showing the *P* values of the overlap, and the Jaccard similarity index.

**Supplementary Fig. S10**. A screenshot of the interface to perform pathway analysis with gene sets from the MsigDB database. Enrichments are calculated for molecular function, biological processes, cellular components, gene ontologies (GOs), and curated and Hallmark gene sets (not shown).

**Supplementary Fig. S11**. The cleanup interface to assist users with removing big files, such as aligned and merged FASTQ files.

**Supplementary Fig. S12**. The interface to view the report generated by Nextflow.

**Supplementary Fig. S13**. Volcano plot of cells treated with HCI 2509 run at (A) AWRI and (B) OSC.

**Supplementary Fig. S14**. Expression of PAX3::FOXO1 in runs at (A) AWRI and (B) OSC.

**Supplementary Fig. S15**. PCA plot of cells treated with HCI 2509, DMSO control, iLuc control, and EWSR1::FLI1 KD (iEF) cells. (A) PCA plot prior to batch adjustment, showing clear separation between conditions. (B) Batch adjustment leads to mixing of replicates across conditions, arguing against using batch correction.

**Supplementary Fig. S16**. PCA plots of Nrf2 KO samples generated using (A) RaNA-seq and (B) RNA-SeqEZPZ. The RNA-SeqEZPZ plot shows a higher proportion of variance explained by PC1, indicating improved separation after batch correction.

**Supplementary Fig. S17**. Volcano plots of Nrf2 KO samples generated using (A) RaNA-seq, with red dots (from left to right) highlighting Nfe2l2, Clcx1, Cxcl12, and Csf1, and (B) the corresponding volcano plot produced by RNA-SeqEZPZ.

**Supplementary Fig. S18**. Pathway enrichment analysis of Nrf2 KO samples generated using (A) RaNA-seq and (B) RNA-SeqEZPZ. Both analyses show similar enrichment of cytokine activity. Additionally, RNA-SeqEZPZ reveals enrichment of the human NRF2 pathway among downregulated genes.

**Supplementary Fig. S19**. Comparative analysis results. (A) Overlap of significant genes identified using RaNA-seq, (B) overlap analysis of significant genes from RNA-SeqEZPZ, and (C) GO molecular function enrichment using RNA-SeqEZPZ, highlighting similar functional categories between the 2 significant gene sets.

**Supplementary Fig. S20**. PCA plots illustrating the impact of batch correction on a larger dataset: (A) before adjustment and (B) after adjustment, with improved separation between conditions.

**Supplementary File 1**. QC report for the knockdown/rescue of EWSR1::FLI1 (iEF_EF) and EWSR1::ETV4 (iEF_EE4) in the A673 cell line.

**Supplementary File 2**. Statistical report for the knockdown/rescue of EWSR1::FLI1 (iEF_EF) and EWSR1::ETV4 (iEF_EE4) in the A673 cell line. PCA plot is shown in Fig. 2A.

**Supplementary File 3**. Plots generated from the interface after the analysis of EWSR1::FLI1 and EWSR1::ETV4 knockdown/rescue in the A673 cell line. Selected plots are shown in Fig. 2B–D.

**Supplementary File 4**. A list of upregulated genes from the comparison of EWSR1::FLI1 knockdown/rescue to the empty construct.

**Supplementary File 5**. Video tutorial on how to run RNA-SeqEZPZ.

**Supplementary File 6**. Report generated by RNA-SeqEZPZ-NF for analysis of EWSR1::FLI1 and EWSR1::ETV4 knockdown/rescue in the A673 cell line, containing executed commands and CPU and memory usage, providing valuable insights for efficient resource management.

**Supplementary File 7**. Statistical report of the same analysis as presented in Supplementary File 2, but run on OSC.

**Supplementary File 8**. Nextflow report for the analysis of EWSR1::FLI1 knockdown, iLuc control, and HCI 2509 and DMSO control run on AWRI.

**Supplementary File 9**. Nextflow report for the analysis of EWSR1::FLI1 knockdown, iLuc control, and HCI 2509 and DMSO control run on OSC.

**Supplementary File 10**. QC report for the analysis of Nrf2 KO and WT mice run using RaNA-seq.

**Supplementary File 11**. QC report for the analysis of Nrf2 KO and WT mice run using RNA-SeqEZPZ.

**Supplementary File 12**. Raw reads QC report for Nrf2 KO and WT mice samples generated by RNA-SeqEZPZ.

## Abbreviations

AWRI: Abigail Wexner Research Institute; DMSO: dimethyl sulfoxide; FDR: false discovery rate; GLM: generalized linear model; GO: Gene Ontology; HPC: high-performance computing; MAQC: MicroArray Quality Control; OSC: Ohio Supercomputer Center; PC1: principal component 1; PCA: principal component analysis; QC: quality control; RNA-seq: RNA sequencing; WT: wild-type.
