# Supplementary material for: RNA-SeqEZPZ: a point-and-click pipeline for comprehensive transcriptomics analysis with interactive visualizations
Source: Gigascience. 2025 Nov 12;15:giaf133. doi: 10.1093/gigascience/giaf133 (PMC12857227; doi:10.1093/gigascience/giaf133)
Supplement: giaf133_Supplemental_Files [file giaf133_supplemental_files.zip › Supplementary_Figure_13_volcano_HCI2509.pdf]

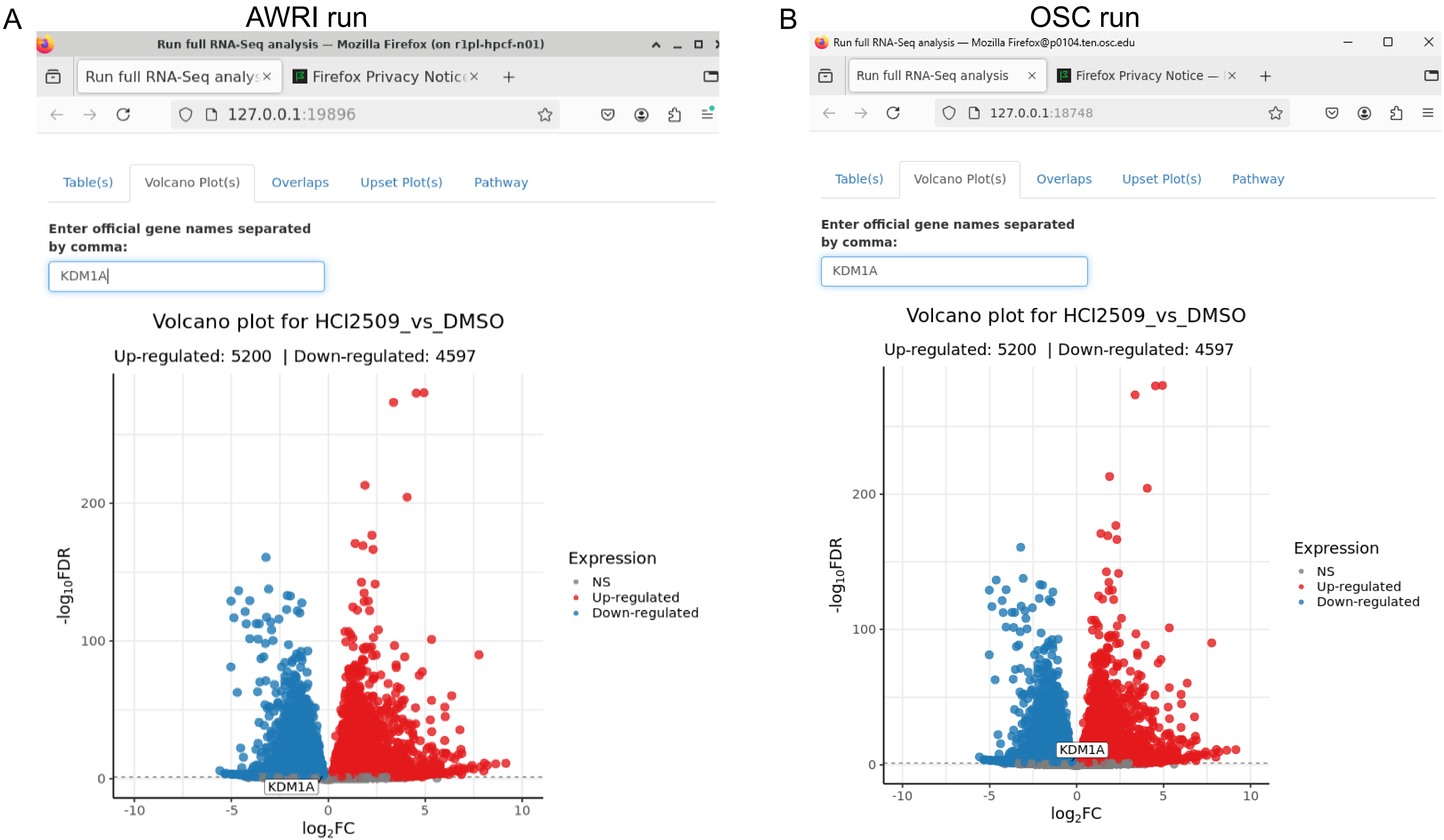
A
AWRI run
Run full RNA-Seq analysis — Mozilla Firefox (on r1pl-hpcf-n01)
Run full RNA-Seq analys
Firefox Privacy Notic
127.0.0.1:19896
Table(s)
Volcano Plot(s)
Overlaps
Upset Plot(s)
Pathway
Enter official gene names separated by comma:
KDM1A
Volcano plot for HCI2509_vs_DMSO
Up-regulated: 5200 | Down-regulated: 4597
-log10FDR
log2FC
KDM1A
Expression
NS
Up-regulated
Down-regulated
B
OSC run
Run full RNA-Seq analysis — Mozilla Firefox@p0104.ten.osc.edu
Run full RNA-Seq analysis
Firefox Privacy Notice —
127.0.0.1:18748
Table(s)
Volcano Plot(s)
Overlaps
Upset Plot(s)
Pathway
Enter official gene names separated by comma:
KDM1A
Volcano plot for HCI2509_vs_DMSO
Up-regulated: 5200 | Down-regulated: 4597
-log10FDR
log2FC
KDM1A
Expression
NS
Up-regulated
Down-regulated
