# Supplementary material for: RNA-SeqEZPZ: a point-and-click pipeline for comprehensive transcriptomics analysis with interactive visualizations
Source: Gigascience. 2025 Nov 12;15:giaf133. doi: 10.1093/gigascience/giaf133 (PMC12857227; doi:10.1093/gigascience/giaf133)
Supplement: giaf133_Supplemental_Files [file giaf133_supplemental_files.zip › Supplementary_Figure_14_PAX3FOXO1_expr.pdf]

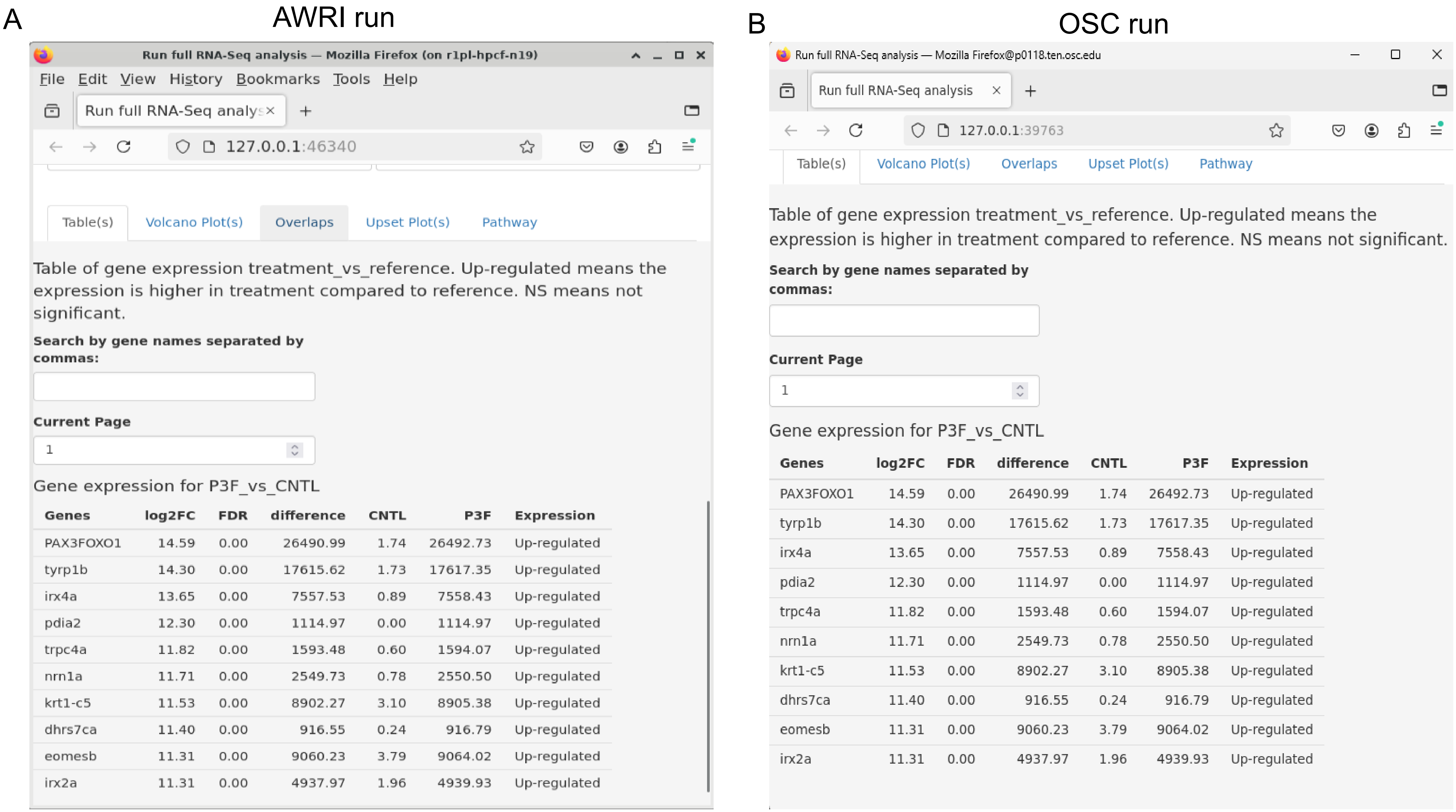

Genes	log2FC	FDR	difference	CNTL	P3F	Expression
PAX3FOXO1	14.59	0.00	26490.99	1.74	26492.73	Up-regulated
tyrp1b	14.30	0.00	17615.62	1.73	17617.35	Up-regulated
irx4a	13.65	0.00	7557.53	0.89	7558.43	Up-regulated
pdia2	12.30	0.00	1114.97	0.00	1114.97	Up-regulated
trpc4a	11.82	0.00	1593.48	0.60	1594.07	Up-regulated
nrn1a	11.71	0.00	2549.73	0.78	2550.50	Up-regulated
krt1-c5	11.53	0.00	8902.27	3.10	8905.38	Up-regulated
dhrs7ca	11.40	0.00	916.55	0.24	916.79	Up-regulated
eomesb	11.31	0.00	9060.23	3.79	9064.02	Up-regulated
irx2a	11.31	0.00	4937.97	1.96	4939.93	Up-regulated

Genes	log2FC	FDR	difference	CNTL	P3F	Expression
PAX3FOXO1	14.59	0.00	26490.99	1.74	26492.73	Up-regulated
tyrp1b	14.30	0.00	17615.62	1.73	17617.35	Up-regulated
irx4a	13.65	0.00	7557.53	0.89	7558.43	Up-regulated
pdia2	12.30	0.00	1114.97	0.00	1114.97	Up-regulated
trpc4a	11.82	0.00	1593.48	0.60	1594.07	Up-regulated
nrn1a	11.71	0.00	2549.73	0.78	2550.50	Up-regulated
krt1-c5	11.53	0.00	8902.27	3.10	8905.38	Up-regulated
dhrs7ca	11.40	0.00	916.55	0.24	916.79	Up-regulated
eomesb	11.31	0.00	9060.23	3.79	9064.02	Up-regulated
irx2a	11.31	0.00	4937.97	1.96	4939.93	Up-regulated
