# Supplementary material for: RNA-SeqEZPZ: a point-and-click pipeline for comprehensive transcriptomics analysis with interactive visualizations
Source: Gigascience. 2025 Nov 12;15:giaf133. doi: 10.1093/gigascience/giaf133 (PMC12857227; doi:10.1093/gigascience/giaf133)
Supplement: giaf133_Supplemental_Files [file giaf133_supplemental_files.zip › Supplementary_File_10_Nrf2_RaNAseq_QC.pdf]

Quality Control

RaNA-seq

2025-06-18 17:22:12

Samples

```
##  id      name
##   1      WT_1
##   2      WT_2
##   3 Nrf2_KO_1
##   4 Nrf2_KO_2
```

Table 1 Table of samples.

Expression Value

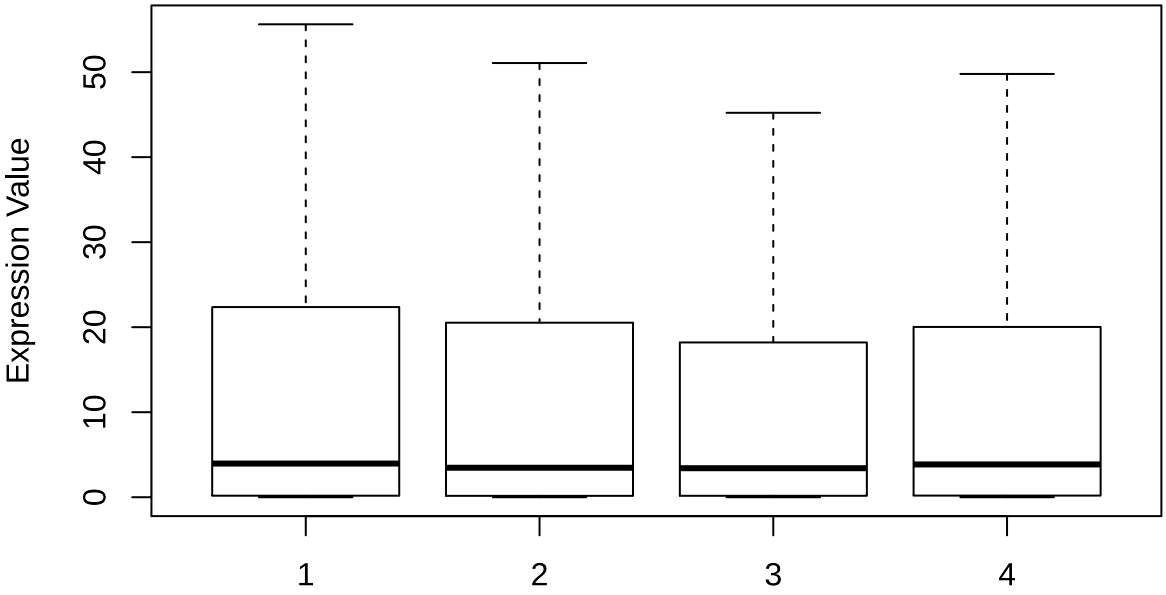

Figure 1 Box-and-whisker plots (boxplots) of the expression values (normalized as TPMs) for each sample.

ExpressedGenes

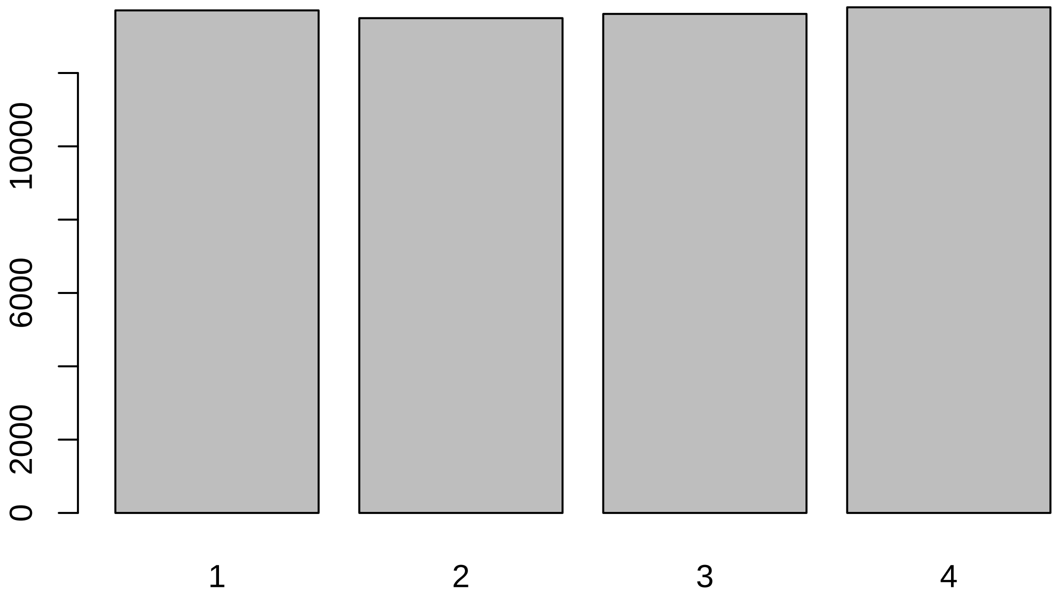

Figure 2 Barplot with the estimated expressed genes per sample.

Heatmap

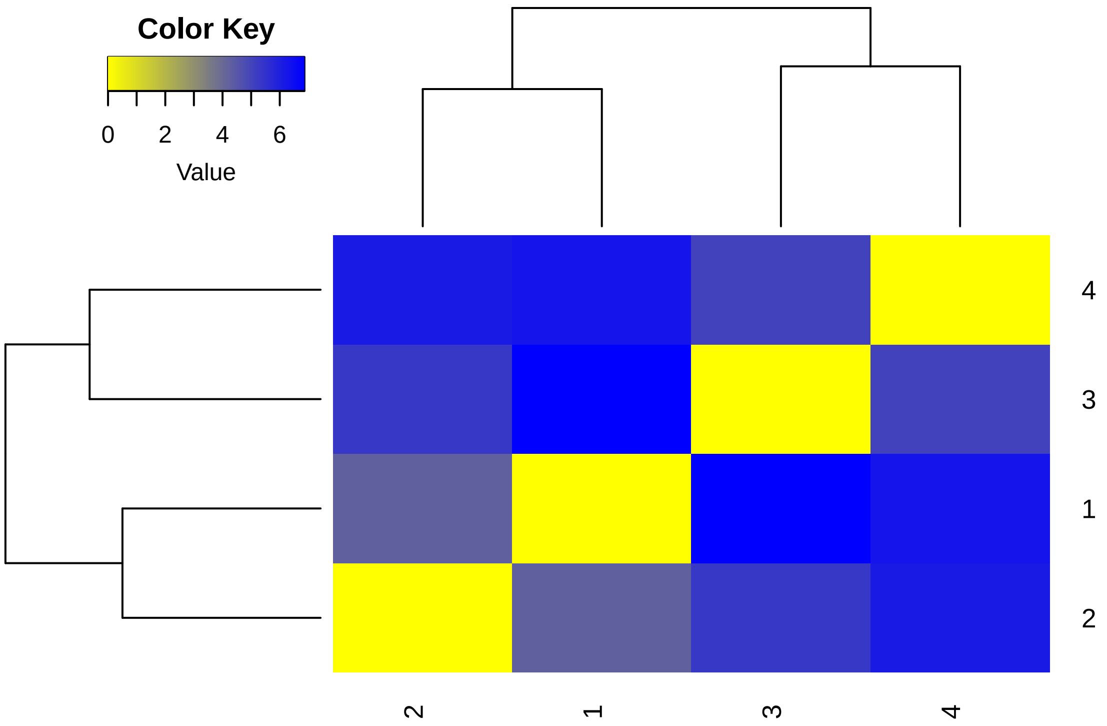

Figure 3 Heatmap of expression similarity between samples. It shows the distance between samples calculating the SERE coefficient (Schulze et al. 2012) between each pair of samples. Similar samples have lower values in the graph.

PCA plot

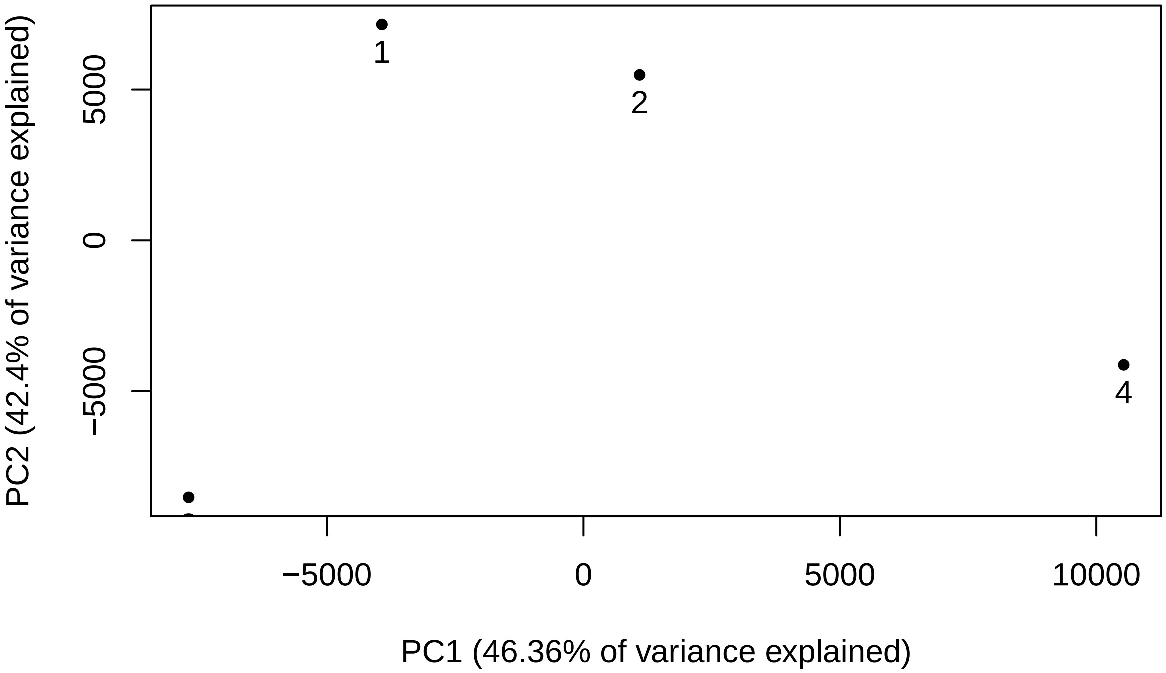

Figure 4 PCA plot of samples. It shows the two first PCs (principal components) values of each sample.

Methods

SERE coefficent was used as distance between samples on Figure 3 (Schulze et al. 2012). Graphs were generated using R (R Development Core Team 2011) and RJSplot (Barrios and Prieto 2018).

References

Barrios, David, and Carlos Prieto. 2018. "RJSplot: Interactive Graphs with R." *Molecular Informatics* 37 (3). https://doi.org/10.1002/minf.201700090.

R Development Core Team, R. 2011. *R: A Language and Environment for Statistical Computing.* Vol. 1. 2.11.1. https://doi.org/10.1007/978-3-540-74686-7.

Schulze, Stefan K., Rahul Kanwar, Meike Gölzenleuchter, Terry M. Therneau, and Andreas S. Beutler. 2012. "SERE: Single-parameter quality control and sample comparison for RNA-Seq." *BMC Genomics* 13 (1). https://doi.org/10.1186/1471-2164-13-524.
