# Supplementary material for: RNA-SeqEZPZ: a point-and-click pipeline for comprehensive transcriptomics analysis with interactive visualizations
Source: Gigascience. 2025 Nov 12;15:giaf133. doi: 10.1093/gigascience/giaf133 (PMC12857227; doi:10.1093/gigascience/giaf133)
Supplement: giaf133_Supplemental_Files [file giaf133_supplemental_files.zip › Supplementary_Table_S1_comparison_other_pipelines.docx]

**Supplementary Table S1**: A comparison of existing RNA-Seq pipelines with RNA-SeqEZPZ

|  | **Ease of installation** | **Ease of use** | **Workflow management systems** | **Access to full source code** | **Statistical method for differential analysis** | **Features** |
| --- | --- | --- | --- | --- | --- | --- |
| **RNA-SeqEZPZ** | ✔  All software is packaged into a Singularity container. | ✔  Users only need to run one command line to invoke an interface to run the entire analysis. | ✔  Nextflow^1^ is used | ✔ | GLM (DESeq2^2^) | ✔Raw reads QC  ✔Differential expression  ✔Enrichment analysis  ✔Comparative analysis across different conditions  ✖Gene regulatory network |
| **bulkAnalyseR (2023)**^3^ | ✖  Requires installation of dozens of R packages. | ✖  Requires pre-processing of FASTQ files expression matrix before any analysis can be run. | ✖ | ✔ | GLM (edgeR^4^ or DESeq2^2^) | ✖Raw reads QC  ✔Differential expression  ✔Enrichment analysis  ✔ Comparative analysis across different sets of DEGs (up to two)  ✔Gene regulatory network |
| **ROGUE**  **(2023)**^5^ | ✔  No installation required if using web version | ✖  Requires FASTQ files to be pre-processed as count data. | ✖ | ✔ | GLM (edgeR^4^ or DESeq2^2^) | ✖Raw reads QC  ✔Differential expression  ✔Enrichment analysis  ✔Comparative analysis across different sets of DEGs  ✖Gene regulatory network |
| **ENCODE**  **RNA-Seq**  **(2023)**^6^ | ✔  All software is packaged into a Singularity container. | ✖  No differential gene analysis or interactive visualization | ✔  WDL^7^ is used | ✔ | NA | ✔Raw reads QC  ✖Differential expression  ✖Enrichment analysis  ✖Comparative analysis across different conditions  ✖Gene regulatory network |
| **RASflow (2020)**^8^ | ✔  All software is packaged into a Docker container. | ✖  No interactive visualization | ✔  Snakemake^9^ is used | ✔ | GLM (edgeR^4^ or DESeq2^2^) | ✔Raw reads QC  ✔Differential expression  ✖Enrichment analysis  ✖Comparative analysis across different conditions |
| **RaNA-Seq**  **(2020)**^10^ | ✔  No installation required. Everything is done on their server. | ✔ | ✖ | ✖  Everything is done on their server. No access to full source code. | GLM (edgeR^4^ or DESeq2^2^) or linear model (limma^11^) | ✖ Raw reads QC  ✔Differential expression  ✔Enrichment analysis  ✔ Comparative analysis across different conditions |
| **nf-core/rnaseq**  **(2020)**^12^ | ✔  All software is packaged into a Singularity^13^ container. | ✖  To do differential gene analysis and interactive visualization a different pipeline (nf-core/differentialabundance) needs to be run separately. | ✔  Nextflow^1^ is used | ✔ | NA | ✔Raw reads QC  ✖Differential expression  ✖Enrichment analysis  ✖Comparative analysis across different conditions  ✖Gene regulatory network  ✖ No interactive visualization |

GLM: Generalized Linear Model, DEGs: Differentially Expressed Genes.

# References

1. DI Tommaso, P. *et al.* Nextflow enables reproducible computational workflows. *Nature Biotechnology 2017 35:4* 35, 316–319 (2017).

2. Love, M. I., Huber, W. & Anders, S. Moderated estimation of fold change and dispersion for RNA-seq data with DESeq2. *Genome Biol* 15, 550 (2014).

3. Moutsopoulos, I., Williams, E. C. & Mohorianu, I. I. bulkAnalyseR: an accessible, interactive pipeline for analysing and sharing bulk multi-modal sequencing data. *Brief Bioinform* 24, 1–7 (2023).

4. Robinson, M. D., McCarthy, D. J. & Smyth, G. K. edgeR: a Bioconductor package for differential expression analysis of digital gene expression data. *Bioinformatics* 26, 139–140 (2010).

5. Farrel, A. *et al.* ROGUE: an R Shiny app for RNA sequencing analysis and biomarker discovery. *BMC Bioinformatics* 24, 1–13 (2023).

6. Hitz, B. C. *et al.* The ENCODE Uniform Analysis Pipelines. *bioRxiv* (2023) doi:10.1101/2023.04.04.535623.

7. Voss, K. *et al.* Full-stack genomics pipelining with GATK4 + WDL + Cromwell. *F1000Res* 6, (2017).

8. Zhang, X. & Jonassen, I. RASflow: An RNA-Seq analysis workflow with Snakemake. *BMC Bioinformatics* 21, 1–9 (2020).

9. Köster, J. & Rahmann, S. Snakemake—a scalable bioinformatics workflow engine. *Bioinformatics* 28, 2520–2522 (2012).

10. Prieto, C. & Barrios, D. RaNA-Seq: interactive RNA-Seq analysis from FASTQ files to functional analysis. *Bioinformatics* 36, 1955–1956 (2020).

11. Ritchie, M. E. *et al.* limma powers differential expression analyses for RNA-sequencing and microarray studies. *Nucleic Acids Res* 43, e47–e47 (2015).

12. Ewels, P. A. *et al.* The nf-core framework for community-curated bioinformatics pipelines. *Nature Biotechnology 2020 38:3* 38, 276–278 (2020).

13. Kurtzer, G. M., Sochat, V. & Bauer, M. W. Singularity: Scientific containers for mobility of compute. *PLoS One* 12, e0177459 (2017).
