# Supplementary figures and images for: RNA-SeqEZPZ: a point-and-click pipeline for comprehensive transcriptomics analysis with interactive visualizations
Source: Gigascience. 2025 Nov 12;15:giaf133. doi: 10.1093/gigascience/giaf133 (PMC12857227; doi:10.1093/gigascience/giaf133)

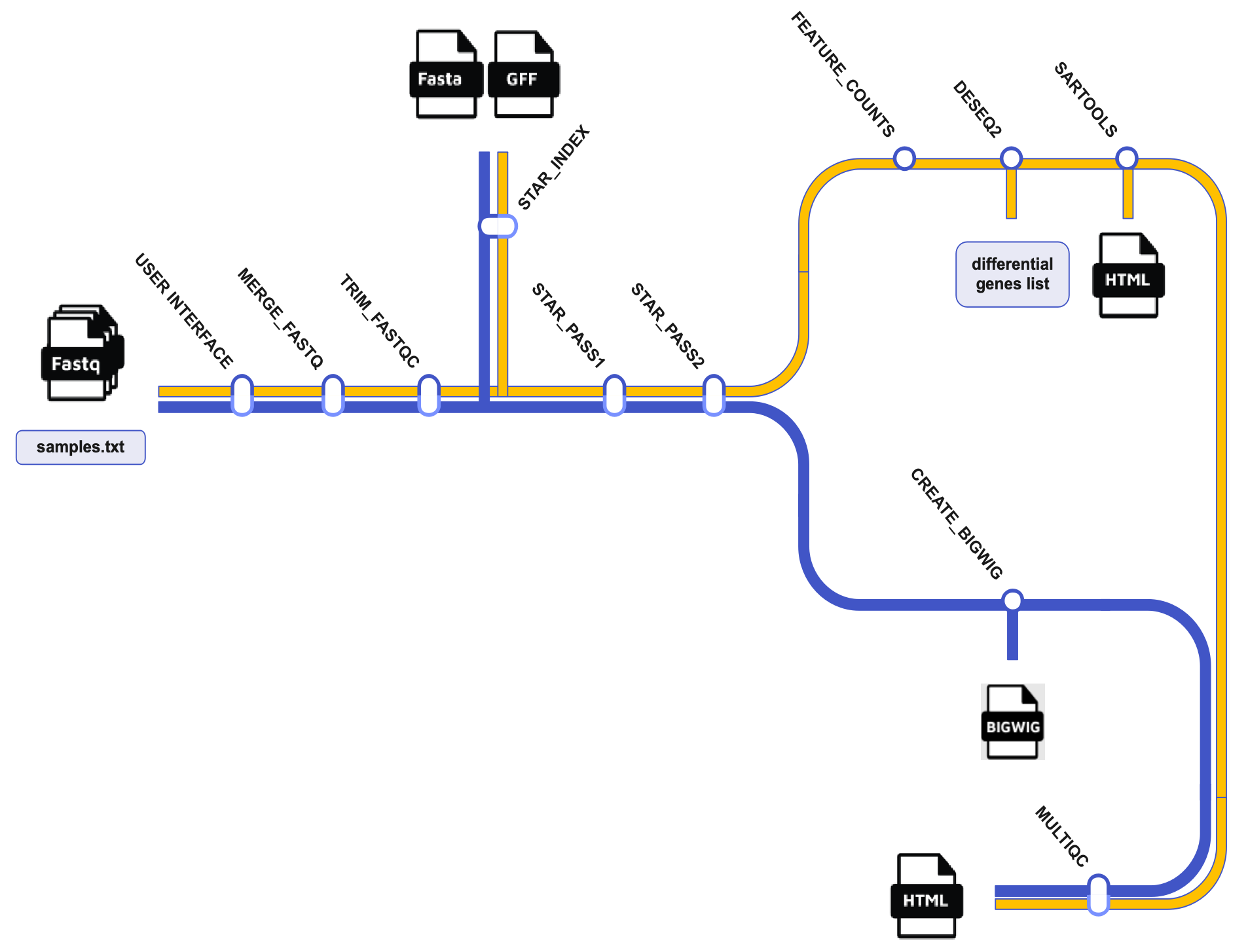

Supplement: giaf133_Supplemental_Files [file giaf133_supplemental_files.zip › Supplementary Figure 1.png]

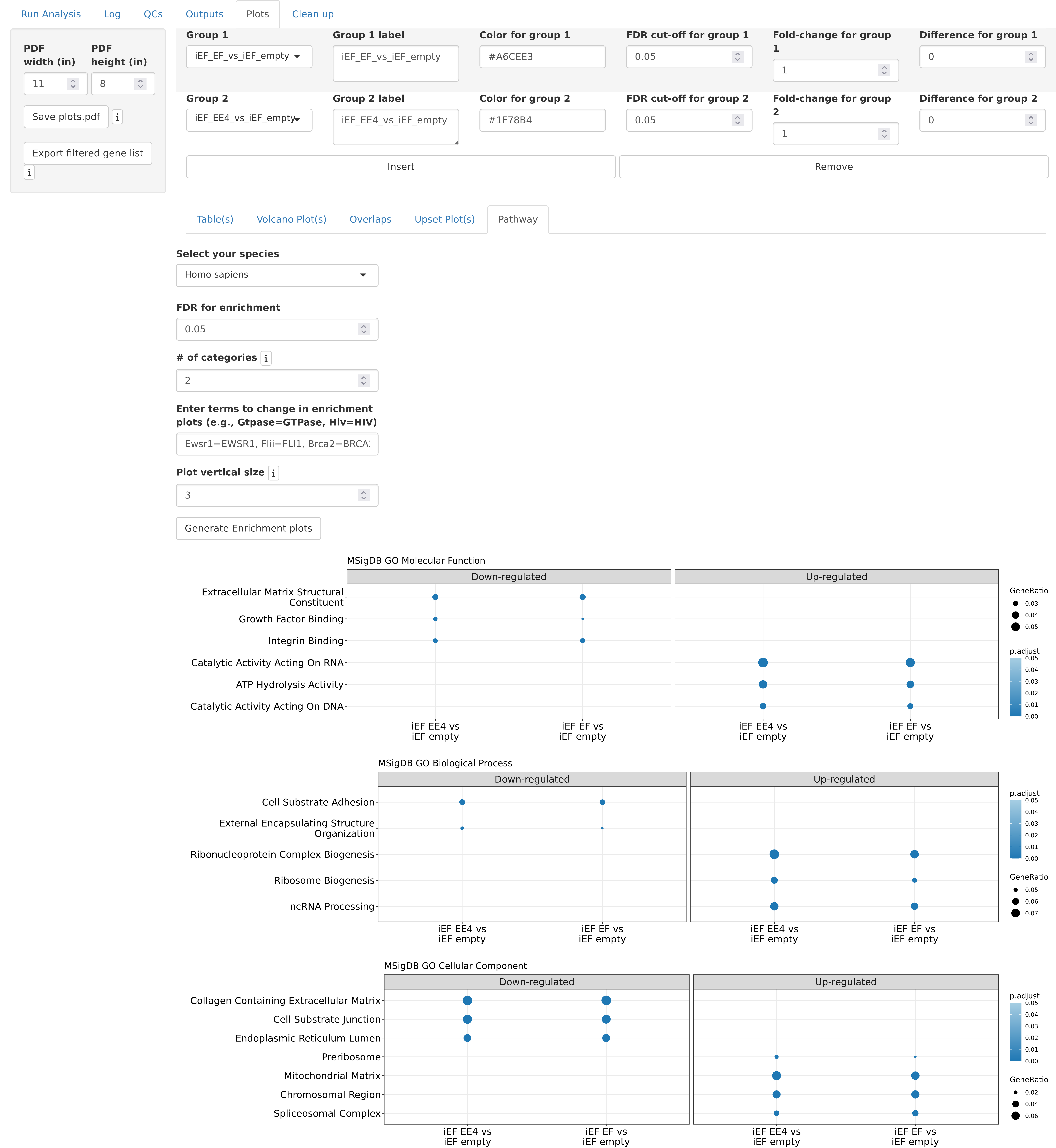

Supplement: giaf133_Supplemental_Files [file giaf133_supplemental_files.zip › Supplementary Figure 10.png]

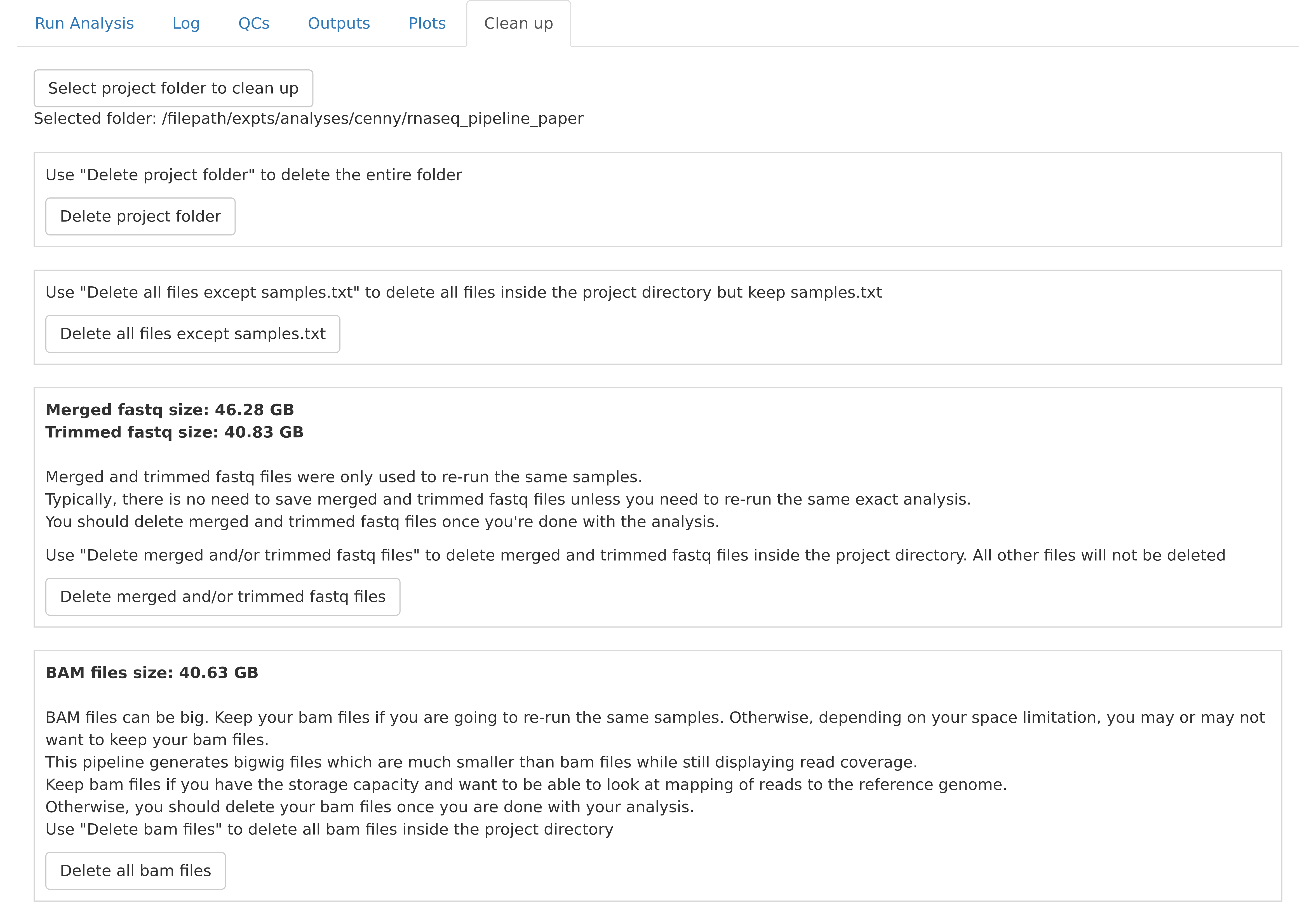

Supplement: giaf133_Supplemental_Files [file giaf133_supplemental_files.zip › Supplementary Figure 11.png]

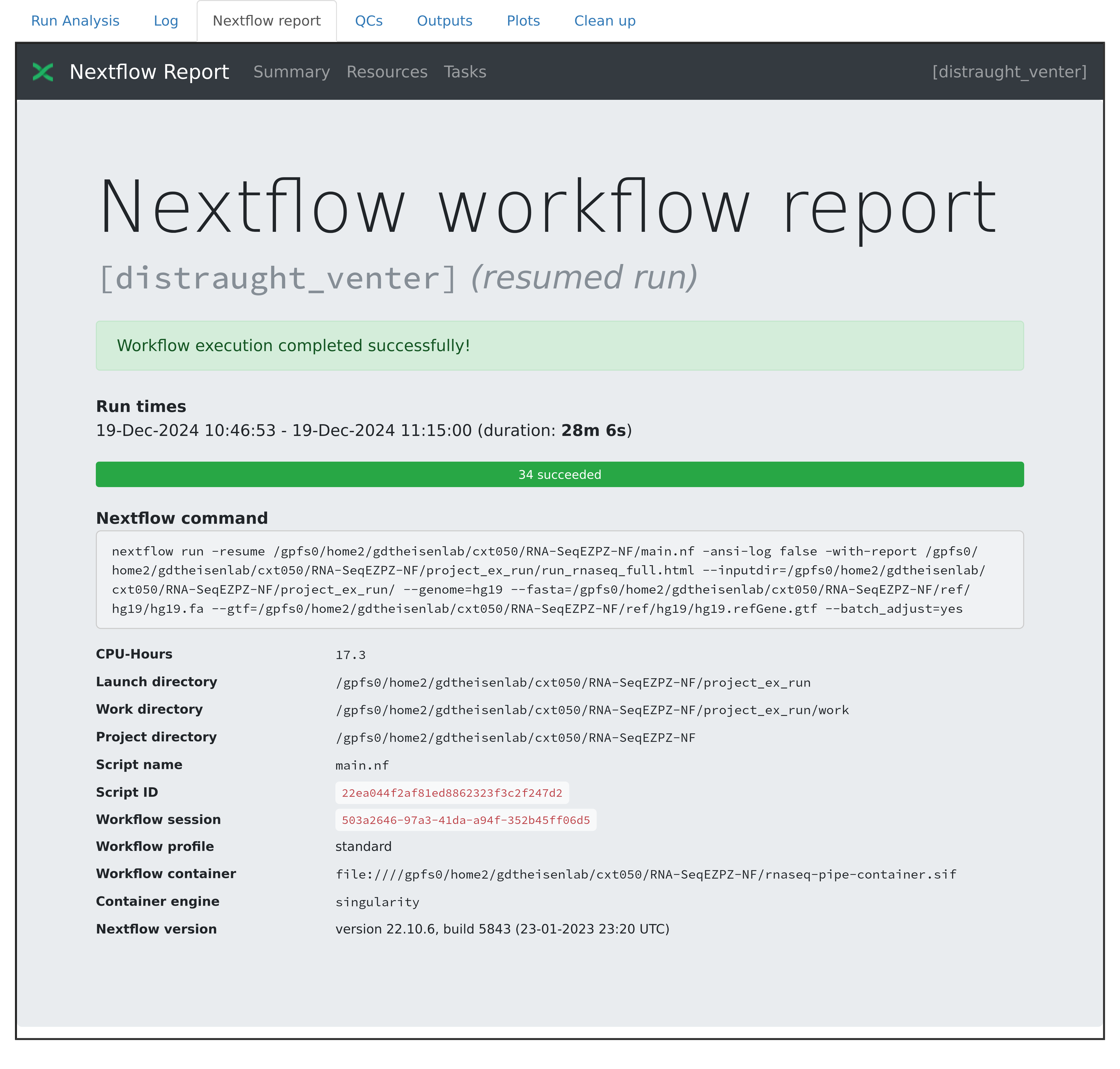

Supplement: giaf133_Supplemental_Files [file giaf133_supplemental_files.zip › Supplementary Figure 12.png]

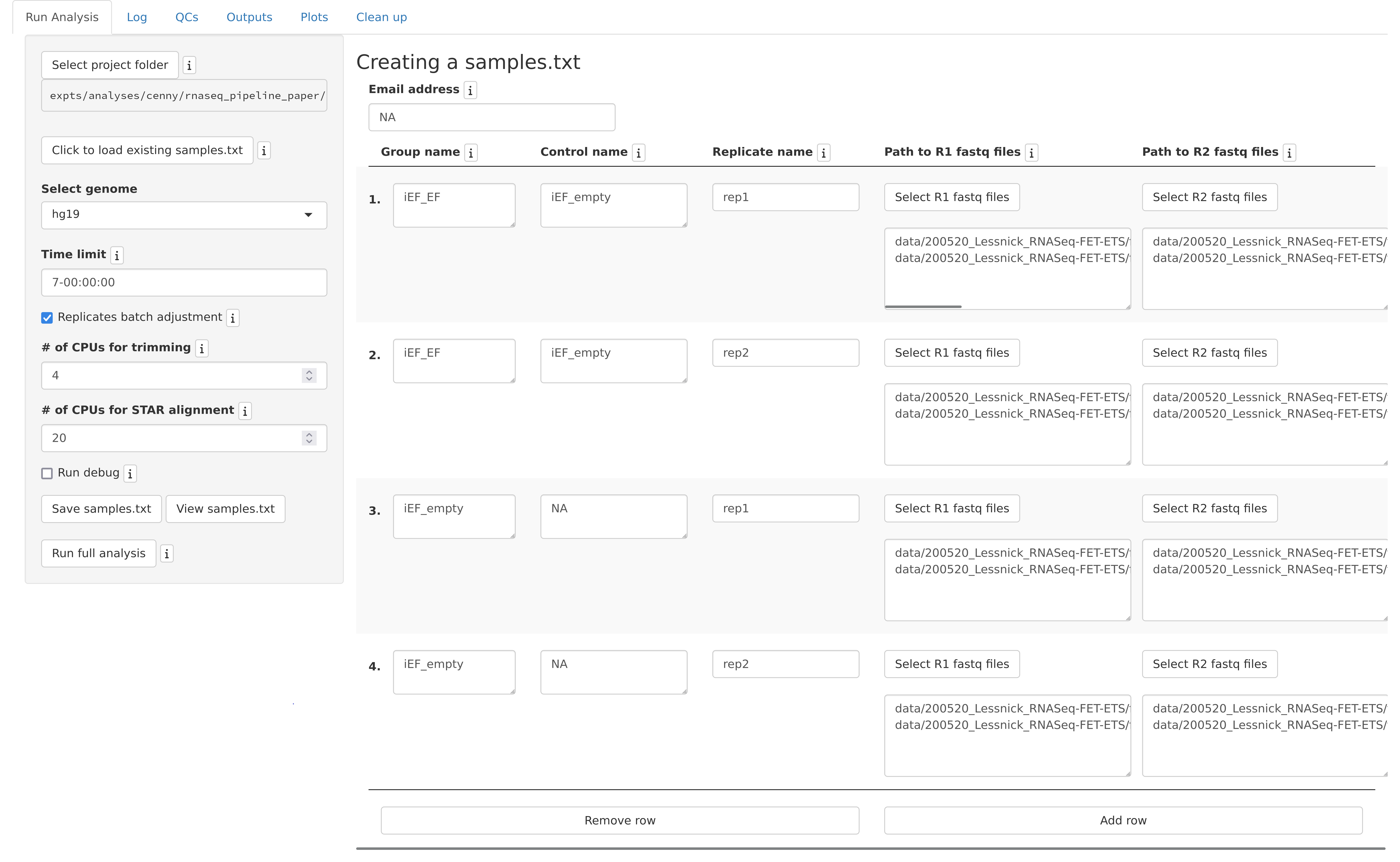

Supplement: giaf133_Supplemental_Files [file giaf133_supplemental_files.zip › Supplementary Figure 2.png]

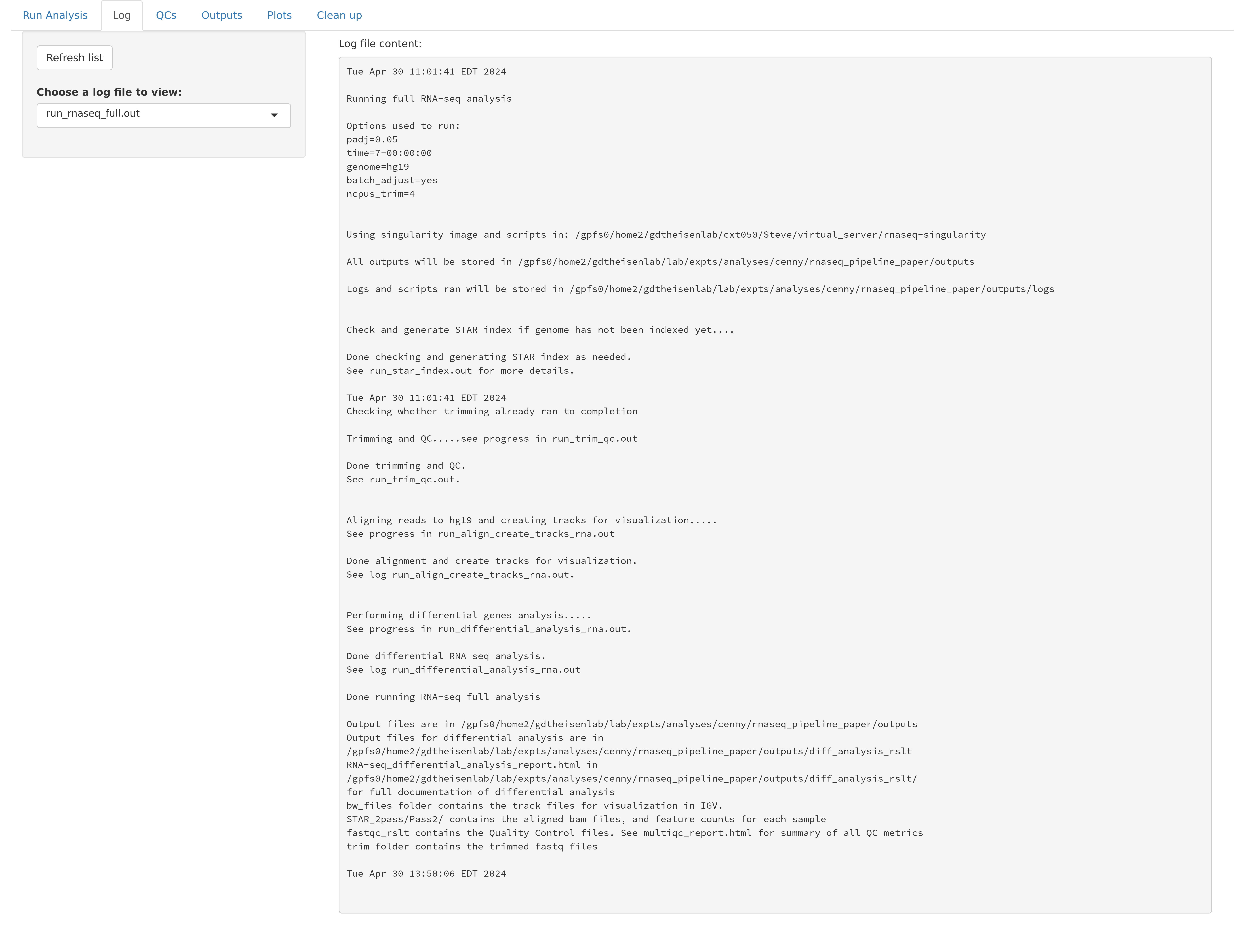

Supplement: giaf133_Supplemental_Files [file giaf133_supplemental_files.zip › Supplementary Figure 3.png]

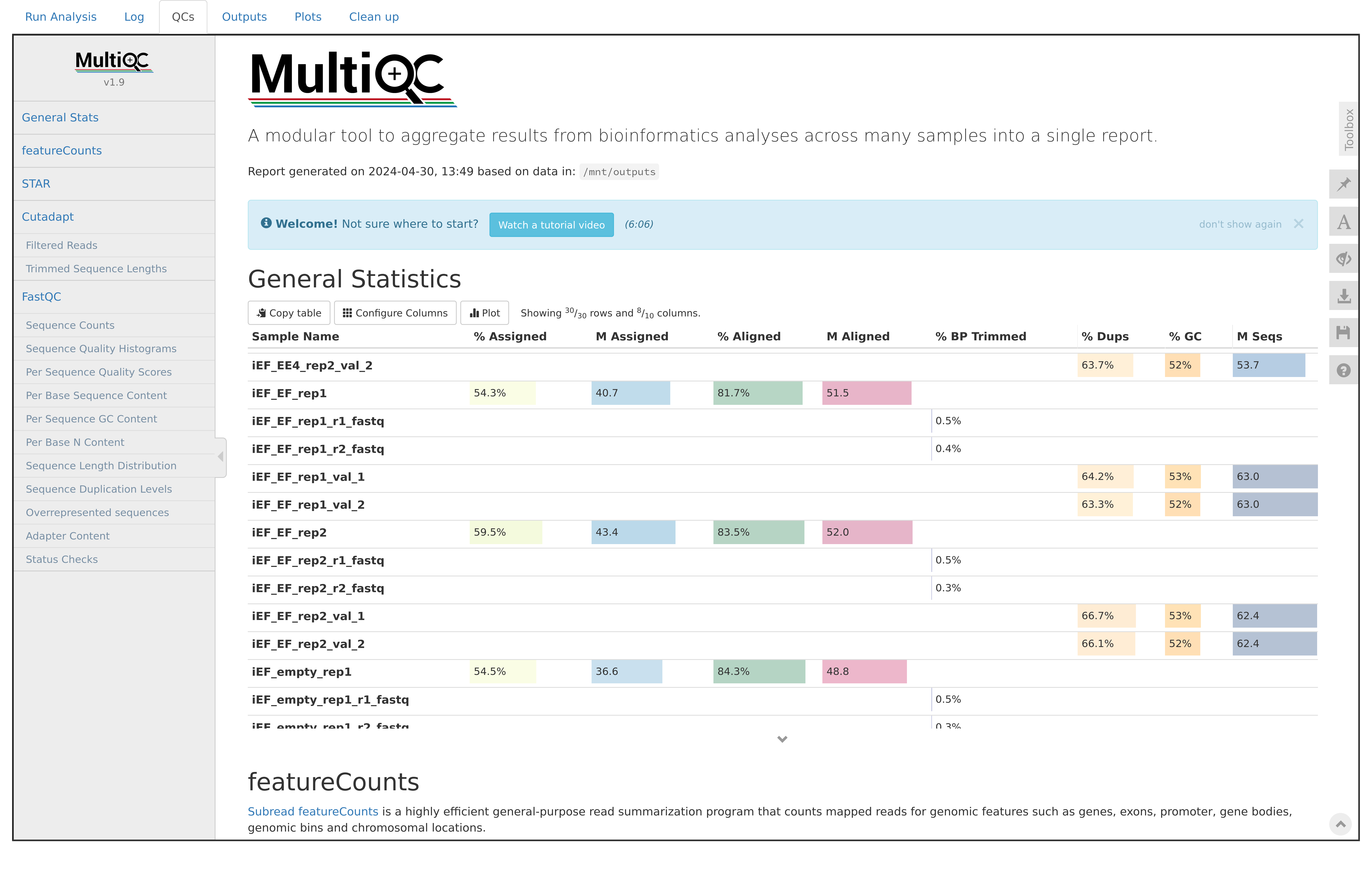

Supplement: giaf133_Supplemental_Files [file giaf133_supplemental_files.zip › Supplementary Figure 4.png]

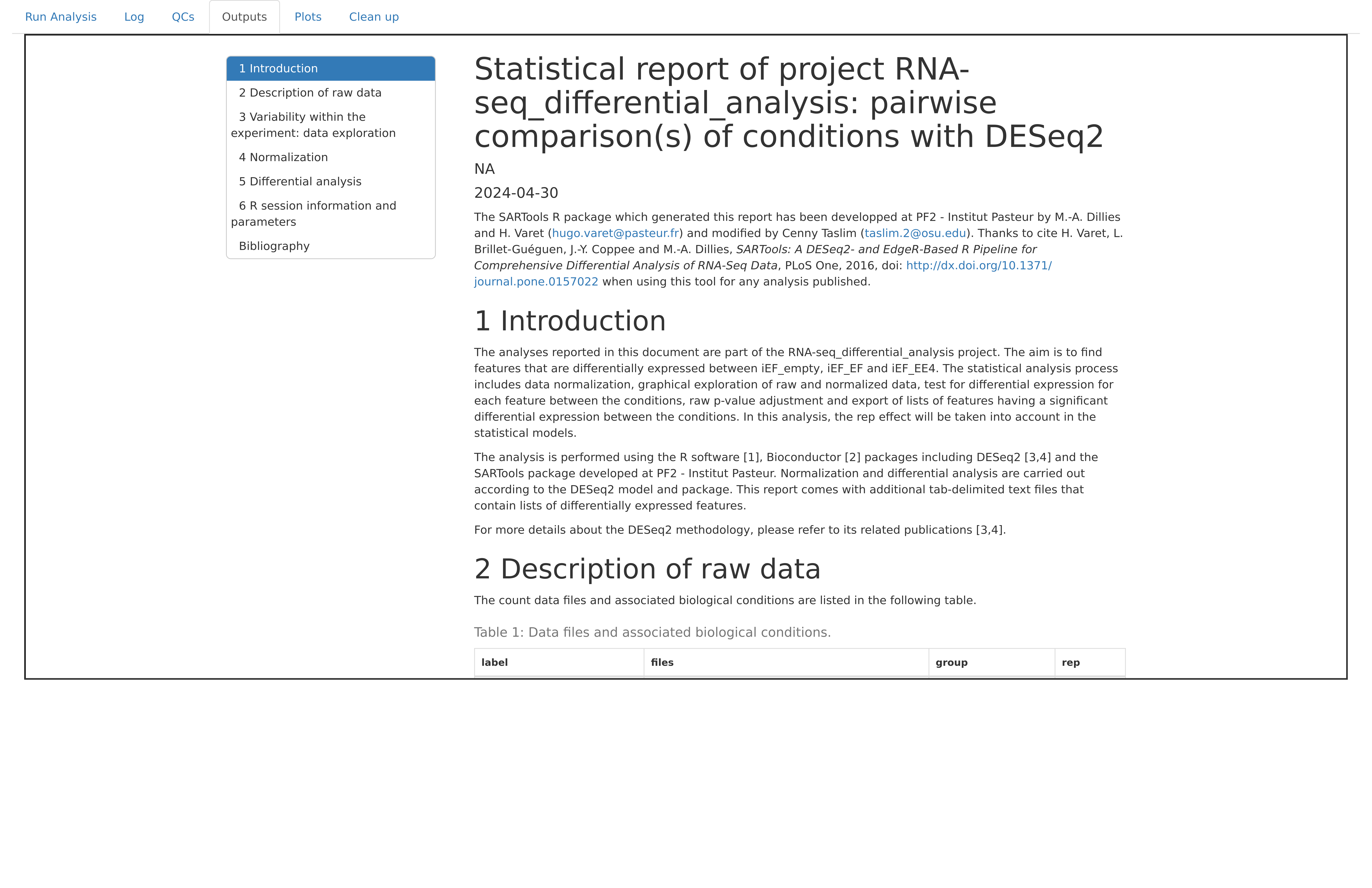

Supplement: giaf133_Supplemental_Files [file giaf133_supplemental_files.zip › Supplementary Figure 5.png]

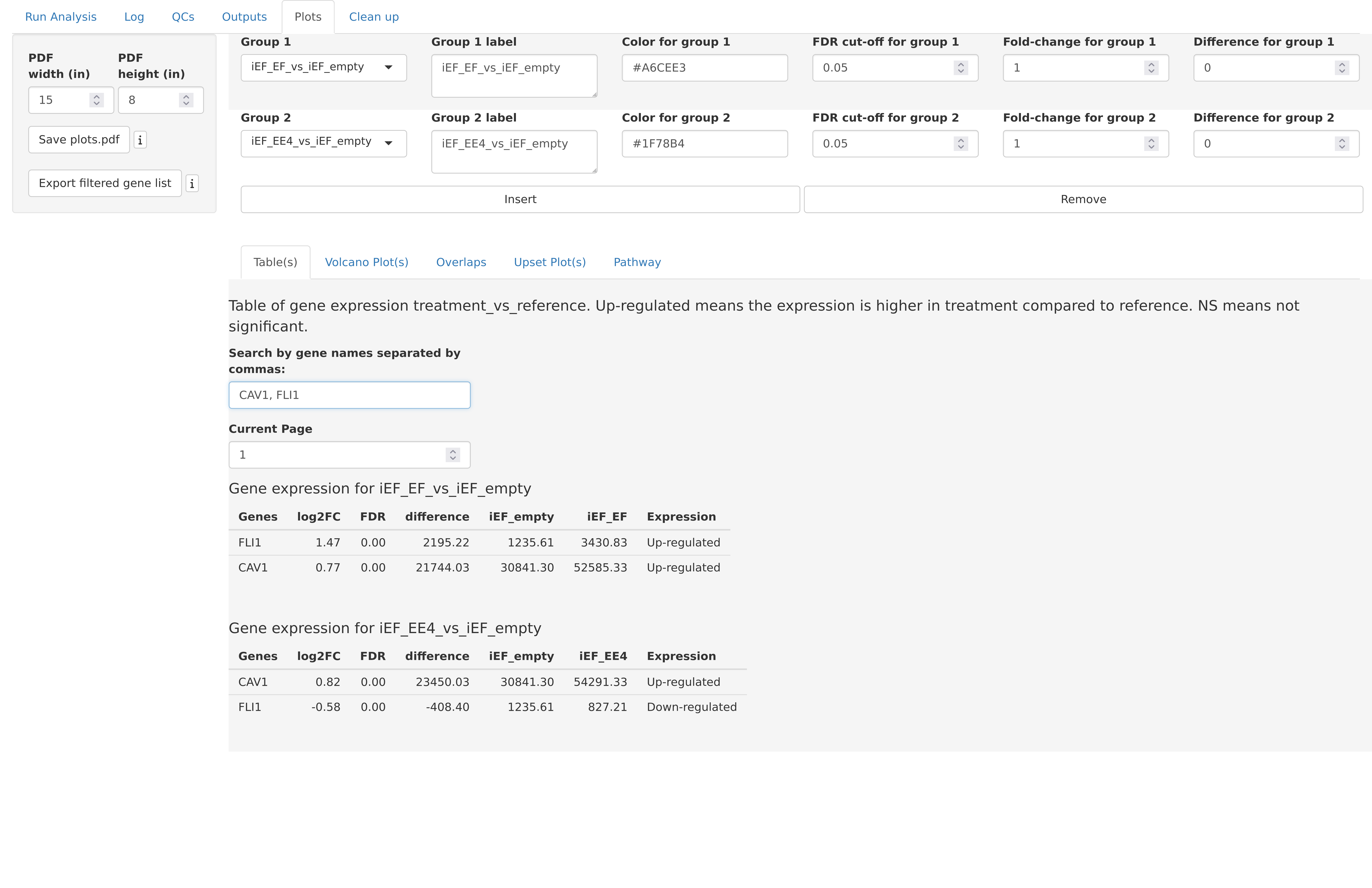

Supplement: giaf133_Supplemental_Files [file giaf133_supplemental_files.zip › Supplementary Figure 6.png]

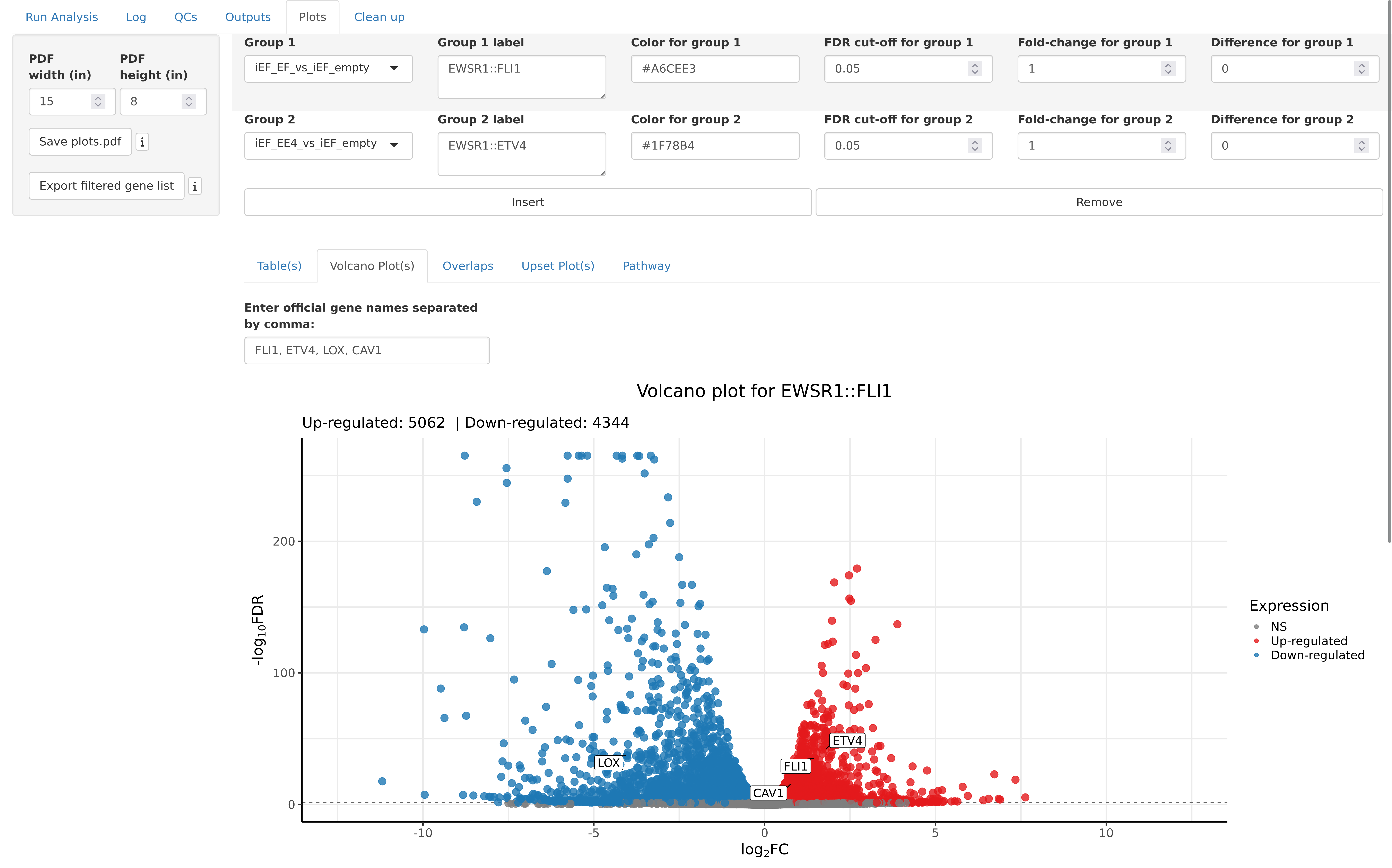

Supplement: giaf133_Supplemental_Files [file giaf133_supplemental_files.zip › Supplementary Figure 7.png]

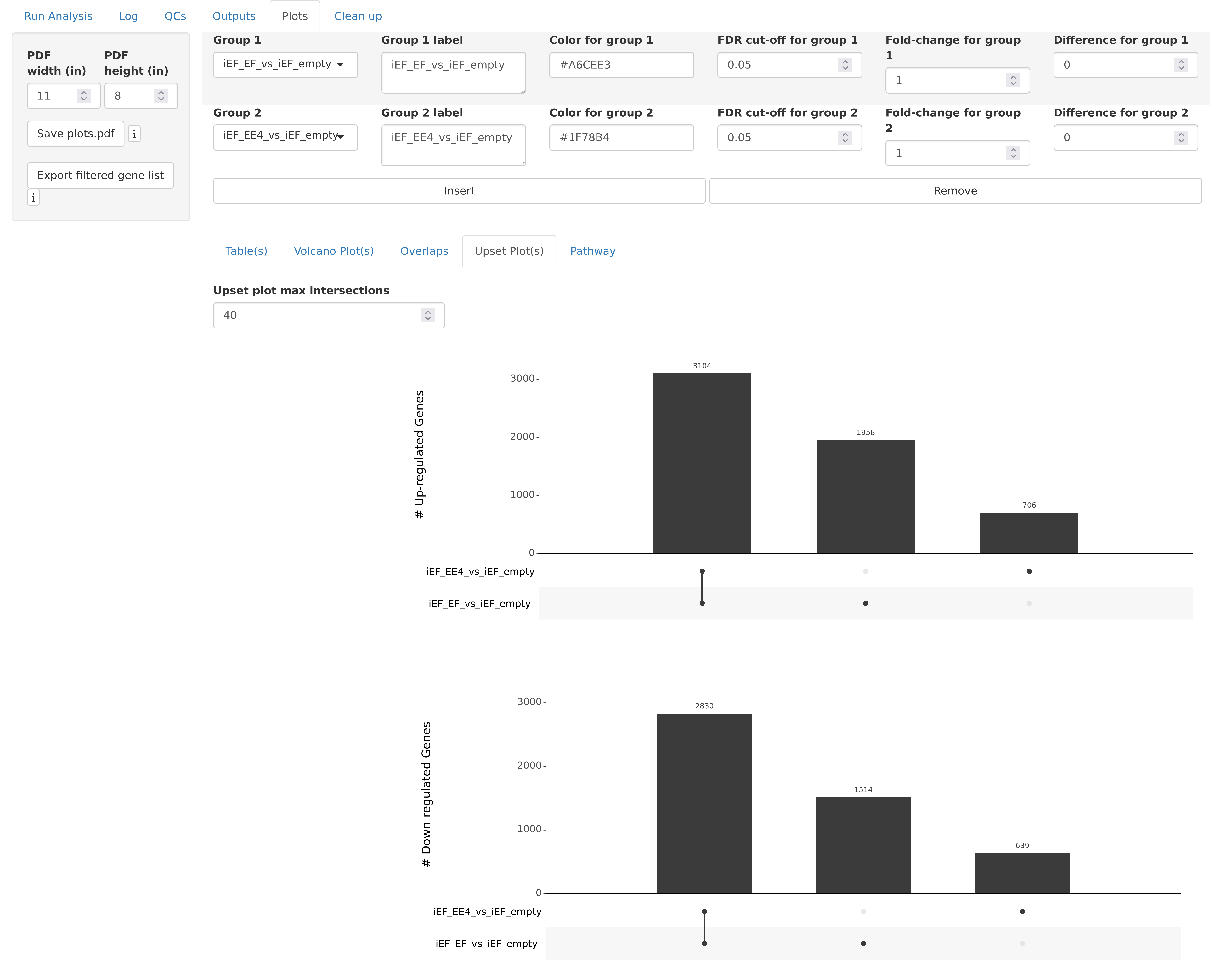

Supplement: giaf133_Supplemental_Files [file giaf133_supplemental_files.zip › Supplementary Figure 8.png]

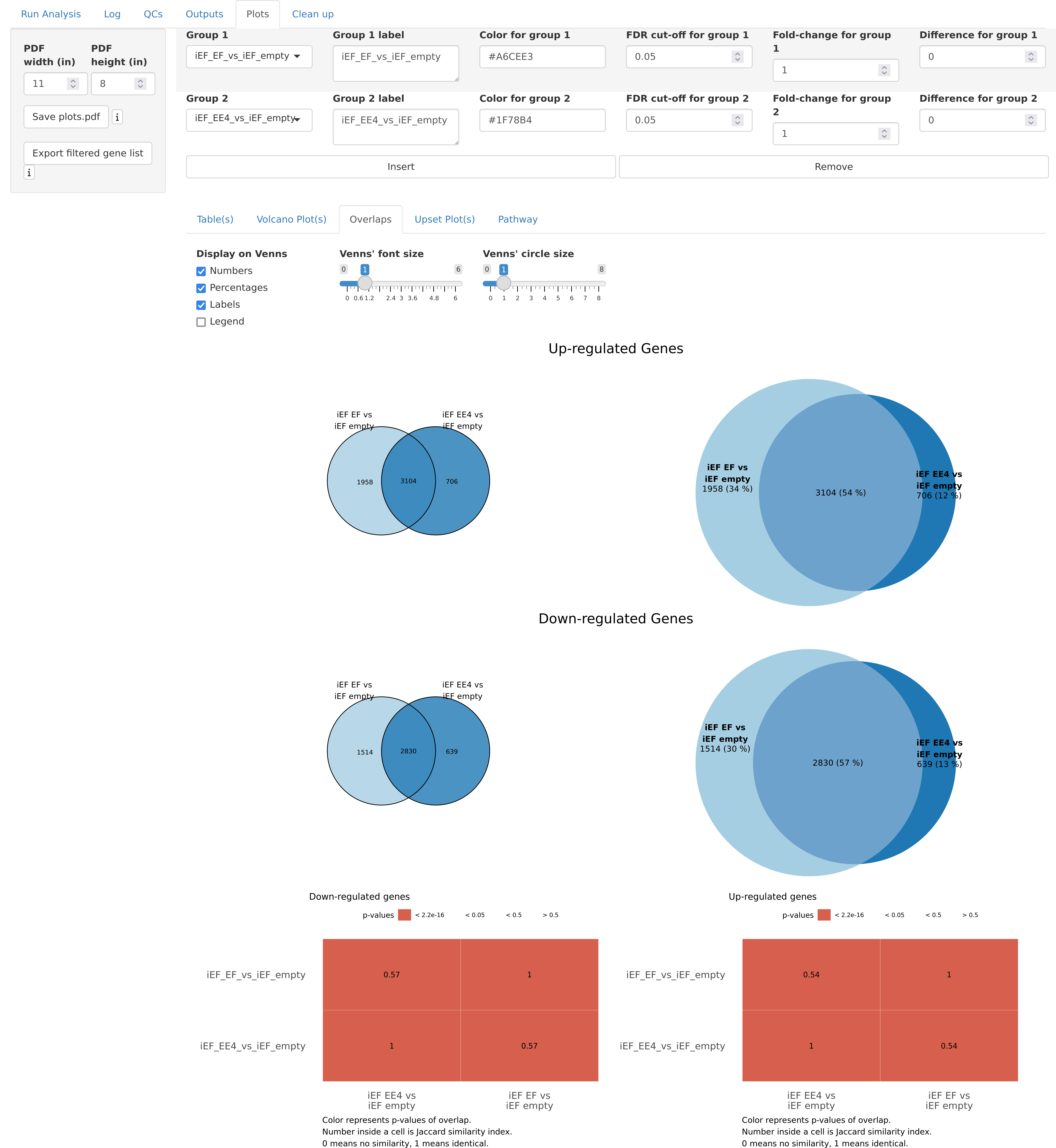

Supplement: giaf133_Supplemental_Files [file giaf133_supplemental_files.zip › Supplementary Figure 9.png]

**A** Before batch adjustment

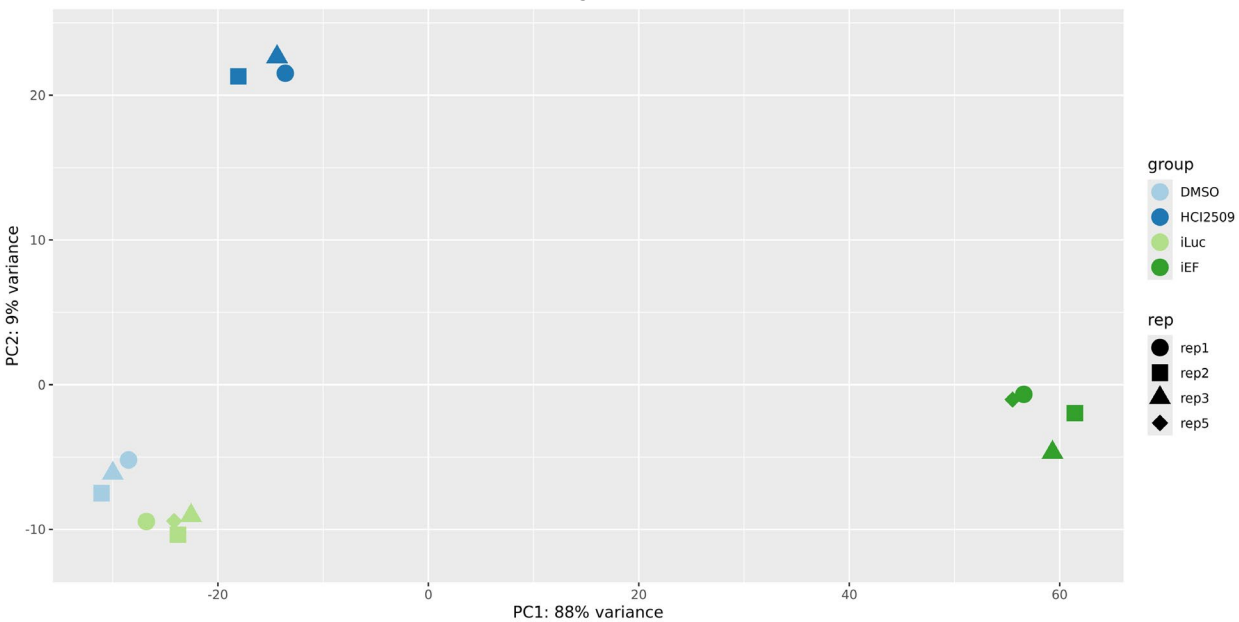

**B** After batch adjustment

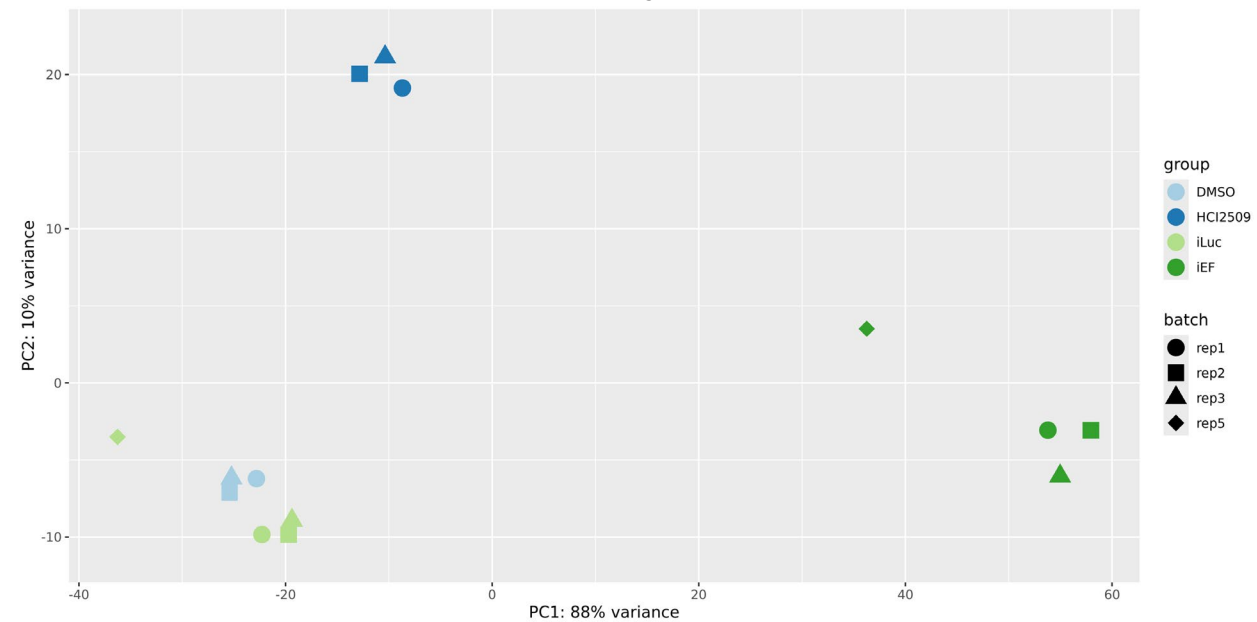

Supplement: giaf133_Supplemental_Files [file giaf133_supplemental_files.zip › Supplementary_Figure_15_PCA_HCI2509.pdf]

A

## RaNA-seq

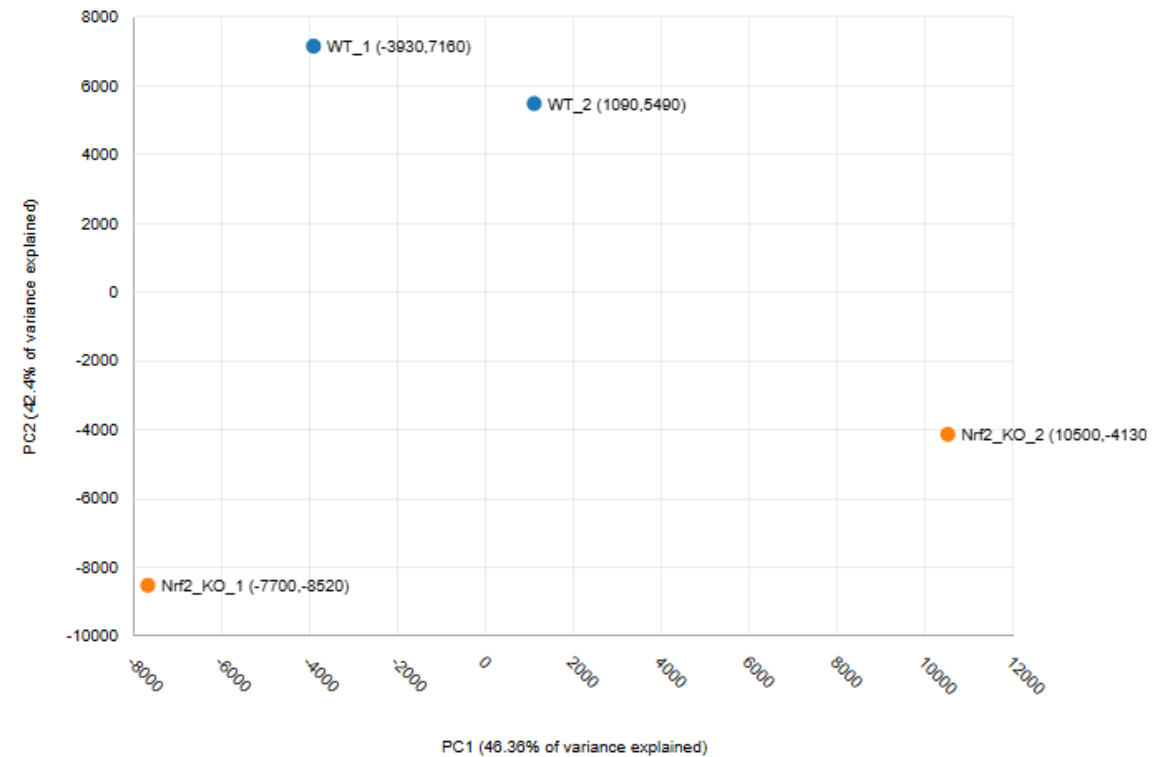

B

## RNA-SeqEZPZ

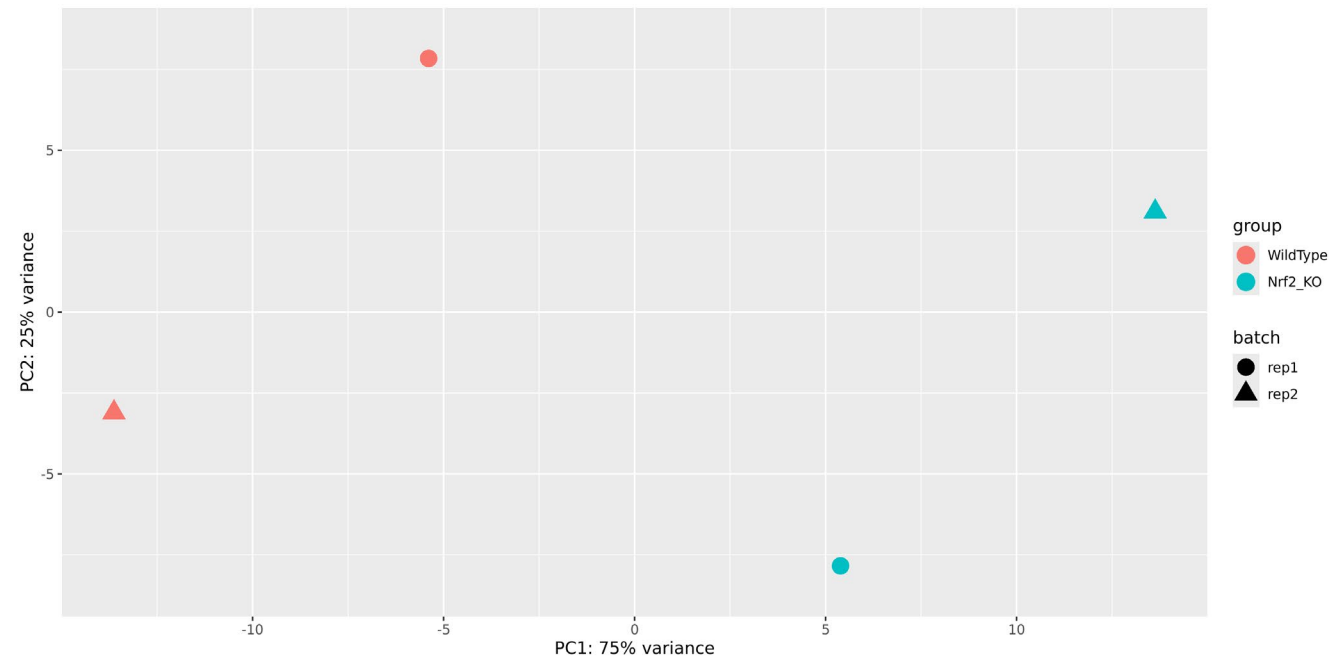

Supplement: giaf133_Supplemental_Files [file giaf133_supplemental_files.zip › Supplementary_Figure_16_PCA_Nrf2.pdf]

A

## RaNA-seq

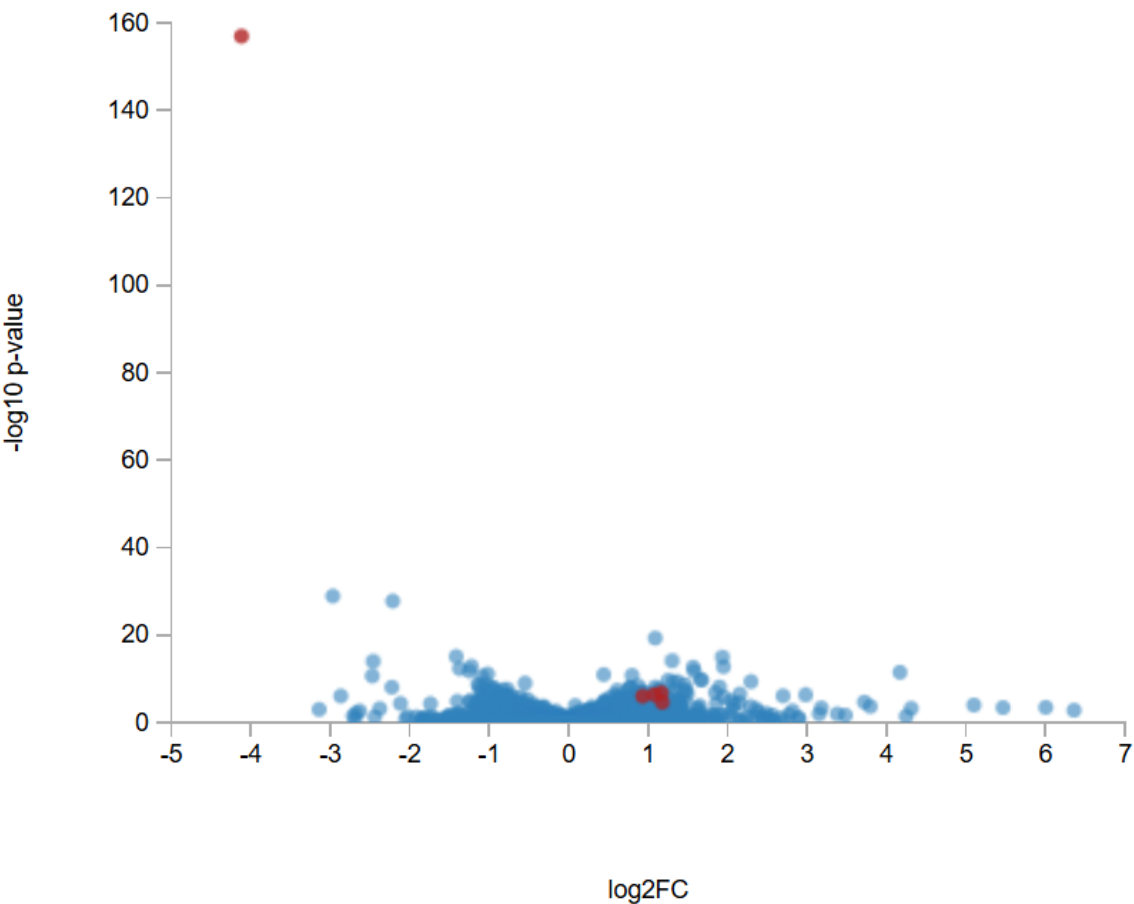

B

## RNA-SeqEZPZ

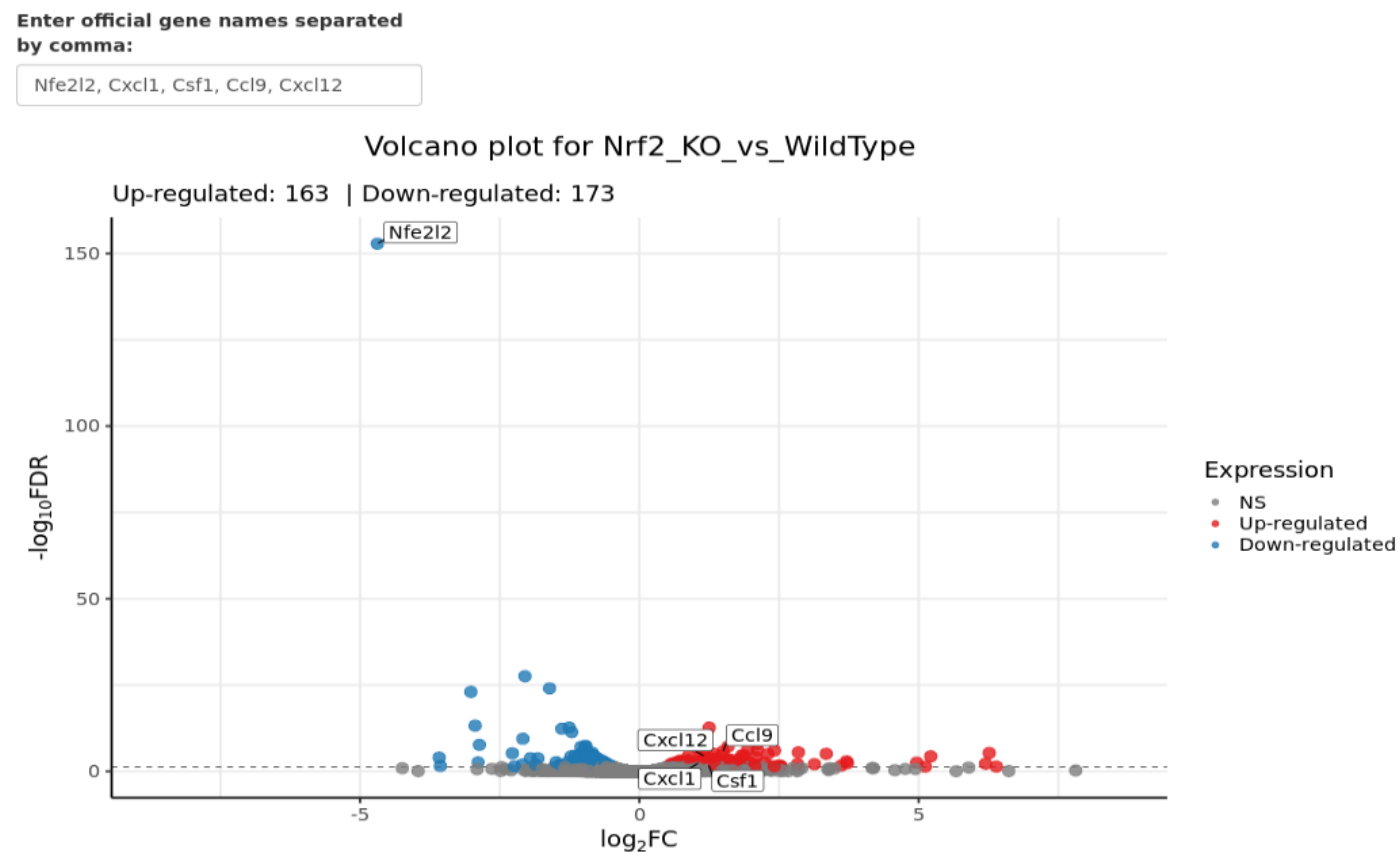

Supplement: giaf133_Supplemental_Files [file giaf133_supplemental_files.zip › Supplementary_Figure_17_volcano_Nrf2.pdf]

A

RaNA-seq

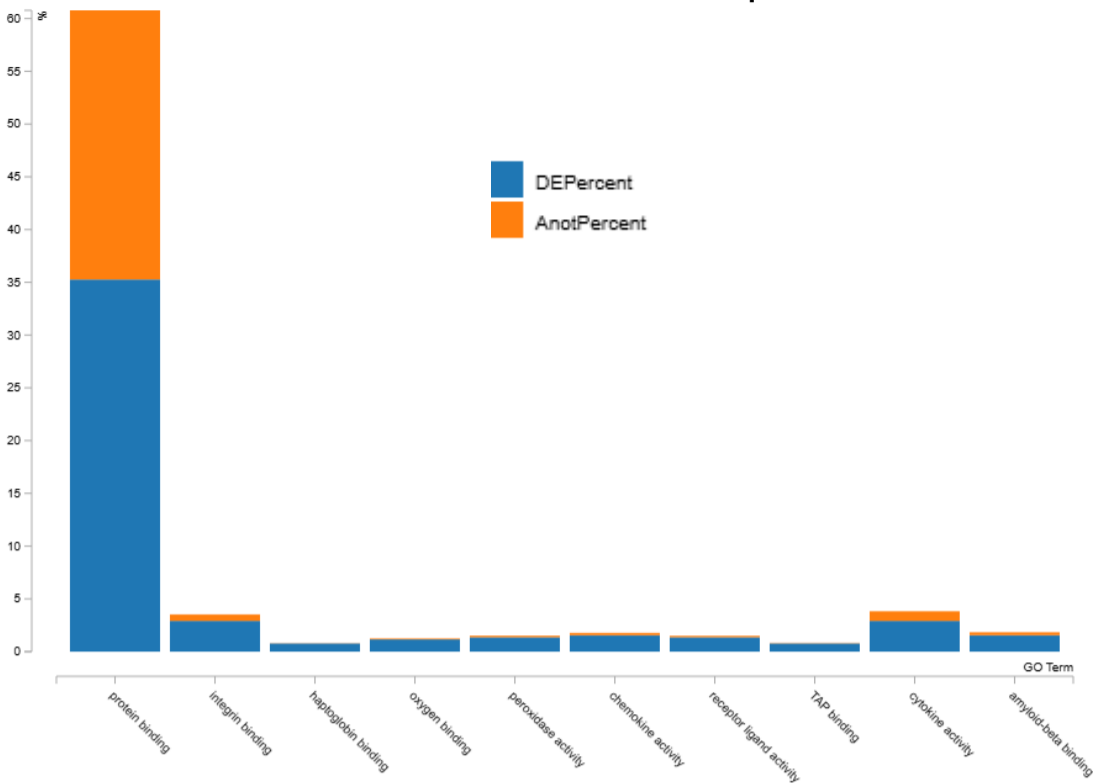

B

RNA-SeqEZPZ

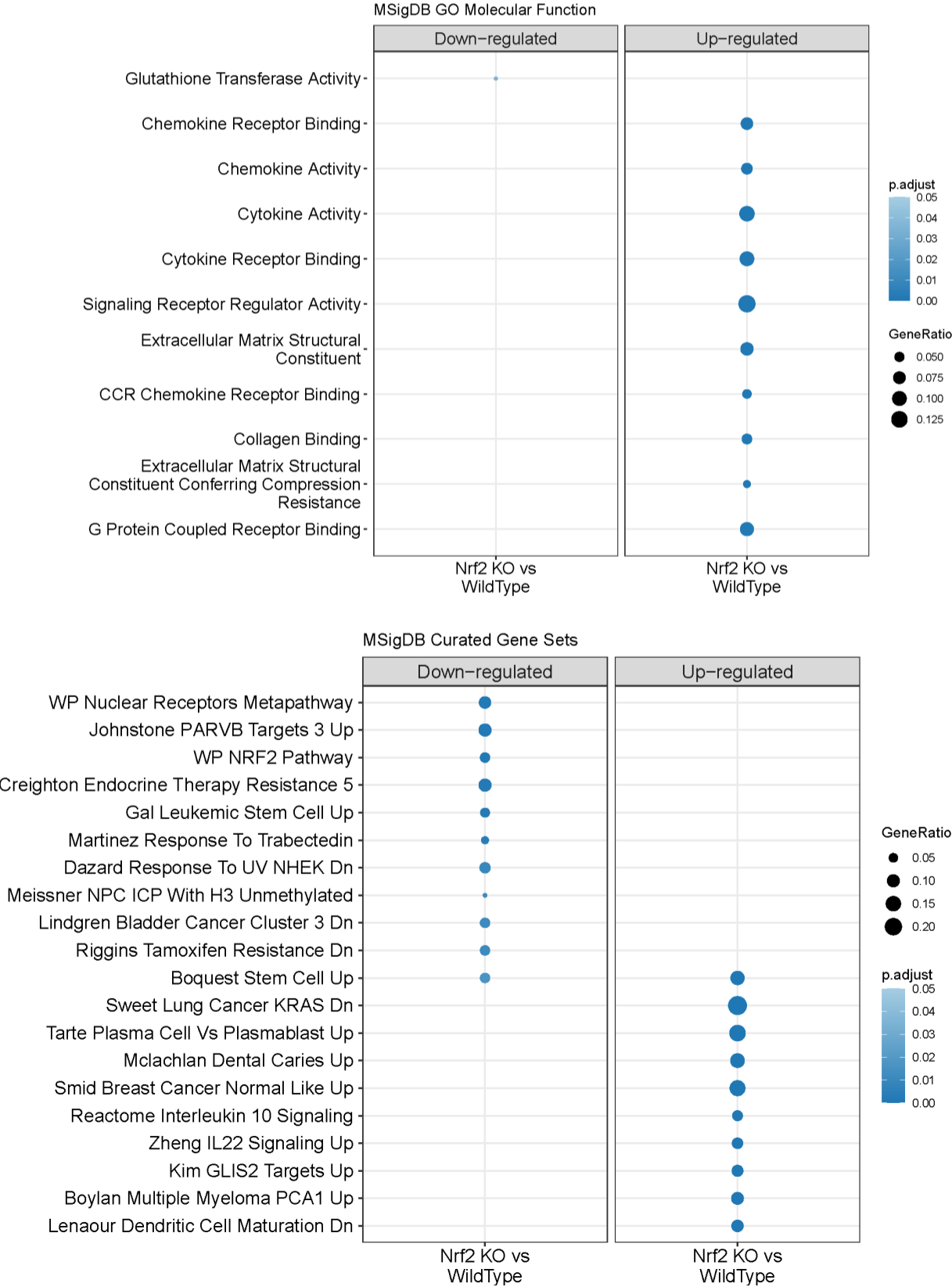

Supplement: giaf133_Supplemental_Files [file giaf133_supplemental_files.zip › Supplementary_Figure_18_pathway_Nrf2.pdf]

A

RaNA-seq

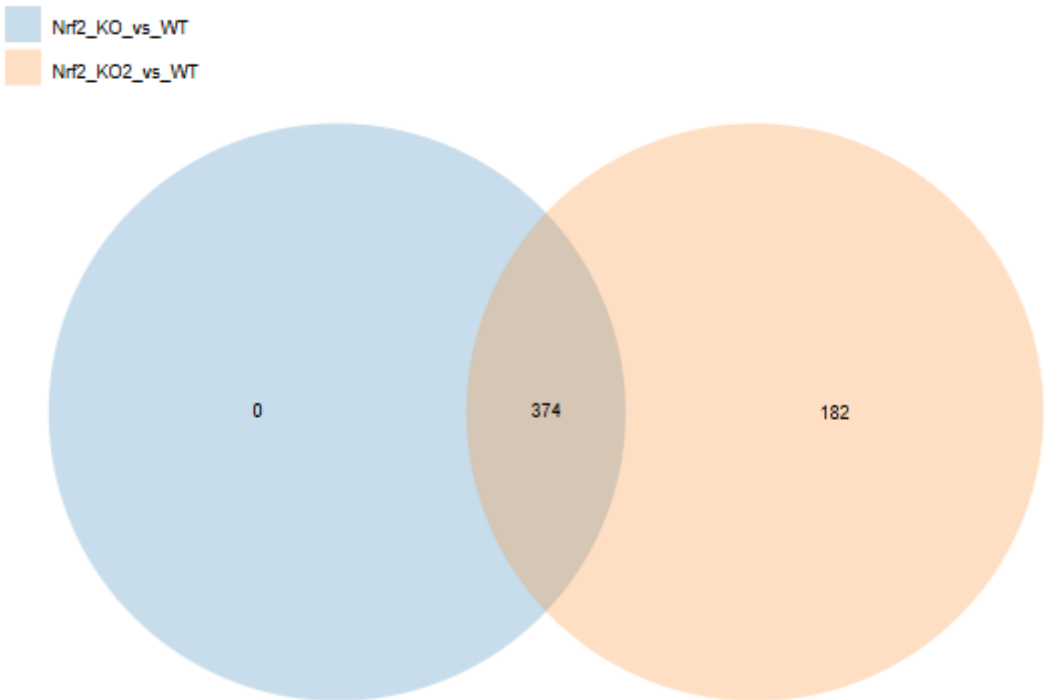

B

RNA-SeqEZIP

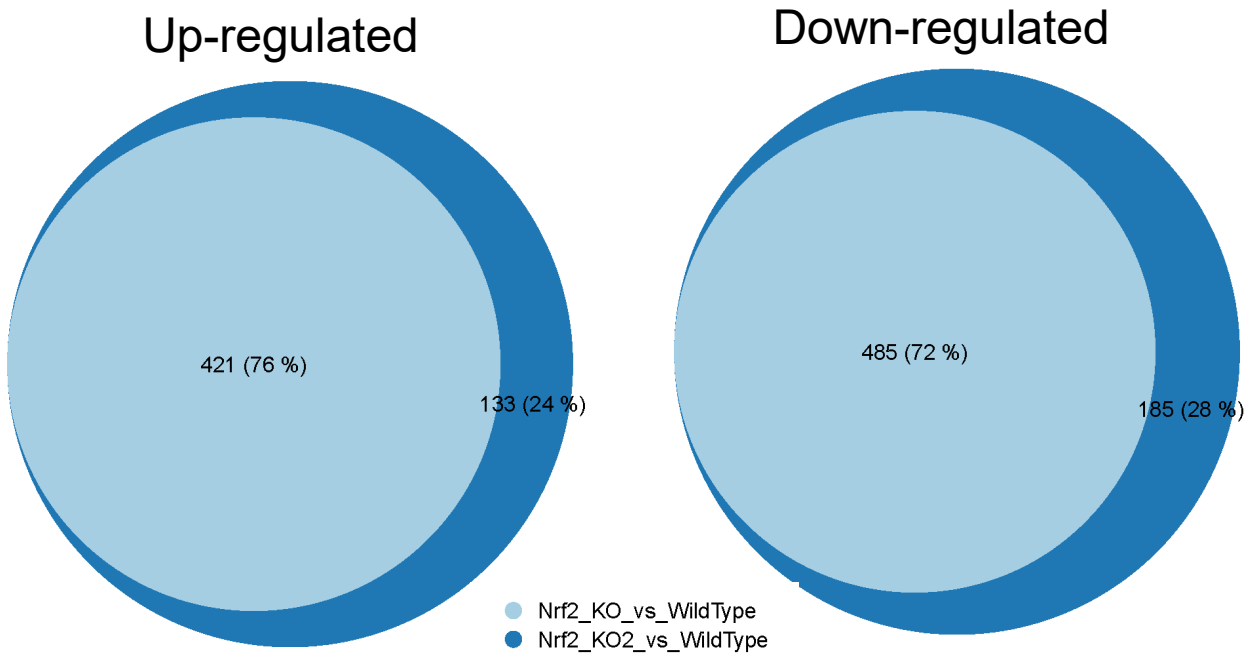

Significance of overlaps

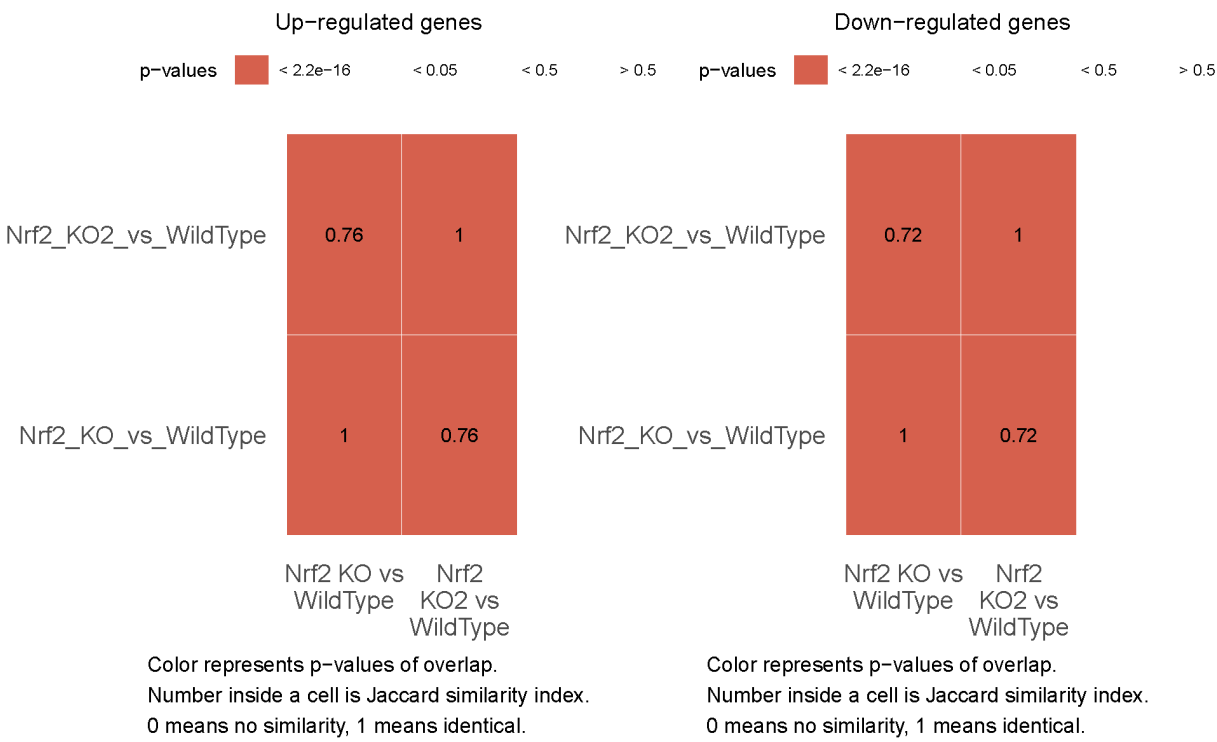

C

RNA-SeqEZIP

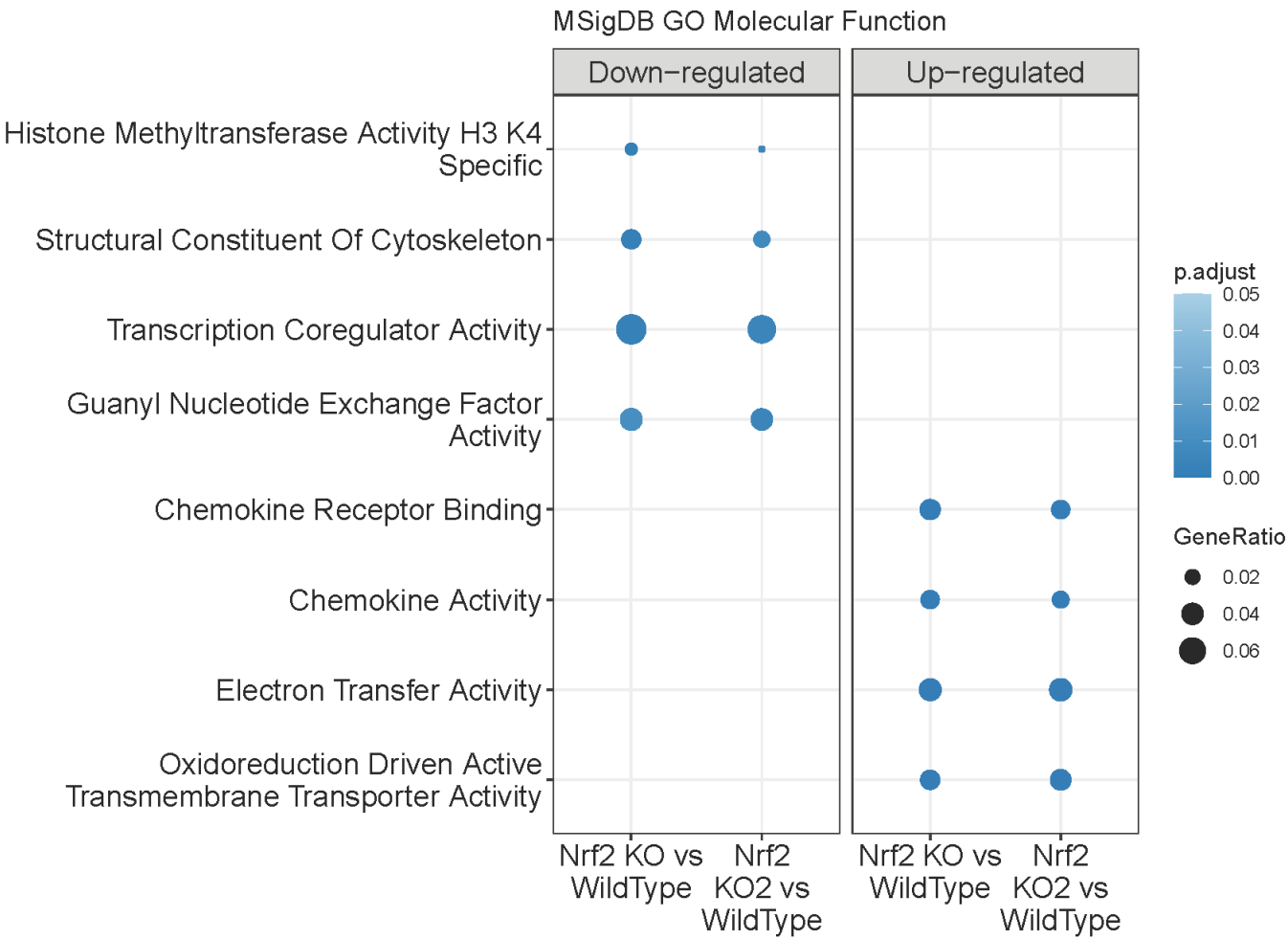

Supplement: giaf133_Supplemental_Files [file giaf133_supplemental_files.zip › Supplementary_Figure_19_comparative_RaNAseq_RNASeqEZPZ.pdf]

**A****Before adjustment**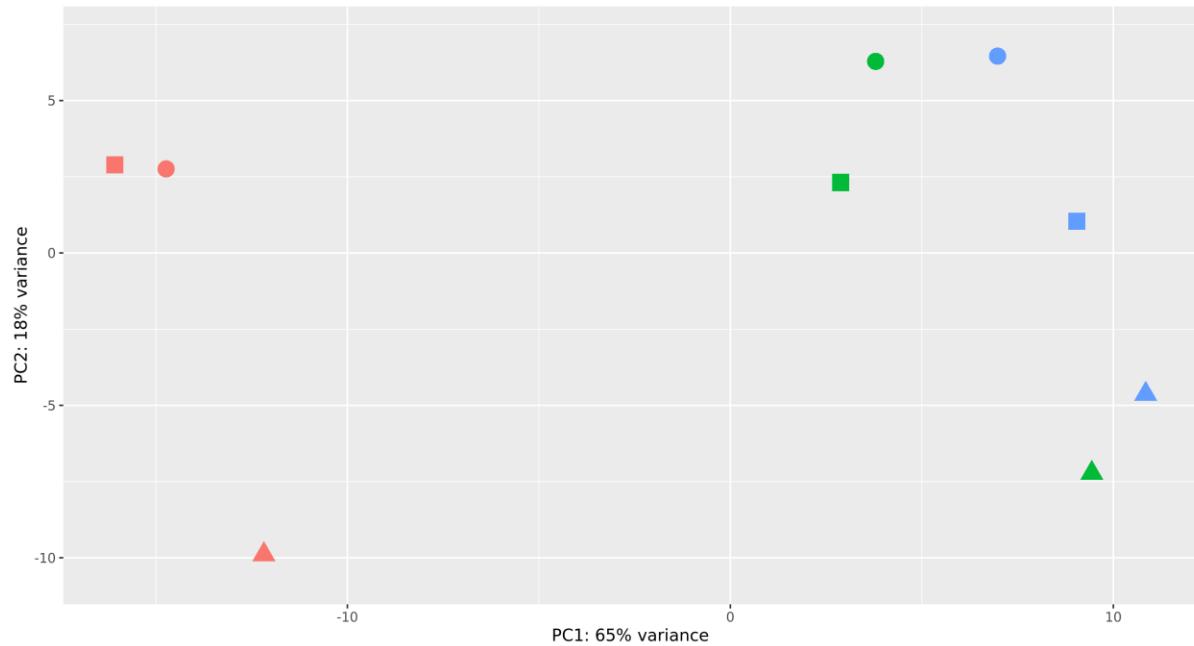**B****After adjustment**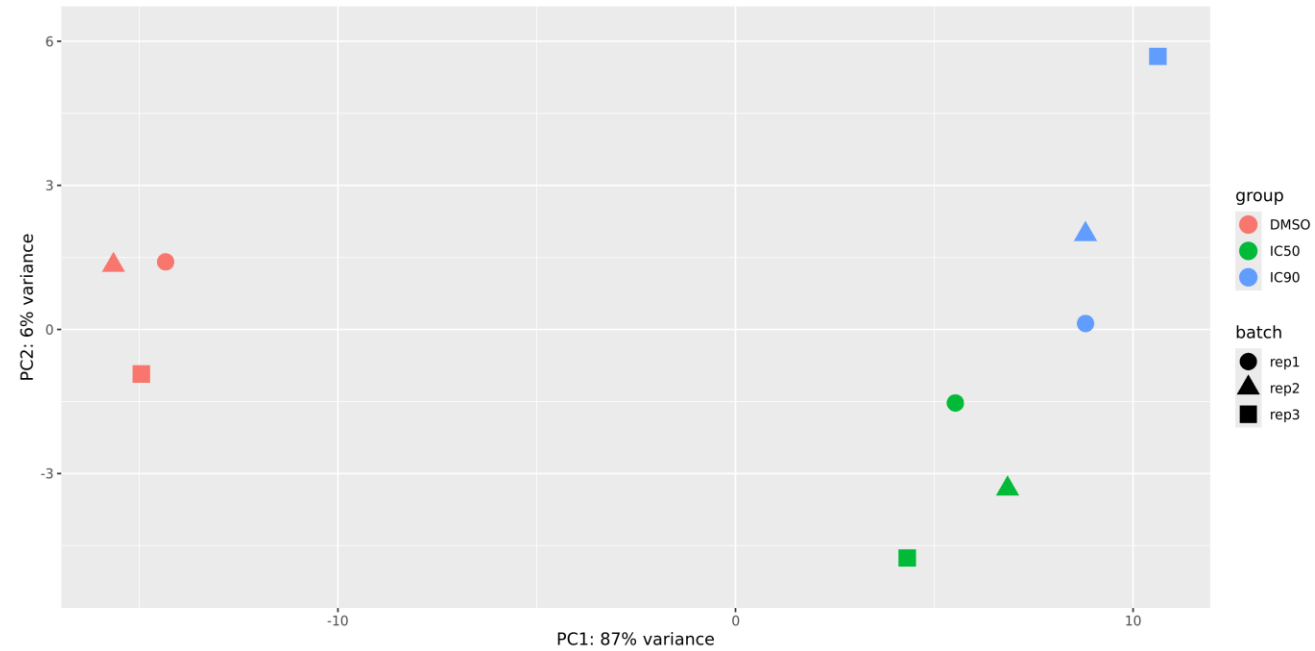

Supplement: giaf133_Supplemental_Files [file giaf133_supplemental_files.zip › Supplementary_Figure_20_PCA_seclidemstat.pdf]
