# Supplementary material for: RNA-SeqEZPZ: a point-and-click pipeline for comprehensive transcriptomics analysis with interactive visualizations
Source: Gigascience. 2025 Nov 12;15:giaf133. doi: 10.1093/gigascience/giaf133 (PMC12857227; doi:10.1093/gigascience/giaf133)
Supplement: giaf133_GIGA-D-25-00067_Original_Submission [file giaf133_giga-d-25-00067_original_submission.pdf]

GigaScience

RNA-SeqEZPZ: A Point-and-Click Pipeline for Comprehensive Transcriptomics Analysis with Interactive Visualizations

--Manuscript Draft--

**Manuscript Number:**	GIGA-D-25-00067
**Full Title:**	RNA-SeqEZPZ: A Point-and-Click Pipeline for Comprehensive Transcriptomics Analysis with Interactive Visualizations
**Article Type:**	Technical Note

Funding Information:

Funder	Recipient
American Cancer Society	Dr. Emily R. Theisen
Unravel Pediatric Cancer (RSG-22-118-01-DMC)	Dr. Emily R. Theisen
National Cancer Institute (R01 CA272872)	Dr. Genevieve C. Kendall
Alex's Lemonade Stand Foundation for Childhood Cancer (A award)	Dr. Genevieve C. Kendall
CancerFree KIDS (new idea award)	Dr. Genevieve C. Kendall
Research Institute, Nationwide Children's Hospital (startup fund)	Dr. Genevieve C. Kendall<br>Dr. Emily R. Theisen
V Foundation for Cancer Research (V scholar award)	Dr. Genevieve C. Kendall

Abstract:

Background

RNA-Seq analysis has become a routine task in numerous genomic research labs, driven by the reduced cost of bulk RNA sequencing experiments. These generate billions of reads that require easy-to-run, comprehensive, and reproducible analysis. However, many labs rely on in-house scripts, which can be challenging for bench scientist to use and hinder standardization and reproducibility. While existing RNA-Seq pipelines attempt to address these challenges, they often lack a complete end-to-end user interface.

Findings

To bridge this gap, we developed RNA-SeqEZPZ, an automated pipeline with a user-friendly point-and-click interface, enabling rigorous and reproducible RNA-Seq analysis without requiring programming or bioinformatics expertise. For advanced users, the pipeline can also be executed from the command line, allowing customization of steps to suit specific applications. The innovation of this pipeline lies in the combination of three key features: (1) all software is packaged within a Singularity container, eliminating installation issues, (2) it offers a point-and-click interface from raw FASTQ files through differential expression and pathway analysis, and (3) it includes a Nextflow version, enabling scalability and portability for seamless execution across various platforms including job submission in the cloud and cluster computing. Additionally, RNA-SeqEZPZ generates a thorough statistical report and offers an option for batch adjustment to minimize effects of noise due to technical variations across replicates. Reports can also be reviewed by a bioinformatician to ensure the overall quality of the analysis.

Conclusions

RNA-SeqEZPZ is a robust, accessible, and scalable solution for comprehensive RNA-Seq analysis, enabling researchers to focus on biological insights rather than computational challenges.

Corresponding Author:

Cenny Taslim, Ph.D.
Abigail Wexner Research Institute at Nationwide Children's Hospital
Columbus, OH UNITED STATES

Powered by Editorial Manager® and ProduXion Manager® from Aries Systems Corporation

Corresponding Author Secondary Information:	
Corresponding Author's Institution:	Abigail Wexner Research Institute at Nationwide Children's Hospital
Corresponding Author's Secondary Institution:	
First Author:	Cenny Taslim, Ph.D.
First Author Secondary Information:	
Order of Authors:	Cenny Taslim, Ph.D.
	Yuan Zhang
	Genevieve C. Kendall, Ph.D.
	Emily R. Theisen, Ph.D.
Order of Authors Secondary Information:	
Additional Information:	
Question	Response
Are you submitting this manuscript to a special series or article collection?	No
**Experimental design and statistics**<br><br>Full details of the experimental design and statistical methods used should be given in the Methods section, as detailed in our Minimum Standards Reporting Checklist. Information essential to interpreting the data presented should be made available in the figure legends.<br><br>Have you included all the information requested in your manuscript?	Yes
**Resources**<br><br>A description of all resources used, including antibodies, cell lines, animals and software tools, with enough information to allow them to be uniquely identified, should be included in the Methods section. Authors are strongly encouraged to cite Research Resource Identifiers (RRIDs) for antibodies, model organisms and tools, where possible.<br><br>Have you included the information requested as detailed in our Minimum Standards Reporting Checklist?	Yes

Powered by Editorial Manager® and ProduXion Manager® from Aries Systems Corporation

**Availability of data and materials**<br><br>All datasets and code on which the conclusions of the paper rely must be either included in your submission or deposited in publicly available repositories (where available and ethically appropriate), referencing such data using a unique identifier in the references and in the "Availability of Data and Materials" section of your manuscript.<br><br>Have you have met the above requirement as detailed in our Minimum Standards Reporting Checklist?	Yes
GigaScience has policies and guidelines in place for the use of generative AI-writing tools such as ChatGPT. If you have used such writing tools to assist with writing the manuscript this must be declared and cited in the text. Authors should not list AI-writing tools and other AI-assisted technologies as an author or co-author and should acknowledge that they are fully responsible for text generated or refined by AI-writing tools.<p><br><br>A summary of use (particularly in the introduction or among methods) needs to be included at the end of the paper, and the outputs should also be included as a supplementary file hosted in GigaDB or other open repositories. Please read our guidelines for more information. <p><br><br>By submitting to GigaScience, you are aware of the journal's AI-writing tools policy, and if you have declared use of such tools below, you have acknowledged this where appropriate in your manuscript and have made a summary of use and outputs available. <p><br><b>AI-assisted writing tools have been used in the preparation of this manuscript?	Yes

Manuscript
Click here to access/download;Manuscript;RNAseq pipeline paper_final_10.docx

RNA-SeqEZPZ: A Point-and-Click Pipeline for Comprehensive Transcriptomics Analysis with Interactive Visualizations

Cenny Taslim[1]*, Yuan Zhang[2]*, Genevieve C. Kendall[1,3†], Emily R. Theisen[1,3†]

[1]Center for Childhood Cancer Research, The Abigail Wexner Research Institute, Nationwide Children's Hospital, Columbus, OH 43215, USA.

[2]High Performance Computing Center, The Abigail Wexner Research Institute, Nationwide Children's Hospital, Columbus, OH 43215, USA.

[3]Department of Pediatrics, The Ohio State University College of Medicine, Columbus, OH 43210, USA.

*, † authors contributed equally to this work

† Corresponding authors: Genevieve C. Kendall (Genevieve.Kendall@NationwideChildrens.org) and Emily R. Theisen (Emily.Theisen@NationwideChildrens.org)

Abstract

Background

RNA-Seq analysis has become a routine task in numerous genomic research labs, driven by the reduced cost of bulk RNA sequencing experiments. These generate billions of reads that require easy-to-run, comprehensive, and reproducible analysis. However, many labs rely on in-house scripts, which can be challenging for bench scientist to use and hinder standardization and reproducibility. While existing

RNA-Seq pipelines attempt to address these challenges, they often lack a complete end-to-end user interface.

Findings

To bridge this gap, we developed RNA-SeqEZPZ, an automated pipeline with a user-friendly point-and-click interface, enabling rigorous and reproducible RNA-Seq analysis without requiring programming or bioinformatics expertise. For advanced users, the pipeline can also be executed from the command line, allowing customization of steps to suit specific applications. The innovation of this pipeline lies in the combination of three key features: (1) all software is packaged within a Singularity container, eliminating installation issues, (2) it offers a point-and-click interface from raw FASTQ files through differential expression and pathway analysis, and (3) it includes a Nextflow version, enabling scalability and portability for seamless execution across various platforms including job submission in the cloud and cluster computing. Additionally, RNA-SeqEZPZ generates a thorough statistical report and offers an option for batch adjustment to minimize effects of noise due to technical variations across replicates. Reports can also be reviewed by a bioinformatician to ensure the overall quality of the analysis.

Conclusions

RNA-SeqEZPZ is a robust, accessible, and scalable solution for comprehensive RNA-Seq analysis, enabling researchers to focus on biological insights rather than computational challenges.

Introduction

Data analysis of RNA-Seq consists of a set of successive stages that are repetitive and routinely executed using a wide variety of tools. Typically, analysis starts with quality control of raw sequence reads or FASTQ files, and is followed by alignment of reads to a reference genome, filtering of low-quality reads,

counting reads that aligned to a specific feature/gene, differential analysis of genes in different condition and finally visualization of the results[1]. In house analysis usually involves a bioinformatician creating step-by-step scripts for specific datasets which will need to be modified for different datasets. With each modification and customization, it is notoriously challenging to keep the analysis fully reproducible primarily due to differences in scripts, hardware, operating systems, and software versions. Reproducibility is critical for a rigorous analysis to ensure reliable validation of scientific findings and has long been a challenging issue in biomedical research[2]. A recent publication found that a large majority of existing Jupyter notebooks (a popular format for documenting and sharing computational workflow) could not be executed automatically and failed to reproduced the results[3]. Reproducibility issues have even led to a retraction of an epidemiological paper[4].

Furthermore, wet lab scientists who conduct the RNA-Seq experiments and generate libraries often have limited programming and bioinformatics experience, making it challenging for them to analyze their own data efficiently while ensure statistical rigor and reproducibility. This creates a strong demand for an easy-to-use, comprehensive pipeline that expedites routine RNA-Seq analysis without sacrificing the quality and reproducibility of the results. Here, we describe RNA-SeqEZPZ, a point-and-click tool for comprehensive analysis of RNA-Seq experiments from FASTQ to result visualization. RNA-SeqEZPZ is primarily designed to empower bench scientists to do their own analyses and explore their results while also providing bioinformaticians with the flexibility for further customization.

There are several existing RNA-Seq pipelines, with ENCODE[5] and nf-core[6] being among the most widely used. In comparison to these pipelines, a notable feature of RNA-SeqEZPZ is its point-and-click interface starting from FASTQ files up to differential genes analysis and interactive visualization capabilities. ENCODE[5] does not perform differential genes analysis and have no interactive visualization. The nf-core[6] RNA-Seq pipeline does offer interactive visualization and differential genes analysis. However, it is a separate visualization module that must be run independently using command line after the completion

of the RNA-Seq pipeline. Several shiny apps providing a graphical interface for RNA-Seq analysis such as ROGUE[7], Shiny-Seq[8] and bulkAnalyseR[9] have also been previously published. However, none of these tools support analyzing RNA-Seq experiments starting from raw FASTQ files. Furthermore, at the time of writing, Shiny-Seq[8] appears to be no longer accessible, as its official website (https://schultzelab.shinyapps.io/Shiny-Seq/) redirect to a "not found" page on FastGenomics. Access to ROGUE (https://marisshiny.research.chop.edu/ROGUE/) was repeatedly interrupted by server issues, which may impact its usability for analysis. We found that Partek™ flow and RaNA-Seq[10] offers functionalities most similar to RNA-SeqEZPZ. However, they require uploading FASTQ files to their server which can be complicated by connection and firewall issues. Furthermore, no source code is provided, making customization impossible. RASflow[11] offers analysis starting from FASTQ files but it doesn't provide a user interface to select FASTQ files. The comparison of these tools with RNA-SeqEZPZ is presented in Supplementary Table S1.

To the best of our knowledge, RNA-SeqEZPZ is the first open-source tool to offer a point-and-click interface with interactive plots, starting from raw FASTQ reads and providing analytical capabilities from differential genes analysis to pathway analysis. This pipeline can potentially accelerate research progress by simplifying a complex process, enhancing reproducibility within and across labs, and empowering researchers with the tools to interpret their own results. With the extensive reports generated by the pipeline, a bioinformatician can supervise the entire process by reviewing the reports to ensure accuracy and proper execution.

Methods

RNA-SeqEZPZ can be started using a single command after downloading a Singularity image and cloning the Git repository (Figure 1 and Supplementary Figure 1). It encompasses multiple steps, utilizes various

tools, and generates statistical reports, visualization, and diverse output files. The pipeline accepts gzipped paired-end FASTQ files as input and supports analysis for 20 genomes including human, zebrafish, and mouse. Users can select all the inputs through a point-and-click interface implemented using a Shiny app[12] and shinyFiles[13] allowing them to initiate a comprehensive analysis effortlessly.

Figure 1. Overview of RNA-SeqEZPZ workflow, interface, and analysis outputs. Installation begins with pulling a Singularity image and cloning a git repository. After installation, the software can be run with a single command, which launches a web interface allowing users to select parameters and FASTQ files. Once the “Run full analysis” is clicked, multiple processes were run. An assortment of statistical outputs was created, and interactive visual interfaces are provided.

Software Implementation

RNA-SeqEZPZ is a combination of a Shiny[1] app with either bash scripts and SLURM[14] (a cluster resource management system) or Nextflow[15], a workflow management systems (Figure 1). The Shiny app at the front end provides an interface for users to run the entire analysis. As SLURM[14] is the most widely used workload manager in HPC[16], using it in bash scripts will enable users to easily modify the scripts as needed and leverage their existing familiarity with the system. Nextflow[15] is a modern workflow management system designed to simplify the development and deployment of complex data analysis pipelines. Nextflow[15] enhances the flexibility of this pipeline to run on diverse computation infrastructures with workload managers other than SLURM[14]. The required R packages, and all other tools needed for analysis including Firefox (the browser used for the interface) are enclosed inside a Singularity[17] container removing any potential difficulties involved in the installation of all the required software. Altogether this promotes the reproducibility, standardization, and portability of the RNA-SeqEZPZ pipeline. Further, because the shiny app and analysis can be run locally on a cluster, there is no need to transfer gigabytes to terabytes of data to an external server over the internet.

Installation and Usage

Installation instructions are provided in detail at https://github.com/cxtaslim/RNA-SeqEZPZ. Briefly, a Singularity image is pulled from a repository. Then, depending on user preference, either a Nextflow or a bash/SLURM repository containing all the necessary scripts should be cloned (Figure 1). To use the pipeline, users need to connect to their HPC cluster and run a one-line command: "`bash run_shiny_analysis.sh`" which will bring up a Firefox browser where user will be able to select the sample FASTQ file path, output path, resource requirements, and various settings. Options are also available for running the steps of the pipeline individually (see the manual on the website for details). To assist users running this for the first time, we have provided example datasets that can be downloaded from https://github.com/cxtaslim/RNA-SeqEZPZ, along with an easy-to-follow step-by-step tutorial.

Workflow Overview

RNA-SeqEZPZ performs multiple steps. The process begins with merging FASTQ files from different sequencing lanes using cat command in Bash. Raw reads quality control are then performed using FASTQC[18] and QC reports are compiled using MultiQC[19]. Low-quality and adapter sequences are trimmed using trim_galore[20]. Following quality control and trimming, read alignment is conducted using STAR[21] two-pass approach, which enhances mapping accuracy. BigWig tracks are generated using bamCoverage[22] and WiggleTools[23] for visualization. Gene-level read quantification is carried out using featureCounts[24]. For differential expression analysis, DESeq2[25] with batch adjustment are used, and statistical reports are generated by SARTools[26]. Batch effects are accounted for by incorporating replicates as a covariate in the model. In the PCA plot generated by the pipeline, the effect of batch adjustment is estimated using limma[27].

Interactive Visualization

To provide additional insights into gene expression analysis, RNA-SeqEZPZ includes several interactive visualization tools. Volcano plots are generated using ggplot2[28] to highlight differentially expressed genes. Area-proportional Euler and Venn diagrams, along with UpSet plots, are generated using Eulerr[29], venn[30] and UpSetR[31] to visualize gene overlaps. The significance of overlap is assessed by testing the independence of two variables using Fisher's exact test[32]. Additionally, the Jaccard Index[33] which quantifies the similarity between gene lists, is computed using GeneOverlap[34] package. For pathway analysis, over-representation analysis is conducted using clusterProfiler[35], utilizing gene sets annotations from MSigDB via the msigdbr[36] package. These interactive tools provide deeper insights into gene expression functions and biological significance.

Rationale for tool selection

RNA-SeqEZPZ is designed as an easy-to-use and accessible pipeline for researchers with no prior experience in RNA-Seq analysis. To ensure simplicity, a single tool is selected for each step based on best practices and recommendations from the Hitchhiker's Guide[1]. For advanced users, the code is fully accessible, allowing customization, tool substitution, and modifications as needed.

For read alignment, STAR[21] was chosen due to its high performance RNA-Seq mapping capabilities[37]. The alignment process occurs in two stages: first, initial mapping identifies potential novel splice junctions, followed by a refined alignment using both known annotations and the newly detected junctions. These two steps approach enhances read mapping accuracy and improves sensitivity.

In our pipeline, we focus on quantifying reads at the gene level, as all isoforms of the same gene typically share the same pathway annotations. To achieve this, we selected featureCounts[24], a fast and efficient quantification of mapped RNA-Seq based on genome alignment. Additionally, a comparative evaluation of seven widely used quantification algorithms demonstrated that featureCounts[24] has higher

sensitivity in detecting single-isoform genes while delivering comparable performance on real datasets[38,39].

For differential expression analysis, DESeq2 was selected based on findings by Rapaport et al. (2013)[40], which demonstrated its superior specificity and sensitivity as well as good control of false positive errors. Additionally, DESeq2 accounts for batch effects by incorporating batch variables into its Generalized Linear Model (GLM) design formula as covariates removing unwanted technical variation.

Reproducibility

Reproducibility has long been a key issue in bioinformatics analysis[4,41]. Ensuring the ability to execute an existing workflow and reproduce the same exact results is crucial for advancing scientific research[2]. To achieve this goal, we employed several solutions following best practices[42–44] to ensure RNA-SeqEZPZ is highly reproducible.

Software Containerization

To prevent dependency mismatches and ensures consistency across computational environments, we encapsulated all software dependencies within a Singularity[17] container. This guarantees that RNA-SeqEZPZ can be used across multiple environments including local machines, cloud platforms, or high performance computing (HPC) cluster eliminating issues caused by dependency mismatches. Unlike Docker[45], another popular containerization platform that requires root privileges, Singularity[17] operates without the need for elevated permission, making it ideal in shared environment such as HPC clusters. Additionally, using Singularity[17] eliminates manual installation of software on different systems and ensures it yields the same results on a different machine.

Workflow Documentation

Beyond software dependencies, Kim et al.[44] emphasize the importance of comprehensive documentation and readable code for ensuring reproducibility. Documenting analysis steps and software can be challenging, as bioinformatics workflows often consists of a multitude of tools and steps which are chained together to create complex analysis workflow. Additionally, minimizing manual steps that are required to execute an analysis workflow is crucial, which is why computational pipelines is needed to automate the integration and execution of these tools.

RNA-SeqEZPZ implemented as a Bash-based pipeline, is designed for readability and ease of use. It automates workflow execution, supports the re-analysis of failed runs, and generates comprehensive documentation on data processing ensuring transparency, code sharing and long-term reproducibility. However, tasks such as re-analysis of failed runs and documentation must be implemented manually. To further enhance flexibility, automation, and resource management, RNA-SeqEZPZ leverages Nextflow[15], a powerful bioinformatics workflow manager. Workflow managers, including Nextflow have been recognized as key solutions of achieving reproducibility[43]. Beyond ensuring reproducibility, Nextflow enables easy parallelization, job-scheduling, re-analysis of failed runs, seamless integration of software containerization and efficient resource management. Additionally, it automates generation of execution report with detailed information, such as input parameters to the pipeline, software versions, and resource usage information, further optimizing workflow efficiency and reproducibility[43].

Code Sharing and Accessibility

To promote transparency and reproducibility, we ensure that all code is publicly accessible via an online repository such as GitHub. This allows other researchers to review, modify, and extend RNA-SeqEZPZ, fostering collaboration and long-term sustainability. The integration of these solutions collectively ensures that RNA-SeqEZPZ maintains a high level of reproducibility.

Below, we describe in more details the components of the RNA-SeqEZPZ interface, including interactive plots implemented using Shiny[12].

User Friendly Interface and Generated Outputs

A primary design goal of RNA-SeqEZPZ is to accelerate full analysis of RNA-seq datasets and provide interactive analysis of the results. As such, the pipeline is designed to be run with a one-line command in the terminal that loads a user-friendly interface implemented as a Shiny[12] app (Figure 1).

The interface accessed through a Firefox browser, allowing users to easily zoom in or out, enlarge text, and adjust the window size for better visibility. To run the analysis, users simply select their FASTQ files and provide the necessary information through an intuitive file browser interface (Supplementary Figure 2). After entering all sample information, clicking "Run full analysis" will automatically execute the full analysis as described above (Figure 1).

During the analysis, users can monitor progress through the "Log" tab (Supplementary Figure 3). Upon completion, the run_rnaseq_full.out log file will display the message "Done running RNA-seq full analysis". The files in the "Log" tab display the current step being processed by the pipeline. Once the analysis is completed, users will be able to click on the "QC" tab and see all the quality control metrics compiled by MultiQC[19] (Supplementary Figure 4). The MultiQC[19] generated HTML files are interactive as well, which allows for some customization of the plots (Supplementary File 1). The QC report includes metrics for raw reads, alignment rate, number of duplicated reads, percentage of reads aligned to genomic features, etc. A statistical report of the differential gene analysis can be viewed in the "Outputs" tab (Supplementary Figure 5). This report is generated using a modified version of SARTools[26]. The report contains description of raw data, Principal Component Analysis (PCA) plot and hierarchical clustering of samples to explore the variability within and between samples. The statistical report also

described the steps performed in the differential analysis using DESeq2[25] along with the statistical assumptions and validation of the choices used (Supplementary File 2).

Under "Plots" tab users can adjust the cut-offs for significant differential genes and in table, they can find the $\log_2$ fold-change of their gene of interest (Supplementary Figure 6), create volcano and UpSet plots (Supplementary Figure 7 and Supplementary Figure 8), perform overlap (Supplementary Figure 9) and pathway analysis (Supplementary Figure 10). The GeneOverlap[34] package was utilized to compute the Jaccard similarity index[33] and Fisher's exact test[32] to evaluate the significance of overlap between the gene lists (Figure 2C). The overlaps between genes in different conditions were visualized using proportional Euler and Venn diagrams, as well as an UpSet plot, created using eulerr[46], Venn[30] and UpSetR[47] packages. Pathway analysis or Over-Representation analysis was conducted using clusterProfiler[35] and msigdbr[36] packages. All other plots were generated using ggplot2[48] package.

Additionally, since the files, including intermediate ones generated by the pipeline can accumulate to terabytes in size, we provide users a simple way to delete projects and files they no longer require (Supplementary Figure 11). To assist in this process, we provide explanations to help users determine whether to keep or delete these files.

Furthermore, in our Nextflow version, we provide an interface to view the report generated by Nextflow (Supplementary Figure 12 and Supplementary File 6).

Public dataset analysis

In order to show the utility of the pipeline, we re-analyzed the RNA-Seq experiments in the study of novel Ewing Sarcoma fusion proteins[49]. RNA-SeqEZPZ was run on two biological replicates from a knockdown/rescue experiment in the A673 human cell line where the endogenous fusion oncogenic transcription factor EWSR1::FLI1 was depleted by shRNA and then rescued with either EWSR1::FLI1 or

EWSR1::ETV4 constructs. These samples were compared to control cells with no rescue (KD). The FASTQ files can be downloaded from GEO (GSE173185).

As shown in the QC report, for EWSR1::ETV4 rescued sample replicate 1, there are 48.5 million aligned reads (83.4% alignment rate) and 53.7% of these reads are assigned to a feature (Supplementary File 1). The PCA plot in the statistical report shows that the 6 samples cluster first by replicates and then by rescue condition. This suggests that experimental conditions significantly influence on the observed variability and that the samples within each replicate group are highly similar, indicating good reproducibility (Figure 2). Differential genes were identified with FDR < 0.05. In samples where EWSR1::FLI1 was rescued, it correctly shows FLI1 as up-regulated and in samples where EWSR1::FLI1 were knockdown and then rescued with EWSR1::ETV4 construct it shows FLI1 as down-regulated and ETV4 as up-regulated genes compared to knockdown control (Figure 2). Well known targets of EWSR1::FLI1 such as LOX1 and CAV1[50,51] are shown as down- and up-regulated in both EWSR1::FLI1 and EWSR1::ETV4 rescued samples. There is significant overlap between genes up-regulated (3,104 genes, p-values < 0.05) and genes down-regulated (2,830 p-value < 0.05) by both EWSR1::FLI1 and EWSR1::ETV4 suggesting that EWSR1::ETV4 regulates similar genes as EWSR1::FLI1. Consistent with overlap analysis that shows significant overlap between genes, pathway analysis indicates that genes regulated by EWSR1::ETV4 and EWSR1::FLI1 are involved in many similar functions (Figure 2 and Supplementary File 3). EWSR1::FLI1 downregulated genes are consistent with those identified in a previous study by Kinsey *et al.*[52] (Supplementary File 3). The QC report (Supplementary File 1) and statistical report of the differential analysis (Supplementary File 2) are saved as HTML files. All the plots created in RNA-SeqEZPZ can be exported as a pdf file (Supplementary File 3). One of the widely used outputs for downstream analysis is the list of differentially expressed genes. These tables list genes that are defined as significant along with their Ensembl ID, raw and normalized read count, fold-changes, p-values adjusted for

multiple testing, and other statistics generated by the DESeq2 models (Supplementary File 4). Video tutorial on the analysis of this dataset is included in Supplementary File 5.

Figure 2. Analysis results of samples rescued with EWSR1::FLI1 and EWSR1::ETV4 constructs. (A) PCA plot showing good separation between the two different conditions. (B) Volcano plots for the two rescue construct showing highlighted known targets of EWSR1::FLI1 in addition to FLI1 and ETV4 indicative of the rescue conditions. (C) Overlap analysis reveals a significant overlap between genes regulated by the two constructs. Box colors indicate p-values of overlaps, while the number inside the boxes represent the Jaccard Similarity Index.

Effects of Batch Adjustment

In order to highlight the benefits of adjusting for batch effects, we re-analyze RNA-Seq experiments from "The DBD-α4 helix of EWSR1::FLI1 is required for GGAA microsatellite binding that underlies genome regulation in Ewing sarcoma"[53]. The FASTQ files were obtained from GEO (GSE268944). RNA-SeqEZPZ was used to analyze two biological replicates of knockdown/rescue experiment in the TTC-466, an Ewing Sarcoma cell line. The two samples analyzed are DBD+, where a mutant construct of EWSR1::FLI1 was used to rescue expression, and samples in which a full-length EWSR1::FLI1 construct was used for rescue. Figure 3A presents the PCA plot before batch adjustment. Based on the plot, it is difficult to definitively determine whether the biological replicates cluster together, as DBD+ replicate 2 appears to be closer to EWSR1::FLI1 replicate 3. However, after adjusting for batch effect, the DBD+ replicates cluster together, separating from EWSR1::FLI1 samples along PC1, which accounts for 83% of the variance (Figure 3B). Furthermore, batch adjustment increased the variance explained by PC1 from 78% to 83%, further clarifying sample separation.

Figure 3. PCA plot demonstrating the impact of batch adjustment. (A) PCA plot before batch adjustment and (B) PCA plot after batch adjustment, showing the improved separation achieve through batch correction.

All figures for the analyses of public datasets were generated using RNA-SeqEZPZ and modified using Adobe Illustrator™. ChatGPT[54] was used to assist in drafting the manuscript.

Discussions

In summary, RNA-SeqEZPZ provides an easy point-and-click comprehensive analysis of RNA-Seq data which enables biologists to analyze and explore the nuances of their own experiments. The implementation of RNA-SeqEZPZ ensures reproducible analysis and is broadly flexible for running in various computational infrastructures. RNA-SeqEZPZ also provides an entry point analysis for more advanced users where they can download the results and do additional downstream analysis or modify the pipeline to include more features. Thus, RNA-SeqEZPZ represents a valuable easy-to-use tool for the scientific community, enabling the analysis, interpretation, and discovery of insights about gene function and regulation through RNA-Seq experiments. With its interface integrated with Singularity container combined with computational pipeline and workflow management systems, the code provides a flexible framework that can be easily expanded to support additional interactive visualizations and more advanced analyses such as single cell RNA-Seq, spatial RNA-Seq and multiomics.

Key points

- RNA-SeqEZPZ is a user-friendly pipeline with point-and-click interface starting from raw FASTQ files for comprehensive RNA-Seq analysis, enabling both novice and experienced users to perform complex analyses with ease.

- RNA-SeqEZPZ enables researchers to analyze and compare differential gene expressions across varying experimental conditions, with intuitive visualization tools for exploring and interpreting results.
- RNA-SeqEZPZ provides a containerized image and uses bioinformatics systems managers, ensuring straightforward installation, seamless deployment across environments, and reproducibility of the analyses performed.
- RNA-SeqEZPZ is freely available and can be downloaded from https://github.com/cxtaslim/RNA-SeqEZPZ and https://github.com/yzhang18/RNA-SeqEZPZ-NF (Nextflow version).

Additional files

Supplementary Figures

Supplementary Figure 1: RNA-SeqEZPZ workflow showing output files generated. Some icons were sourced and/or adapted from https://nf-co.re/dualrnaseq, created by Regan Hayward under the MIT license.

Supplementary Figure 2: A screenshot of the run analysis interface where users will be able to click-and-select their FASTQ files, reference genome, and other inputs. There is an “i” icon which will provide more information when hovered over in the interface.

Supplementary Figure 3: A snapshot of the log file providing information on the current progress of the RNA-Seq analysis.

Supplementary Figure 4: A screenshot of the quality control report in interactive HTML format that can be viewed by users by clicking the “QCs” tab.

Supplementary Figure 5: A screenshot of the interface in RNA-SeqEZPZ to view and interact with statistical report generated automatically. The left navigation bar makes it easy to move to different section of the HTML file.

Supplementary Figure 6: A screenshot of the table interface showing the sorted log2 Fold-Change, False Discovery Rate (FDR) and read counts difference between treatment and control samples. Users can type in gene names to find their expressions.

Supplementary Figure 7: A screenshot of the interface where users can highlight specific genes in the volcano plot and change their significance cut-offs.

Supplementary Figure 8: A screenshot of the interface to create UpSet plot.

Supplementary Figure 9: A screenshot of the interface to perform overlap analysis which include generation of area-proportional Euler diagram, Venn diagram up to seven groups overlaps and heatmap showing the p-values of overlap and the Jaccard similarity index.

Supplementary Figure 10: A screenshot of the interface to perform pathway analysis with gene sets from MsigDB database. Enrichments are calculated for molecular function, biological processes, cellular components gene ontologies, curated and Hallmark gene sets (not shown).

Supplementary Figure 11: The clean-up interface to assist users with removing big files such as aligned and merged FASTQ files.

Supplementary Figure 12: The interface to view the report generated by Nextflow.

Supplementary Files

Supplementary File 1: QC report for the knockdown /rescue of EWSR1::FLI1 (iEF_EF) and EWSR1::ETV4 (iEF_EE4) in the A673 cell line. Link to Supplementary Files

Supplementary File 2: Statistical report for the knockdown/rescue of EWSR1::FLI1 (iEF_EF) and EWSR1::ETV4 (iEF_EE4) in the A673 cell line. PCA plot is shown in Fig.2A. Link to Supplementary Files

Supplementary File 3: Plots generated from the interface after the analysis of EWSR1::FLI1 and EWSR1::ETV4 knockdown/rescue in the A673 cell line. Selected plots are shown in Fig.2B-D. Link to Supplementary Files

Supplementary File 4: A list of up-regulated genes from the comparison of EWSR1::FLI1 knockdown/rescue to the empty construct. Link to Supplementary Files

Supplementary File 5 Report generated by RNA-SeqEZPZ-NF for analysis of EWSR1::FLI1 and EWSR1::ETV4 knockdown/rescue in the A673 cell line, containing executed commands, CPU and memory usage, providing valuable insights for efficient resource management. Link to Supplementary Files

Data Availability

No new data were generated for this study. The data used in this article are available in NCBI Gene Expression Omnibus (GEO) repository (https://www.ncbi.nlm.nih.gov/geo/), under the accession number GSE173185 for A673 and GSE268944 for TTC-466 samples. The Supplemental files 1, 5, and 6 along with the archived software and input files are stored in the GigaDB.

Acknowledgements

This research was partially supported by the High Performance Computing Facility at the Abigail Wexner Research Institute (AWRI), Nationwide Children’s Hospital. Assistance with figures provided by the AWRI Children's Graphics Resource Group and William Clarence Ray Ph.D.

Funding

E.R.T. is grateful for support from institutional startup funds, an American Cancer Society Research Scholar Grant and RSG-22-118-01-DMC, and an Unravel Pediatric Cancer grant. G.C.K. is grateful for support from an NIH/NCI R01 CA272872 grant, an Alex’s Lemonade Stand Foundation “A” Award, a V Foundation for Cancer Research V Scholar Award, a CancerFree Kids New Idea Award, and Startup Funds from The Abigail Wexner Research Institute at Nationwide Children’s Hospital. The funders had no role in study design, data collection and analysis, decision to publish, or preparation of the manuscript. Further, the content is solely the responsibility of the authors and does not necessarily represent the official views of the National Institutes of Health.

Competing Interest Statement

The authors declare no competing interests.

Author Contributions

C.T, Y.Z., G.C.K, E.R.T. conceived the main idea, the framework of the pipeline and the manuscript. C.T, Y.Z., G.C.K, E.R.T drafted and improved the manuscript. C.T and Y.Z. developed and implemented the

pipeline. G.C.K and E.R.T revised the manuscript, supervised the development of the pipeline, and provided funding. All authors read and commented on the manuscript.

References

1. Chen, J.-W. W., Shrestha, L., Green, G., Leier, A. A. & Marquez-Lago, T. T. The hitchhikers' guide to RNA sequencing and functional analysis. *Brief. Bioinform.* **24**, bbac529 (2023).

2. Baykal, P. I. *et al.* Genomic reproducibility in the bioinformatics era. *Genome Biol.* **25**, 213 (2024).

3. Samuel, S. & Mietchen, D. Computational reproducibility of Jupyter notebooks from biomedical publications. *Gigascience* **13**, 1–23 (2024).

4. Meyerowitz-Katz, G., Besançon, L., Flahault, A. & Wimmer, R. Impact of mobility reduction on COVID-19 mortality: absence of evidence might be due to methodological issues. *Sci. Rep.* **11**, (2021).

5. Hitz, B. C. *et al.* The ENCODE Uniform Analysis Pipelines. *bioRxiv* (2023) doi:10.1101/2023.04.04.535623.

6. Ewels, P. A. *et al.* The nf-core framework for community-curated bioinformatics pipelines. *Nat. Biotechnol. 2020 383* **38**, 276–278 (2020).

7. Farrel, A. *et al.* ROGUE: an R Shiny app for RNA sequencing analysis and biomarker discovery. *BMC Bioinformatics* **24**, 1–13 (2023).

8. Sundararajan, Z. *et al.* Shiny-Seq: advanced guided transcriptome analysis. *BMC Res. Notes* **12**, 432 (2019).

9. Moutsopoulos, I., Williams, E. C. & Mohorianu, I. I. bulkAnalyseR: An accessible, interactive

pipeline for analysing and sharing bulk multi-modal sequencing data. *bioRxiv* 2021.12.23.473982 (2022) doi:10.1101/2021.12.23.473982.

10. Prieto, C. & Barrios, D. RaNA-Seq: interactive RNA-Seq analysis from FASTQ files to functional analysis. *Bioinformatics* **36**, 1955–1956 (2020).

11. Zhang, X. & Jonassen, I. RASflow: An RNA-Seq analysis workflow with Snakemake. *BMC Bioinformatics* **21**, 1–9 (2020).

12. Chang, W. *et al.* shiny: Web Application Framework for R. (2023).

13. Pedersen, T. L., Nijs, V., Schaffner, T. & Nantz, E. A Server-Side File System Viewer for Shiny [R package shinyFiles version 0.9.3]. *CRAN Contrib. Packag.* (2022) doi:10.32614/CRAN.PACKAGE.SHINYFILES.

14. Yoo, A. B., Jette, M. A. & Grondona, M. SLURM: Simple Linux Utility for Resource Management. *Lect. Notes Comput. Sci. (including Subser. Lect. Notes Artif. Intell. Lect. Notes Bioinformatics)* **2862**, 44–60 (2003).

15. DI Tommaso, P. *et al.* Nextflow enables reproducible computational workflows. *Nat. Biotechnol. 2017 354* **35**, 316–319 (2017).

16. June 2024 | TOP500. https://top500.org/lists/top500/2024/06/.

17. Kurtzer, G. M., Sochat, V. & Bauer, M. W. Singularity: Scientific containers for mobility of compute. *PLoS One* **12**, e0177459 (2017).

18. Andrews. FastQC: a quality control tool for high throughput sequence data. (2010).

19. Ewels, P., Magnusson, M., Lundin, S. & Käller, M. MultiQC: summarize analysis results for multiple tools and samples in a single report. *Bioinformatics* **32**, 3047–3048 (2016).

20. Krueger, F. Trim Galore. https://www.bioinformatics.babraham.ac.uk/projects/trim_galore/ (2012).

21. Dobin, A. *et al.* STAR: ultrafast universal RNA-seq aligner. *Bioinformatics* **29**, 15–21 (2013).

22. Ramírez, F. *et al.* deepTools2: a next generation web server for deep-sequencing data analysis. *Nucleic Acids Res.* **44**, W160–W165 (2016).

23. Zerbino, D. R., Johnson, N., Juettemann, T., Wilder, S. P. & Flicek, P. WiggleTools: parallel processing of large collections of genome-wide datasets for visualization and statistical analysis. *Bioinformatics* **30**, 1008–1009 (2014).

24. Liao, Y., Smyth, G. K. & Shi, W. featureCounts: an efficient general purpose program for assigning sequence reads to genomic features. *Bioinformatics* **30**, 923–930 (2014).

25. Love, M. I., Huber, W. & Anders, S. Moderated estimation of fold change and dispersion for RNA-seq data with DESeq2. *Genome Biol.* **15**, 550 (2014).

26. Varet, H., Brillet-Guéguen, L., Coppée, J.-Y. & Dillies, M.-A. SARTools: A DESeq2- and EdgeR-Based R Pipeline for Comprehensive Differential Analysis of RNA-Seq Data. *PLoS One* **11**, e0157022 (2016).

27. Ritchie, M. E. *et al.* limma powers differential expression analyses for RNA-sequencing and microarray studies. *Nucleic Acids Res.* **43**, e47–e47 (2015).

28. Wickham, H. *ggplot2: Elegant Graphics for Data Analysis*. (Springer-Verlag New York, 2016).

29. Larsson, J. & Gustafsson, P. A Case Study in Fitting Area-Proportional Euler Diagrams with Ellipses Using eulerr. in *Proceedings of International Workshop on Set Visualization and Reasoning* vol. 2116 84–91 ({CEUR Workshop Proceedings}, 2018).

30. Dusa, A. venn: Draw Venn Diagrams. (2020).

31. Gehlenborg, N. UpSetR: A More Scalable Alternative to Venn and Euler Diagrams for Visualizing Intersecting Sets. (2019).

32. Fisher, R. A. On the Interpretation of χ 2 from Contingency Tables, and the Calculation of P. *J. R. Stat. Soc.* **85**, 87 (1922).

33. Chung, N. C., Miasojedow, B. Z., Startek, M. & Gambin, A. Jaccard/Tanimoto similarity test and estimation methods for biological presence-absence data. *BMC Bioinformatics* **20**, 8–11 (2019).

34. Shen, L. & Sinai, M. GeneOverlap: Test and visualize gene overlaps. (2013).

35. Yu, G., Wang, L.-G., Han, Y. & He, Q.-Y. clusterProfiler: an R package for comparing biological themes among gene clusters. *Omi. A J. Integr. Biol.* **16**, 284–287 (2012).

36. Dolgalev, I. msigdbr: MSigDB Gene Sets for Multiple Organisms in a Tidy Data Format. (2019).

37. Baruzzo, G. *et al.* Simulation-based comprehensive benchmarking of RNA-seq aligners. *Nat. Methods* **14**, 135 (2016).

38. Perelo, L. W., Gabernet, G., Straub, D. & Nahnsen, S. How tool combinations in different pipeline versions affect the outcome in RNA-seq analysis. *NAR Genomics Bioinforma.* **6**, 20 (2024).

39. Sarantopoulou, D. *et al.* Comparative evaluation of full-length isoform quantification from RNA-Seq. *BMC Bioinformatics* **22**, 1–24 (2021).

40. Rapaport, F. *et al.* Comprehensive evaluation of differential gene expression analysis methods for RNA-seq data. *Genome Biol.* **14**, 1–13 (2013).

41. Botvinik-Nezer, R. *et al.* Variability in the analysis of a single neuroimaging dataset by many teams. *Nature* **582**, 84 (2020).

42. Moreau, D., Wiebels, K. & Boettiger, C. Containers for computational reproducibility. *Nat. Rev. Methods Prim.* **3**, 50 (2023).

43. Wratten, L., Wilm, A. & Göke, J. Reproducible, scalable, and shareable analysis pipelines with bioinformatics workflow managers. *Nat. Methods 2021 1810* **18**, 1161–1168 (2021).

44. Kim, Y. M., Poline, J. B. & Dumas, G. Experimenting with reproducibility: a case study of robustness in bioinformatics. *Gigascience* **7**, 1–8 (2018).

45. MerkelDirk. Docker. *Linux J.* (2014) doi:10.5555/2600239.2600241.

46. Wilkinson, L. Exact and approximate area-proportional circular venn and euler diagrams. *IEEE Trans. Vis. Comput. Graph.* **18**, 321–331 (2012).

47. Conway, J. R., Lex, A. & Gehlenborg, N. A More Scalable Alternative to Venn and Euler Diagrams for Visualizing Intersecting Sets [R package UpSetR version 1.4.0]. *Bioinformatics* **33**, 2938–2940 (2019).

48. Wickham, H. *ggplot2: Elegant graphics for data analysis (use R!)*. (Springer, 2009).

49. Boone, M. A. *et al.* Identification of a Novel FUS/ETV4 Fusion and Comparative Analysis with Other Ewing Sarcoma Fusion Proteins. *Mol. Cancer Res.* **19**, 1795–1801 (2021).

50. Dreher, R. D. & Theisen, E. R. Lysine specific demethylase 1 is a molecular driver and therapeutic target in sarcoma. *Front. Oncol.* **12**, 1076581 (2023).

51. Luo, W. *et al.* GSTM4 is a microsatellite-containing EWS/FLI target involved in Ewing's sarcoma oncogenesis and therapeutic resistance. *Oncogene* **28**, 4126–4132 (2009).

52. Kinsey, M., Smith, R. & Lessnick, S. L. NR0B1 is required for the oncogenic phenotype mediated by EWS/FLI in Ewing's sarcoma. *Mol. Cancer Res.* **4**, 851–859 (2006).

53. Bayanjargal, A. *et al.* The DBD-α4 helix of EWSR1::FLI1 is required for GGAA microsatellite binding that underlies genome regulation in Ewing sarcoma. *bioRxiv* 2024.01.31.578127 (2024) doi:10.1101/2024.01.31.578127.

54. OpenAI. ChatGPT (Feb 12 version). (2024).

55. Moutsopoulos, I., Williams, E. C. & Mohorianu, I. I. bulkAnalyseR: an accessible, interactive pipeline for analysing and sharing bulk multi-modal sequencing data. *Brief. Bioinform.* **24**, 1–7 (2023).

Supplementary Table S1: A comparison of existing RNA-Seq pipelines with RNA-SeqEZPZ

	Starts from raw FASTQ files	Interface to select FASTQ	Ease of installation	Use of workflow managers?	Customization possible?	Features
**RNA-SeqEZPZ**	✔	✔	✔ All software is packaged into a Singularity container.	✔ Nextflow is used	✔	✔ Raw reads QC<br>✔ Differential expression<br>✔ Enrichment analysis<br>✔ comparative analysis across different conditions<br>✘ Gene regulatory network
**bulkAnalyseR**[55] **(2023)**	✘ Requires FASTQ files to be pre-processed as expression matrix.	✘	✘ Requires installation of dozens of R packages.	✘	✔	✘ Raw reads QC<br>✔ Differential expression<br>✔ Enrichment analysis<br>✘ comparative analysis across different conditions. Can only compare up to three conditions.<br>✔ Gene regulatory network
**ROGUE**[7] **(2023)**	✘ Requires FASTQ files to be pre-processed as count data.	✘	✔ No installation required. Everything is done on their server.	✘	✘ Everything is done on their server. No access to source code.	✘ Raw reads QC<br>✔ Differential expression<br>✔ Enrichment analysis<br>✘ comparative analysis across different conditions. Can only compare up to three conditions.<br>✘ Gene regulatory network

**ENCODE RNA-Seq[5] (2023)**	✓	✗	✓<br>All software is packaged into a Singularity container.	✓<br>WDL is used	✓	✓ Raw reads QC<br>✗ Differential expression<br>✗ Enrichment analysis<br>✗ comparative analysis across different conditions<br>✗ Gene regulatory network<br>✗ no interactive visualization
**RASflow[11] (2020)**	✓	✗	✓<br>All software is packaged into a Docker container.	✓<br>Snakemake is used	✓	✓ Raw reads QC<br>✓ Differential expression<br>✗ Enrichment analysis<br>✗ comparative analysis across different conditions<br>✗ no interactive visualization
**RaNA-Seq[10] (2020)**	✓<br>Requires the FASTQ files uploaded to their server.	✓	✓<br>No installation required. Everything is done on their server.	✗	✗<br>Everything is done on their server. No access to source code.	✗ Raw reads QC<br>✓ Differential expression<br>✓ Enrichment analysis<br>✗ comparative analysis across different conditions<br>✗ no interactive visualization
**nf-core/rnaseq[6] (2020)**	✓	✗	✓<br>All software is packaged into a Singularity container.	✓<br>Nextflow is used	✓	✓ Raw reads QC<br>✗ Differential expression<br>✗ Enrichment analysis<br>✗ comparative analysis across different conditions<br>✗ Gene regulatory network<br>✗ no interactive visualization

Figure 1
Click here to access/download;Figure;Fig_1_2.4.2.pdf

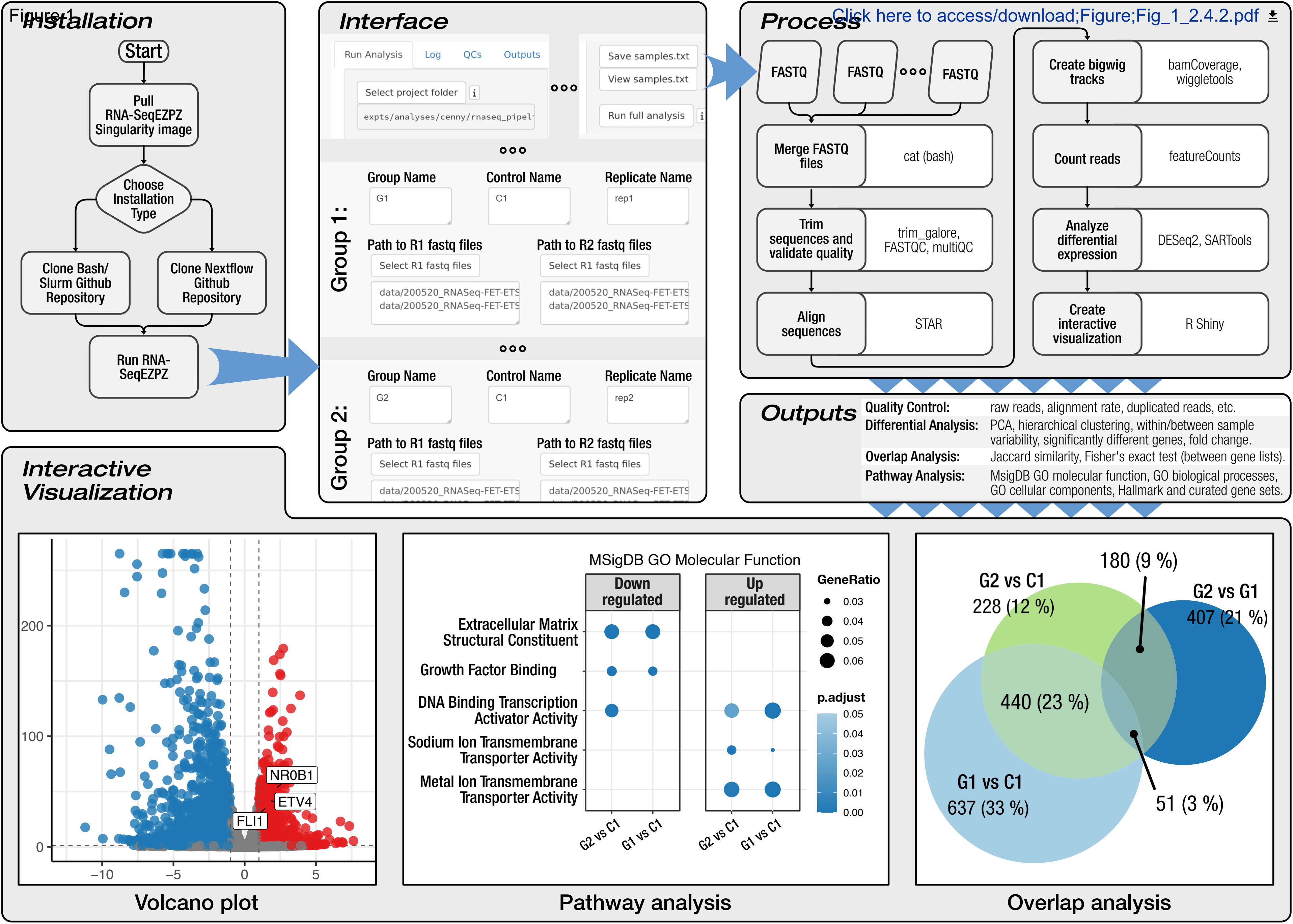

Figure 2

Click here to [illegible]1.2.0.pdf

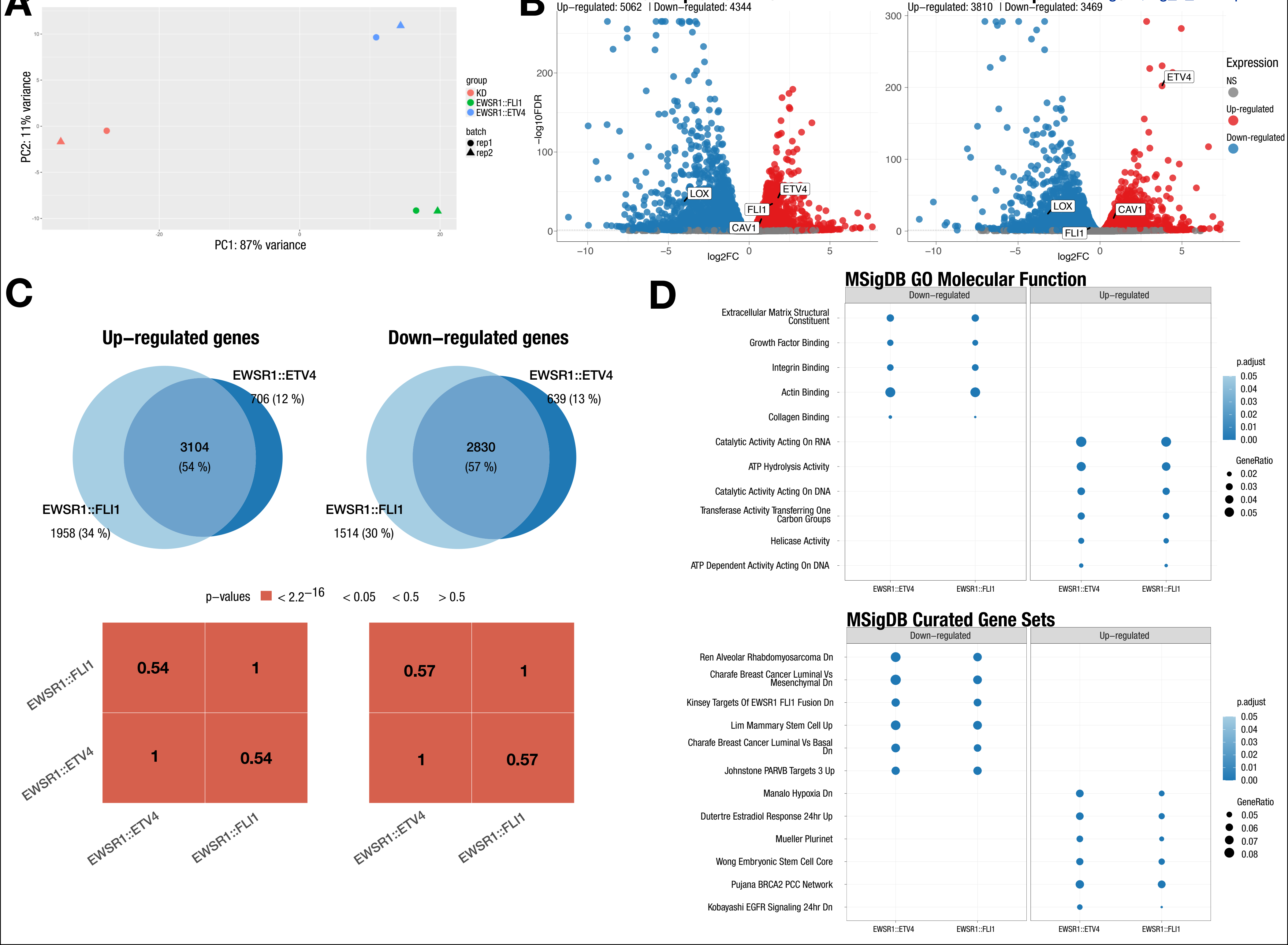

Figure 3

Click here to access/download;Figure;Fig_3_1.1.2.pdf

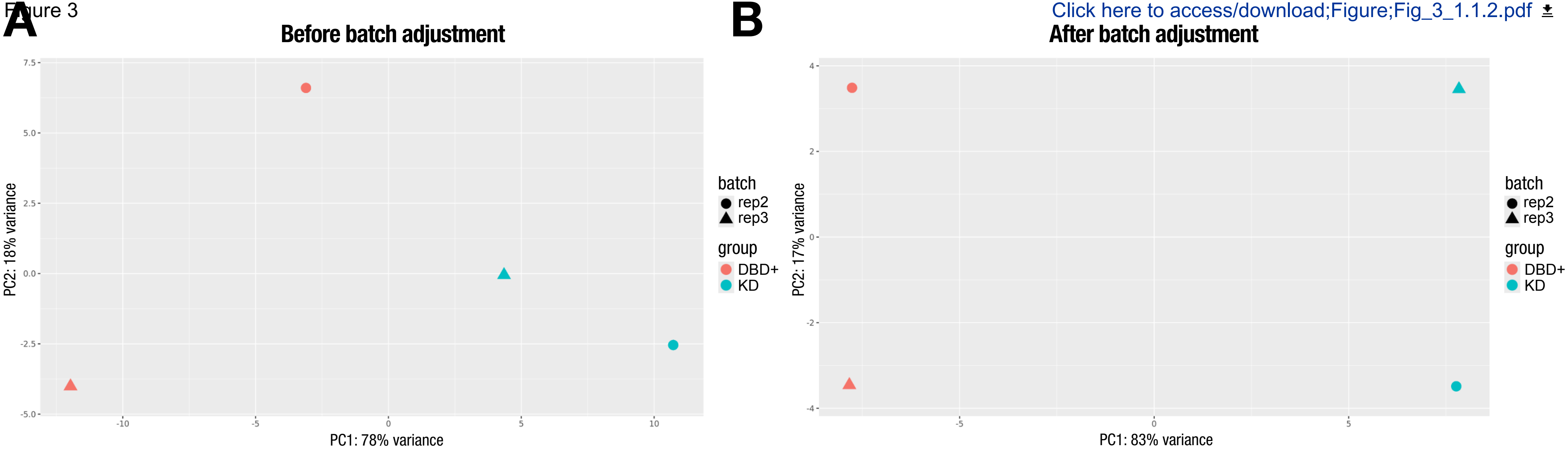

Supplementary Table S1

Click here to access/download
Supplementary Material
Suppplementary Table S1.docx

Supplementary Figure 1

Click here to access/download
Supplementary Material
Supplementary Figure 1.png

Supplementary Figure 2

Click here to access/download
Supplementary Material
Supplementary Figure 2.png

Supplementary Figure 3

Click here to access/download
Supplementary Material
Supplementary Figure 3.png

Supplementary Figure 4

Click here to access/download
Supplementary Material
Supplementary Figure 4.png

Supplementary Figure 5

Click here to access/download
Supplementary Material
Supplementary Figure 5.png

Supplementary Figure 6

Click here to access/download
Supplementary Material
Supplementary Figure 6.png

Supplementary Figure 7

Click here to access/download
Supplementary Material
Supplementary Figure 7.png

Supplementary Figure 8

Click here to access/download
Supplementary Material
Supplementary Figure 8.png

Supplementary Figure 9

Click here to access/download
Supplementary Material
Supplementary Figure 9.png

Supplementary Figure 10

Click here to access/download
Supplementary Material
Supplementary Figure 10.png

Supplementary Figure 11

Click here to access/download
Supplementary Material
Supplementary Figure 11.png

Supplementary Figure 12

Click here to access/download
Supplementary Material
Supplementary Figure 12.png

Supplementary File 2

Click here to access/download
Supplementary Material
Supplementary File 2.html

Click here to access/download
Supplementary Material
Supplementary File 3.pdf

Supplementary File 4

Click here to access/download
Supplementary Material
Supplementary File 4.txt

Cover letter Click here to access/download;Personal Cover;2025-02-17_CoverLetter_GigaScience.docx

NATIONWIDE CHILDREN'S
When your child needs a hospital, everything matters.℠

THE OHIO STATE UNIVERSITY
COLLEGE OF MEDICINE

February 17, 2025

Scott Edmunds, PhD
Editor-in-Chief
GigaScience

Dear Dr. Edmunds,

We are delighted to submit our manuscript titled "**RNA-SeqEZPZ: A Point-and-Click Pipeline for Comprehensive Transcriptomics Analysis with Interactive Visualizations**" for consideration by *GigaScience*.

In this manuscript, we present a comprehensive pipeline with end-to-end graphical user interface designed for the analysis of RNA-Seq experiments.

RNA-Seq analysis has become a routine experiment in many research laboratories. While several RNA-Seq pipelines such as nf-core (https://nf-co.re/rnaseq/3.17.0/) and ENCODE (https://www.encodeproject.org/data-standards/rna-seq/small-rnas/), are well established, none provide an easy-to-use interface combined with a comprehensive analysis. Additionally, as of this writing, Shiny-Seq, an RNA-Seq pipeline with a graphical interface (https://schultzelab.shinyapps.io/Shiny-Seq/), redirects to a non-existent page on FastGenomics, making it inaccessible. Our pipeline covers everything from raw FASTQ files to differential gene lists, providing a statistical report and offering interactive plots as well (https://github.com/cxtaslim/RNA-SeqEZPZ). It provides a user-friendly interface for bench scientists to perform their analysis, while allowing computational scientists to improve and adapt the scripts to fit their specific requirements. Furthermore, every step is fully documented and reproducible through containerization by singularity image ensuring consistent software versions. We also included a video tutorial.

Reproducibility remains a critical issue in bioinformatics, as it is crucial for reliable validation of scientific findings, as highlighted by Baykal et al. in "*Genomic reproducibility in the bioinformatics era*" (Genome Biology, 2024). We are confident that this pipeline will play a valuable role in enhancing the reproducibility of genomic results while empowering wet lab researchers to perform and visualize their own analysis. We believe our work aligns well with the journal's scope and will be of considerable interest to its readership.

We believe these individuals have the necessary expertise to thoroughly evaluate our work and offer valuable, objective feedback, and suggest them as reviewers:

Patrick Grohar, MD PhD
Professor
University of Michigan
grohar@med.umich.edu

Thomas G P Grünewald, MD PhD
Division Head, Translational Pediatric Sarcoma Research
Deutsches Krebsforschungszentrum (DKFZ)
t.gruenewald@kitz-heidelberg.de

Kenneth Chen, MD

Assistant Professor
UT Southwestern Medical Center
Kenneth.Chen@UTSouthwestern.edu

Adam Shlien, PhD
Professor
Hospital for Sick Children (SickKids)
adam.shlien@sickkids.ca

Thank you for your consideration of this manuscript.

Sincerely,

Emily Theisen, PhD
Principal Investigator, Nationwide Children's Hospital
Assistant Professor, The Ohio State University College of Medicine

Genevieve Kendall, PhD
Principal Investigator, Nationwide Children's Hospital
Assistant Professor, The Ohio State University College of Medicine
