# Supplementary material for: RNA-SeqEZPZ: a point-and-click pipeline for comprehensive transcriptomics analysis with interactive visualizations
Source: Gigascience. 2025 Nov 12;15:giaf133. doi: 10.1093/gigascience/giaf133 (PMC12857227; doi:10.1093/gigascience/giaf133)
Supplement: giaf133_GIGA-D-25-00067_Revision_2 [file giaf133_giga-d-25-00067_revision_2.pdf]

GigaScience

RNA-SeqEZPZ: A Point-and-Click Pipeline for Comprehensive Transcriptomics Analysis with Interactive Visualizations

--Manuscript Draft--

Manuscript Number:	GIGA-D-25-00067R2
Full Title:	RNA-SeqEZPZ: A Point-and-Click Pipeline for Comprehensive Transcriptomics Analysis with Interactive Visualizations
Article Type:	Technical Note
Funding Information:	American Cancer Society — Dr. Emily R. Theisen<br>Unravel Pediatric Cancer (RSG-22-118-01-DMC) — Dr. Emily R. Theisen<br>National Cancer Institute (R01 CA272872) — Dr. Genevieve C. Kendall<br>Alex's Lemonade Stand Foundation for Childhood Cancer (A award) — Dr. Genevieve C. Kendall<br>CancerFree KIDS (new idea award) — Dr. Genevieve C. Kendall<br>Research Institute, Nationwide Children's Hospital (startup fund) — Dr. Genevieve C. Kendall, Dr. Emily R. Theisen<br>V Foundation for Cancer Research (V scholar award) — Dr. Genevieve C. Kendall

Funder	Recipient
American Cancer Society	Dr. Emily R. Theisen
Unravel Pediatric Cancer (RSG-22-118-01-DMC)	Dr. Emily R. Theisen
National Cancer Institute (R01 CA272872)	Dr. Genevieve C. Kendall
Alex's Lemonade Stand Foundation for Childhood Cancer (A award)	Dr. Genevieve C. Kendall
CancerFree KIDS (new idea award)	Dr. Genevieve C. Kendall
Research Institute, Nationwide Children's Hospital (startup fund)	Dr. Genevieve C. Kendall<br>Dr. Emily R. Theisen
V Foundation for Cancer Research (V scholar award)	Dr. Genevieve C. Kendall

Abstract:

Background

RNA-Seq analysis has become a routine task in numerous genomic research labs, driven by the reduced cost of bulk RNA sequencing experiments. These generate billions of reads that require easy-to-run, comprehensive, and reproducible analysis. However, many labs rely on in-house scripts, which can be challenging for bench scientists to use and hinder standardization and reproducibility. While existing RNA-Seq pipelines attempt to address these challenges, they often lack a complete end-to-end user interface.

Findings

To bridge this gap, we developed RNA-SeqEZPZ, an automated pipeline with a user-friendly point-and-click interface, enabling rigorous and reproducible RNA-Seq analysis without requiring programming or bioinformatics expertise. For advanced users, the pipeline can also be executed from the command line, allowing customization of steps to suit specific applications. The innovation of this pipeline lies in the combination of three key features: (1) all software is packaged within a Singularity container, eliminating installation issues, (2) it offers a point-and-click interface from raw FASTQ files through differential expression and pathway analysis, and (3) it includes a Nextflow version, enabling scalability and portability for seamless execution across various platforms including job submission in the cloud and cluster computing. Additionally, RNA-SeqEZPZ generates a thorough statistical report and offers an option for batch adjustment to minimize effects of noise due to technical variations across replicates. Reports can also be reviewed by a bioinformatician to ensure the overall quality of the analysis.

Conclusions

RNA-SeqEZPZ is a robust, accessible, and scalable solution for comprehensive RNA-Seq analysis, enabling researchers to focus on biological insights rather than computational challenges.

Corresponding Author: Cenny Taslim, Ph.D.
Abigail Wexner Research Institute at Nationwide Children's Hospital
Columbus, OH UNITED STATES

Powered by Editorial Manager® and ProduXion Manager® from Aries Systems Corporation

**Corresponding Author Secondary Information:**	
**Corresponding Author's Institution:**	Abigail Wexner Research Institute at Nationwide Children's Hospital
**Corresponding Author's Secondary Institution:**	
**First Author:**	Cenny Taslim, Ph.D.
**First Author Secondary Information:**	
**Order of Authors:**	Cenny Taslim, Ph.D.
	Yuan Zhang
	Galen Rask
	Genevieve C. Kendall, Ph.D.
	Emily R. Theisen, Ph.D.
**Order of Authors Secondary Information:**	
**Response to Reviewers:**	Please see the attached point-by-point response to the reviewers.
**Additional Information:**	
**Question**	**Response**
Are you submitting this manuscript to a special series or article collection?	No
**Experimental design and statistics**<br><br>Full details of the experimental design and statistical methods used should be given in the Methods section, as detailed in our Minimum Standards Reporting Checklist. Information essential to interpreting the data presented should be made available in the figure legends.<br><br>Have you included all the information requested in your manuscript?	Yes
**Resources**<br><br>A description of all resources used, including antibodies, cell lines, animals and software tools, with enough information to allow them to be uniquely identified, should be included in the Methods section. Authors are strongly encouraged to cite Research Resource Identifiers (RRIDs) for antibodies, model organisms and tools, where possible.	Yes

Powered by Editorial Manager® and ProduXion Manager® from Aries Systems Corporation

Have you included the information requested as detailed in our Minimum Standards Reporting Checklist?	
**Availability of data and materials**<br><br>All datasets and code on which the conclusions of the paper rely must be either included in your submission or deposited in publicly available repositories (where available and ethically appropriate), referencing such data using a unique identifier in the references and in the "Availability of Data and Materials" section of your manuscript.<br><br>Have you have met the above requirement as detailed in our Minimum Standards Reporting Checklist?	Yes
GigaScience has policies and guidelines in place for the use of generative AI-writing tools such as ChatGPT. If you have used such writing tools to assist with writing the manuscript this must be declared and cited in the text. Authors should not list AI-writing tools and other AI-assisted technologies as an author or co-author and should acknowledge that they are fully responsible for text generated or refined by AI-writing tools.<p><br><br>A summary of use (particularly in the introduction or among methods) needs to be included at the end of the paper, and the outputs should also be included as a supplementary file hosted in GigaDB or other open repositories. Please read our guidelines for more information. <p><br><br>By submitting to GigaScience, you are aware of the journal's AI-writing tools policy, and if you have declared use of such tools below, you have acknowledged this where appropriate in your manuscript and have made a summary of use and outputs available. <p><br><b>AI-assisted writing tools have been used in the preparation of this manuscript?	Yes

Manuscript

Click here to access/download;Manuscript;RNAseq pipeline paper_final_16 (1).docx

RNA-SeqEZPZ: A Point-and-Click Pipeline for Comprehensive Transcriptomics Analysis with Interactive Visualizations

Cenny Taslim[1]*, Yuan Zhang[2]*, Galen Rask[1], Genevieve C. Kendall[1,3†], Emily R. Theisen[1,3†hay]

[1]Center for Childhood Cancer Research, The Abigail Wexner Research Institute, Nationwide Children's Hospital, Columbus, OH 43215, USA.

[2]High Performance Computing Center, The Abigail Wexner Research Institute, Nationwide Children's Hospital, Columbus, OH 43215, USA.

[3]Department of Pediatrics, The Ohio State University College of Medicine, Columbus, OH 43210, USA.

*, † authors contributed equally to this work

† Corresponding authors: Genevieve C. Kendall (Genevieve.Kendall@NationwideChildrens.org) and Emily R. Theisen (Emily.Theisen@NationwideChildrens.org)

ORCID

Cenny Taslim[0000-0003-3302-9099];

Yuan Zhang; Galen Rask[0000-0002-6877-4796];

Genevieve C Kendall [0000-0003-3775-2006];

Emily R Theisen [0000-0003-2923-1198]

Abstract

Background

RNA-Seq analysis has become a routine task in numerous genomic research labs, driven by the reduced cost of bulk RNA sequencing experiments. These studies generate billions of reads that require easy-to-run, comprehensive, and reproducible analysis. However, many labs rely on in-house scripts, which can be challenging for bench scientists to use and hinder standardization and reproducibility. While existing RNA-Seq pipelines attempt to address these challenges, they often lack a complete end-to-end user interface.

Findings

To bridge this gap, we developed RNA-SeqEZPZ, an automated pipeline with a user-friendly point-and-click interface, enabling rigorous and reproducible RNA-Seq analysis without requiring programming or bioinformatics expertise. For advanced users, the pipeline can also be executed from the command line, allowing customization of steps to suit specific applications. The innovation of this pipeline lies in the combination of three key features: (1) all software is packaged within a Singularity container, eliminating installation issues, (2) it offers a point-and-click interface from raw FASTQ files through differential expression and pathway analysis, and (3) it includes a Nextflow version, enabling scalability and portability for seamless execution across various platforms including job submission in the cloud and cluster computing. Additionally, RNA-SeqEZPZ generates a thorough statistical report and offers an option for batch adjustment to minimize effects of noise due to technical variations across replicates. Reports can also be reviewed by a bioinformatician to ensure the overall quality of the analysis.

Conclusions

RNA-SeqEZPZ is a robust, accessible, and scalable solution for comprehensive RNA-Seq analysis, enabling researchers to focus on biological insights rather than computational challenges.

Introduction

Data analysis of RNA-Seq consists of a set of successive stages that are repetitive and routinely executed using a wide variety of tools. Typically, analysis starts with quality control of raw sequence reads or FASTQ files followed by alignment of reads to a reference genome, filtering of low-quality reads, counting reads that align to a specific feature/gene, differential analysis of genes in different conditions and finally visualization of the results [1]. In-house analysis usually involves a bioinformatician creating step-by-step scripts for specific datasets which will need to be modified for different datasets. With each modification and customization, it is notoriously challenging to keep the analysis fully reproducible primarily due to differences in scripts, hardware, operating systems, and software versions. Reproducibility is critical for a rigorous analysis to ensure reliable validation of scientific findings and has long been a challenging issue in biomedical research [2]. A recent publication found that a large majority of existing Jupyter notebooks (a popular format for documenting and sharing computational workflow) could not be executed automatically and failed to reproduce the results[3]. Reproducibility issues have even led to a retraction of an epidemiological paper [3].

Furthermore, wet lab scientists who conduct the RNA-Seq experiments and generate libraries often have limited programming and bioinformatics experience, making it challenging for them to analyze their own data efficiently while ensuring statistical rigor and reproducibility. This creates a strong demand for an easy-to-use, comprehensive pipeline that expedites routine RNA-Seq analysis without sacrificing the quality and reproducibility of the results. Here, we describe RNA-SeqEZPZ [4,5], a point-and-click tool for comprehensive analysis of RNA-Seq experiments from FASTQ to result visualization. RNA-SeqEZPZ is primarily designed to empower bench scientists to do their own analyses and explore their results while also providing bioinformaticians with the flexibility for further customization.

Several RNA-Seq pipelines exist, with ENCODE[5] and nf-core [6] among the most widely used in the community. In comparison to these pipelines, a notable feature of RNA-SeqEZPZ is its point-and-click interface starting from FASTQ files up to differential genes analysis and interactive visualization capabilities. ENCODE does not perform differential genes analysis and has no interactive visualization. The nf-core RNA-Seq pipeline itself does not include built-in interactive visualization and differential gene analysis. However, it provides output files that can be used as input to a separate visualization and differential analysis pipeline that must be run independently using command line after the completion of the RNA-seq pipeline. Several shiny [7] apps providing a graphical interface for RNA-Seq analysis such as ROGUE[8], Shiny-Seq [9] and bulkAnalyseR [10] have also been previously published. However, these tools do not support analyzing RNA-Seq experiments starting from raw FASTQ files. Furthermore, at the time of writing, Shiny-Seq appears to be no longer accessible, as its official website [11] redirects to a "not found" page on FastGenomics. Access to ROGUE online [12] was repeatedly interrupted by server issues, which may impact its usability for analysis. We found that Partek™ flow and RaNA-Seq [13] offers functionalities most similar to RNA-SeqEZPZ. However, both require users to upload FASTQ files to their server, which can be complicated by connection and firewall restrictions or create privacy concerns if analyzing patient data. In addition, neither pipeline provides access to full source code, limiting customization. RASflow [14] supports analysis from FASTQ files but lacks a graphical interface for selecting these files, which may hinder usability for non-technical users. Regarding comparative analysis, only bulkAnalyseR and RaNA-seq appear to support such feature. However, bulkAnalyseR restricts comparisons to a maximum of two groups, whereas RNA-SeqEZPZ supports comparisons across up to 7 groups. In RaNA-seq, comparative analysis is limited to a Venn diagram of significant gene overlap. In contrast, RNA-SeqEZPZ offers an expanded suite of analysis, including Venn diagrams, gene overlap analysis and pathway comparisons across groups. A comparison of these tools is provided in Supplementary Table S1.

To the best of our knowledge, RNA-SeqEZPZ is the first open-source tool to offer a point-and-click interface with interactive plots, starting from raw FASTQ reads and providing analytical capabilities from differential genes analysis to pathway analysis. This pipeline can potentially accelerate research progress by simplifying a complex process, enhancing reproducibility within and across labs, and empowering researchers with the tools to interpret their own results. With the extensive reports generated by the pipeline, a bioinformatician can supervise the entire process by reviewing the reports to ensure accuracy and proper execution.

Methods

RNA-SeqEZPZ can be started using a single command after downloading a Singularity image and cloning the Git repository (Figure 1 and Supplementary Figure 1). It encompasses multiple steps, utilizes various tools, and generates statistical reports, visualization, and diverse output files. The pipeline accepts gzipped paired-end FASTQ files as input and supports analysis for 20 genomes including human, zebrafish, and mouse. Users can select all the inputs through a point-and-click interface implemented using a shiny [7] app and shinyFiles [15] allowing them to initiate a comprehensive analysis effortlessly.

Figure 1. Overview of RNA-SeqEZPZ workflow, interface, and analysis outputs. Installation begins with pulling a Singularity image and cloning a git repository. After installation, the software can be run with a single command, which launches a web interface allowing users to select parameters and FASTQ files. Clicking “Run full analysis” triggers multiple processes that generate statistical outputs and provide interactive visual interfaces.

Software Implementation

RNA-SeqEZPZ is a combination of a shiny [7] app with either bash scripts and SLURM[16] (a cluster resource management system) or Nextflow [17], a workflow management system (Figure 1). The shiny app at the front end provides an interface for users to run the entire analysis. As SLURM is the most widely used workload manager in High-Performance Computing (HPC) [18], using it in bash scripts will enable users to easily modify the scripts as needed and leverage their existing familiarity with the system. Nextflow is a modern workflow management system designed to simplify the development and deployment of complex data analysis pipelines. Nextflow enhances the flexibility of this pipeline to run on diverse computation infrastructures with workload managers other than SLURM. The required R packages, and all other tools needed for analysis including Firefox (the browser used for the interface) are enclosed inside a Singularity [19] container removing any potential difficulties involved in the installation of all the required software. Altogether this promotes the reproducibility, standardization, and portability of the RNA-SeqEZPZ pipeline. Further, because the shiny app and analysis can be run locally on a cluster, there is no need to transfer gigabytes to terabytes of data to an external server over the internet.

Installation and Usage

Installation instructions are provided in detail at the GitHub repository. Briefly, a Singularity image either a Nextflow-based or a bash/SLURM-based version, depending on user preference containing all the necessary scripts is cloned from a repository (Figure 1). To use the pipeline, users need to connect to their HPC cluster and run a one-line command: “`bash run_shiny_analysis.sh`” which will bring up a Firefox browser (provided by the container) where the user will be able to select the sample FASTQ file path, output path, resource requirements, and various settings. Options are also available for running the steps of the pipeline individually (see the manual on the website for details). To assist users running this for the first time, we have provided example datasets that can be downloaded from the

GitHub repository, along with an easy-to-follow step-by-step tutorial. A video tutorial is available in Supplementary File 5.

Workflow Overview

RNA-SeqEZPZ performs multiple steps. The process begins with merging FASTQ files from different sequencing lanes using cat command in Bash. Raw reads quality control is assessed using default metrics provided by FASTQC [20] and the quality control reports are compiled using MultiQC [21]. For guidance on interpreting FASTQC metrics to identify and remove low quality files, users may refer to thresholds commonly applied in variant calling analysis [22]. Low quality bases and adapter sequences are removed using trim_galore [23]. Specifically, bases with a Phred [24] quality score below 20 are trimmed from the 3' end of the reads. Paired-end reads that become shorter than 20 bp after trimming are discarded. Following quality control and trimming, reads are aligned to the reference genome using the two-pass approach of STAR [25], which enhances mapping accuracy. Subsequently, gene-level read quantification is carried out using featureCounts [26]. BigWig tracks are generated using bamCoverage [27] and WiggleTools [28] for visualization. Differential expression analysis is performed using DESeq2 [29] with batch adjustment, and statistical reports are generated by SARTools [30]. By default, differentially expressed genes are identified using a False Discovery Rate (FDR) [31] threshold of 0.05, with no fold-change cut-off applied. These thresholds along with the minimum difference in normalized count can be adjusted by users through the graphical interface (see Supplementary Figure 6). The model incorporates replicates as a covariate to correct for batch effects. Users also have the option to disable batch adjustment directly within the interface (see Supplementary Figure 2). In the PCA plot generated by the pipeline, the effect of batch adjustment is estimated using limma [32].

Interactive Visualization

To provide additional insights into gene expression analysis, RNA-SeqEZPZ includes several interactive visualization tools. Volcano plots are generated using ggplot2 [33] to highlight differentially expressed genes. Area-proportional Euler and Venn diagrams, along with UpSet plots, are generated using Eulerr [34], venn [35] and UpSetR [36] to visualize gene overlaps. The significance of overlap is assessed by testing the independence of two variables using Fisher's exact test [37]. Additionally, the Jaccard Index [38] which quantifies the similarity between gene lists, is computed using GeneOverlap [39] package. For pathway analysis, over-representation analysis is conducted using clusterProfiler [40], utilizing gene sets annotations from MSigDB via the msigdbr [41] package. These interactive tools provide deeper insights into gene expression functions and biological significance.

Rationale for tool selection

RNA-SeqEZPZ is designed as an easy-to-use and accessible pipeline for researchers with no prior experience in RNA-Seq analysis. To ensure simplicity, a single tool is selected for each step based on best practices and recommendations from the Hitchhiker's Guide [42]. For advanced users, the code is fully accessible, allowing customization, tool substitution, and modifications as needed.

For read alignment, STAR [25] was chosen due to its high performance RNA-Seq mapping capabilities [43]. The alignment process occurs in two stages: first, initial mapping identifies potential novel splice junctions, followed by a refined alignment using both known annotations and the newly detected junctions. These two steps approach enhances read mapping accuracy and improves sensitivity.

In our pipeline, we focus on quantifying reads at the gene level, as all isoforms of the same gene typically share the same pathway annotations. To achieve this, we selected featureCounts [26], a fast and efficient quantification of mapped RNA-Seq based on genome alignment. Additionally, a comparative evaluation of seven widely used quantification algorithms demonstrated that

featureCounts [26] has higher sensitivity in detecting single-isoform genes while delivering comparable performance on real datasets [44,45].

For differential expression analysis, DESeq2 was selected based on findings by Rapaport et al. (2013) [46], which demonstrated its superior specificity and sensitivity as well as good control of false positive errors. More recently, the bestDEG [47] study further support DESeq2's enhanced sensitivity compared to other tools when applied to human RNA-seq datasets from MicroArray Quality Control (MAQC) project. In addition, DESeq2 addresses batch effects by incorporating batch variables as covariates within its Generalized Linear Model (GLM) design formula, thereby removing unwanted technical variation.

Reproducibility

Reproducibility has long been a key issue in bioinformatics analysis [48,49]. Ensuring the ability to execute an existing workflow and reproduce the same exact results is crucial for advancing scientific research [50]. To achieve this goal, we employed several solutions following best practices[51–53] to ensure RNA-SeqEZPZ is highly reproducible.

Software Containerization

To prevent dependency mismatches and ensures consistency across computational environments, we encapsulated all software dependencies within a Singularity [19] container. This guarantees that RNA-SeqEZPZ can be used across multiple environments including local machines, cloud platforms, or High Performance Computing (HPC) cluster eliminating issues caused by dependency mismatches. Unlike Docker [54], another popular containerization platform that requires root privileges, Singularity [19] operates without the need for elevated permission, making it ideal in shared environment such as HPC clusters. Additionally, using Singularity [19] eliminates manual installation of software on different systems and ensures it yields the same results on different machines.

Workflow Documentation

Beyond software dependencies, Kim et al. [53] emphasize the importance of comprehensive documentation and readable code for ensuring reproducibility. Documenting analysis steps and software can be challenging, as bioinformatics workflows often consists of a multitude of tools and steps which are chained together to create a complex analysis workflow. Additionally, minimizing manual steps that are required to execute an analysis workflow is crucial, which is why computational pipelines are needed to automate the integration and execution of these tools.

RNA-SeqEZPZ implemented as a Bash-based pipeline, is designed for readability and ease of use. It automates workflow execution, supports the re-analysis of failed runs, and generates comprehensive documentation on data processing ensuring transparency, code sharing and long-term reproducibility. However, tasks such as re-analysis of failed runs and documentation must be implemented manually. To further enhance flexibility, automation, and resource management, RNA-SeqEZPZ leverages Nextflow [17], a powerful bioinformatics workflow manager. Nextflow have been recognized as key solutions for achieving reproducibility by standardizing execution, tracking inputs and consistent runtime setting [52]. Beyond ensuring reproducibility, Nextflow enables easy parallelization, job-scheduling, re-analysis of failed runs, seamless integration of software containerization and efficient resource management. Additionally, it automates generation of execution report with detailed information, such as input parameters to the pipeline, software versions, and resource usage information, further optimizing workflow efficiency and reproducibility [52].

Code Sharing and Accessibility

To promote transparency and reproducibility, we ensure that all code is publicly accessible via an online repository such as GitHub. This allows other researchers to review, modify, and extend RNA-SeqEZPZ,

fostering collaboration and long-term sustainability. The integration of these solutions collectively ensures that RNA-SeqEZPZ maintains a high level of reproducibility.

Below, we describe in more detail the components of the RNA-SeqEZPZ interface, including interactive plots implemented using Shiny [55].

User Friendly Interface and Generated Outputs

A primary design goal of RNA-SeqEZPZ is to accelerate full analysis of RNA-seq datasets and provide interactive analysis of the results. As such, the pipeline is designed to be run with a one-line command in the terminal that loads a user-friendly interface implemented as a Shiny [55] app (Figure 1).

The interface is accessed through a Firefox browser, allowing users to easily zoom in or out, enlarge text, and adjust the window size for better visibility. To run the analysis, users simply select their FASTQ files and provide the necessary information through an intuitive file browser interface (Supplementary Figure 2). After entering all sample information, clicking "Run full analysis" will automatically execute the full analysis as described above (Figure 1).

During the analysis, users can monitor progress through the "Log" tab (Supplementary Figure 3). Upon completion, the `run_rnaseq_full.out` log file will display the message "Done running RNA-seq full analysis". The files in the "Log" tab display the current step being processed by the pipeline. Once the analysis is completed, users will be able to click on the "QC" tab and see all the quality control metrics compiled by MultiQC [21] (Supplementary Figure 4). The MultiQC [21] generated HTML files are interactive as well, which allows for some customization of the plots (Supplementary File 1). The QC report includes metrics for raw reads, alignment rate, number of duplicated reads, percentage of reads aligned to genomic features, etc. A statistical report of the differential gene analysis can be viewed in

the “Outputs” tab (Supplementary Figure 5). This report is generated using a modified version of SARTools [30]. The report contains description of raw data, Principal Component Analysis (PCA) plot and hierarchical clustering of samples to explore the variability within and between samples. The statistical report also describes the steps performed in the differential analysis using DESeq2 [56] along with the statistical assumptions and validation of the choices used (Supplementary File 2).

Under “Plots” tab users can adjust the cut-offs for significant differential genes and in table, they can find the $\log_2$ fold-change of their gene of interest (Supplementary Figure 6), create volcano and UpSet plots (Supplementary Figure 7 and Supplementary Figure 8), perform overlap (Supplementary Figure 9) and pathway analysis (Supplementary Figure 10). The GeneOverlap [39] package is utilized to compute the Jaccard similarity index [38] and Fisher’s exact test [37] to evaluate the significance of overlap between the gene lists (Figure 2C). The overlaps between genes in different conditions were visualized using proportional Euler and Venn diagrams, as well as an UpSet plot, created using eulerr [57], Venn [35] and UpSetR [58] packages. Pathway analysis or Over-Representation analysis was conducted using clusterProfiler [40] and msigdbr [41] packages. All other plots were generated using ggplot2 [59] package.

Additionally, since the files, including intermediate ones generated by the pipeline can accumulate to terabytes in size, we provide users a simple way to delete projects and files they no longer require (Supplementary Figure 11). To assist in this process, we provide explanations to help users determine whether to keep or delete these files.

Furthermore, in our Nextflow version, we provide an interface to view the report generated by Nextflow (Supplementary Figure 12 and Supplementary File 6).

Public dataset analysis

To demonstrate the utility of our pipeline, we re-analyzed the RNA-seq experiments in the study of novel Ewing Sarcoma fusion proteins [60]. RNA-SeqEZPZ was run on two biological replicates from a knockdown/rescue experiment in the A673 human cell line where the endogenous fusion oncogenic transcription factor EWSR1::FLI1 was depleted by shRNA and then rescued with either EWSR1::FLI1 or EWSR1::ETV4 constructs. These samples were compared to control cells with no rescue (KD). The FASTQ files can be downloaded from GEO (GSE173185).

As shown in the QC report, for EWSR1::ETV4 rescued sample replicate 1, there are 48.5 million aligned reads (83.4% alignment rate) and 53.7% of these reads are assigned to a feature (Supplementary File 1). The PCA plot in the statistical report shows that the 6 samples cluster first by replicates and then by rescue condition. This suggests that experimental conditions significantly influence the observed variability, and that the samples within each replicate group are highly similar, indicating good reproducibility (Figure 2). Differential genes were identified with FDR < 0.05. In samples where EWSR1::FLI1 was rescued, FLI1 is correctly up-regulated, serving as a surrogate for the EWSR1::FLI1 fusion. In samples where EWSR1::FLI1 was knocked down and then rescued with an EWSR1::ETV4 construct, it shows FLI1 as down-regulated and ETV4 as up-regulated genes compared to knockdown control (Figure 2). Well known targets of EWSR1::FLI1 such as LOX1 and CAV1 [61,62] are shown as down- and up-regulated in both EWSR1::FLI1 and EWSR1::ETV4 rescued samples. There is significant overlap between genes up-regulated (3,104 genes, p-values < 0.05) and genes down-regulated (2,830 p-value < 0.05) by both EWSR1::FLI1 and EWSR1::ETV4 suggesting that EWSR1::ETV4 regulates similar genes as EWSR1::FLI1. Consistent with overlap analysis that shows significant overlap between genes, the pathway analysis indicates that genes regulated by EWSR1::ETV4 and EWSR1::FLI1 are involved in many similar functions (Figure 2 and Supplementary File 3). EWSR1::FLI1 downregulated genes are consistent with those identified in a previous study by Kinsey *et al.* [63] (Supplementary File 3). The QC report (Supplementary File 1) and statistical report of the differential analysis (Supplementary File 2) are

saved as HTML files. All the plots created in RNA-SeqEZPZ can be exported as a pdf file (Supplementary File 3). One of the widely used outputs for downstream analysis is the list of differentially expressed genes. These tables list genes that are defined as significant along with their Ensembl ID, raw and normalized read count, fold-changes, p-values adjusted for multiple testing, and other statistics generated by the DESeq2 models (Supplementary File 4). Video tutorial on the analysis of this dataset is included in Supplementary File 5.

Figure 2. Analysis results of samples rescued with EWSR1::FLI1 and EWSR1::ETV4 constructs. (A) PCA plot showing good separation between the two different conditions. (B) Volcano plots for the two rescue constructs showing highlighted known targets of EWSR1::FLI1 in addition to FLI1 and ETV4 indicative of the rescue conditions. (C) Overlap analysis reveals a significant overlap between genes regulated by the two constructs. Box colors indicate p-values of overlaps, while the number inside the boxes represent the Jaccard Similarity Index.

Portability, Scalability and Reproducibility of Results

By using RNA-SeqEZPZ instead of in-house scripts, users can more easily run analyses across diverse computational infrastructures with a range of hardware architectures and CPU configurations, while also handle large datasets efficiently. To demonstrate this, we ran RNA-SeqEZPZ on three independent datasets across two distinct computing environments: (1) the HPC Facility at Abigail Wexner Research Institute (AWRI), and (2) the Ohio Supercomputer Center (OSC) [64]. First, we re-analyzed the knockdown/rescue experiments of EWSR1::FLI1 and EWSR1::ETV4, each with two replicates and approximately 50 to 60 million paired-end reads (~134 GB total), as previously described (GEO GSE173185). The analysis ran on two different HPC clusters with the same cut-offs produced identical results, identifying 5,062 up-regulated and 4,344 down-regulated genes by EWSR1::FLI1. Notably, these runs were performed over a year apart on April 30, 2024 at AWRI and on June 11, 2025 at OSC (see

Supplementary File 2 for the AWRI run and Supplementary File 7 for the OSC run). Second, we analyzed RNA-seq data from A673 cells treated with either vehicle control (DMSO, n = 3) or HCI 2509, an KDM1A inhibitor which has been shown to reverse the transcriptional activity of EWSR1::FLI1 (n = 3) [65]. Additionally, we included EWSR1::FLI1 knockdown cells (iEF) cells and RNAi luciferase controls (iLuc), each in quadruplicates (n = 4) [66]. The dataset comprises approximately 130 GB of raw FASTQ files, which are available for download from GEO under accession number GSE98787 and GSE94503. Both analyses identified a total of 9,797 differentially expressed genes with FDR ≤ 0.05, including down-regulation of KDM1A in cells treated with HCI 2509 compared to control cells (see Supplementary Figure 13). The analyses were executed at OSC using 30 CPUs and completed in 1 hour 59 minutes. In comparison, the same analysis at AWRI ran with 20 CPUs, finished in 2 hours 7 minutes (see Supplementary File 8 for the AWRI run and Supplementary File 9 for the OSC run). Finally, we analyzed RNA-seq from 6 hours post-fertilization (hpf) zebrafish embryos injected with human PAX3::FOXO1 compared to control injected embryos, each in quadruplicates [67] (GEO accession: GSE270325), to investigate the in vivo activity of the fusion gene. The dataset is approximately 60GB. Reads were aligned to a custom reference genome that included the PAX3::FOXO1 sequence, enabling quantification of its expression. In both runs, PAX3::FOXO1 was identified as the most highly expressed gene, with a $\log_2$ fold-change of 14.59 relative to control, followed by tyrp1b, irx4a and pdia2 (Supplementary Figure 14).

We have run three RNA-seq datasets (from human and zebrafish samples), ranging from 60GB to 130GB, across two distinct computing environments with varying infrastructures and CPU configurations. All runs produced identical results emphasizing the scalability and portability of RNA-SeqEZPZ while ensuring reproducibility of the results.

Effects of Batch Adjustment

In order to highlight the benefits of adjusting for batch effects, we re-analyzed RNA-seq experiments from the manuscript, "The DBD-α4 helix of EWSR1::FLI1 is required for GGAA microsatellite binding that underlies genome regulation in Ewing sarcoma" [68]. The FASTQ files were obtained from GEO (GSE268944). RNA-SeqEZPZ was used to analyze two biological replicates of knockdown/rescue experiments in the TTC-466, an Ewing Sarcoma cell line. Following knockdown of EWSR1::FLI1, rescue was performed using either a mutant construct (DBD+) or the full-length EWSR1::FLI1 construct (EF). Figure 3A presents the PCA plot before batch adjustment. Based on the plot, it is difficult to definitively determine whether the biological replicates cluster together. However, after adjusting for batch effect, the DBD+ replicates cluster together, separating from EWSR1::FLI1 samples along PC1, which accounts for 83% of the variance (Figure 3B). Furthermore, batch adjustment increased the variance explained by PC1 from 78% to 83%, further clarifying sample separation.

Figure 3. PCA plot demonstrating the impact of batch adjustment. (A) PCA plot before batch adjustment and (B) PCA plot after batch adjustment, showing the improved separation achieve through batch correction.

To further demonstrate the benefit of batch correction on a larger dataset, we reanalyzed RNA-seq data from A673 Ewing sarcoma cells reported in "Seclidemstat blocks the transcriptional function of multiple FET-fusion oncoproteins" [69]. Cells were treated with vehicle (DMSO) or seclidemstat at IC50 or IC90, and RNA-seq was performed in biological triplicate. These samples are available in the GEO (GSE306637). Seclidemstat is currently in clinical trials for FET-rearranged sarcomas (NCT03600649). Prior to batch correction, IC50 and IC90 samples were intermixed. Following batch correction, distinct

clusters emerge yielding clear dose specific clusters and increasing the variance explained by PC1 by 21% (Supplementary Figure 20).

RNA-SeqEZPZ offers adjustment to correct for technical differences introduced by processing replicates in batches. By default, the replicate name is treated as a batch variable and added to the Generalized Linear Model (GLM) as a covariate to be adjusted by DESeq2. Including replicates in the model allows DESeq2 to account for unwanted variation between replicates, effectively adjusting the read counts for each gene or feature. This adjustment helps reduce noise due to technical variability and increases the sensitivity for detecting differentially expressed genes between biological conditions [70]. When the data exhibits significant batch effects and the samples cluster primarily by batch rather than by biological condition of interest in the PCA plot (e.g. different instruments, sequencing runs, technical variations between replicates), it is recommended to perform batch adjustment. In general, when you have a balanced design where the number of replicates is equal across conditions, adjusting for replicate variation can enhance both the sensitivity and precision of the estimate. However, in the case of A673 cell line, which includes DMSO control (n = 3), HCI 2509 treatment (n = 3), iLuc control (n = 4) and EWSR1::FLI1 knockdown (iEF, n = 4), the unadjusted PCA plot already shows clear clustering by condition. Notably, the two control groups (DMSO and iLuc) cluster together as expected (Supplementary Figure 15). After applying replicate-based adjustment, replicate 5 of the iLuc group shifts closer to the DMSO cluster, and replicate 5 of the iEF group becomes more distant from the rest of the iEF replicates. Therefore, for this dataset, it may be best to do the analysis without adjusting for replicates, due to the lack of representation of replicate 5 across all conditions. Users can easily turn off this adjustment by unchecking the “Replicates batch adjustment” option in the user interface (Supplementary Figure 2). Batch correction for other variables or experimental factors can be carried out by modifying the scripts provided using either a multi-factor design in DESeq2 [29] or in combination with ComBat-seq [70].

Side-by-side Comparison with RaNA-seq

RNA-SeqEZPZ was created to enable bench scientists to run their own analysis from beginning processing of the raw FASTQ files to the differential gene analysis. Although other pipelines exist (see Supplementary Table 1), RaNA-seq [13] will be the most comparable to RNA-SeqEZPZ in terms of user-friendliness. RaNA-seq doesn't require pre-processing of the FASTQ files, provides a user interface for FASTQ files selection, and no intensive installation is required, while also performing differential gene analysis. Its main limitation, however, is the need to upload FASTQ files to a remote server, which can be challenging due to institutional firewalls or other security constraints.

To enable a side-by-side comparison between RNA-SeqEZPZ and RaNA-seq, we analyzed RNA-seq data from nuclear factor (erythroid-derived 2) knockout (Nrf2 KO) mice, which develop lung tumors earlier than wildtype (WT) mice (GEO GSE99338) [71].

QC reports

In RaNA-seq, the QC report contains boxplots of the normalized expression values (TPM), bar plot of estimated number of expressed genes, heatmap of expression similarity and PCA plot which are similar to the outputs report generated by RNA-SeqEZPZ (see Supplementary File 10 for RaNA-seq and Supplementary File 11, a similar report generated by RNA-SeqEZPZ). One notable difference between our pipeline and theirs is the use of batch adjustment. As a result of this adjustment, PC1 in our analysis explained 75% of the variance, compared to 46.4% in their analysis. This suggests that accounting for variation in the biological replicates allowed PC1 to capture a higher proportion of the variance in the data. Furthermore, the PCA plot shows that samples in our analysis cluster clearly by experimental condition (Supplementary Figure 16). RNA-SeqEZPZ also provides alignment rate, percent of duplicates, percent reads that are assigned to a feature, and other raw reads statistics which were not included in RaNA-seq (Supplementary File 12).

Differential gene analysis

RaNA-seq identified 375 significant genes while RNA-SeqEZPZ identified 336 significant genes, using an FDR threshold of 0.05. Both pipelines identified Nfe2l2 as the most down-regulated gene along with a set of immune response genes (Cxcl1, Csf1, Ccl9, Cxcl12) that are known to promote tumorigenesis, as being upregulated in Nrf2 KO mice consistent with the previous finding [71] (Supplementary Figure 17). RNA-SeqEZPZ provides an interface to change the fold-change, FDR and mean normalized count difference cut-offs while RaNA-seq only provides FDR cut-off change.

Pathway analysis

Both RNA-SeqEZPZ and RaNA-seq shows enrichment of immune response. RNA-SeqEZPZ specifically indicates an up-regulation of cytokine, chemokine activity. Most importantly, RNA-SeqEZPZ's curated gene sets analysis revealed that genes down-regulated in Nrf2 KO compared to WT mice are significantly enriched in the NRF2 pathway [72], highlighting the potential relevance of these findings to human biology (Supplementary Figure 18).

Comparative analysis

In RaNA-seq, two analyses can be compared after they are individually analyzed. In contrast, in RNA-SeqEZPZ, all samples need to run together. This way, all samples will be normalized together to correct for library size and dispersion will incorporate the within-group variability across all groups which will make their expression values comparable, minimize batch effects such as GC-content, length or other technical biases. To enable comparative analysis, we re-analyzed the Nrf2 KO dataset using an FDR threshold of 0.1 (Nrf2_KO2) and compared the results to the previous Nrf2 KO vs WT analysis with FDR threshold of 0.05. A Venn diagram illustrating the overlap of significant genes between the two analyses is provided as the sole comparison output by RaNA-seq (Supplementary Figure 19A). In RNA-SeqEZPZ, we added a second Nrf2 KO samples and performed a similar comparative analysis, evaluating the

overlap of significant genes identified at FDR ≤ 0.05 and ≤ 0.1. Area-proportional Euler diagrams illustrate the overlap of significant genes, stratified by direction of regulation. Additionally, a gene overlap analysis along with Jaccard similarity index were conducted to quantify the degree of similarity (Supplementary Figure 19B). The analysis also includes pathway enrichment results for both comparison groups (Supplementary Figure 19C).

Some of the figures for the analyses of public datasets were generated using RNA-SeqEZPZ and modified using graphic editing software. ChatGPT [73] was utilized to assist in checking grammar and improving the clarity of the manuscript draft.

Discussions

In summary, RNA-SeqEZPZ provides an easy point-and-click comprehensive analysis of RNA-seq data which enables biologists to analyze and explore the nuances of their own experiments. The implementation of RNA-SeqEZPZ ensures reproducible analysis and is broadly flexible for running in various computational infrastructures. RNA-SeqEZPZ also provides an entry point analysis for more advanced users where they can download the results and do additional downstream analysis or modify the pipeline to include more features. Thus, RNA-SeqEZPZ represents a valuable easy-to-use tool for the scientific community, enabling the analysis, interpretation, and discovery of insights about gene function and regulation through RNA-seq experiments. By integrating Singularity container with workflow management systems and offering an end-to-end user interface, the codebase provides a flexible and extensible framework. It can be easily expanded to support additional interactive visualizations and more advanced analyses such as single cell RNA-seq, spatial transcriptomics and multiomics integration. For example, to enhance the precision and specificity of the differential gene detection in future iterations, we may incorporate a consensus-based approach as implemented in bestDEG [47].

Key points

- RNA-SeqEZPZ is a user-friendly pipeline with point-and-click interface starting from raw FASTQ files for comprehensive RNA-seq analysis, enabling both novice and experienced users to perform complex analyses with ease.
- RNA-SeqEZPZ enables researchers to analyze and compare differential gene expression across varying experimental conditions, with intuitive visualization tools for exploring and interpreting results.
- RNA-SeqEZPZ provides a containerized image and uses bioinformatics systems managers, ensuring straightforward installation, seamless deployment across environments, and reproducibility of the analyses performed.
- RNA-SeqEZPZ is freely available and can be downloaded from https://github.com/cxtaslim/RNA-SeqEZPZ and https://github.com/yzhang18/RNA-SeqEZPZ-NF (Nextflow version).

Additional files

Supplementary Table

Supplementary Table S1**:** A comparison of existing RNA-seq pipelines with RNA-SeqEZPZ.

Supplementary Figures

Supplementary Figure 1: RNA-SeqEZPZ workflow showing output files generated. Some icons were sourced and/or adapted from [74], created by Regan Hayward under the MIT license.

Supplementary Figure 2: A screenshot of the run analysis interface where users will be able to click-and-select their FASTQ files, reference genome, and other inputs. There is an “i” icon which will provide more information when hovered over in the interface.

Supplementary Figure 3: A snapshot of the log file providing information on the current progress of the RNA-seq analysis.

Supplementary Figure 4: A screenshot of the quality control report in interactive HTML format that can be viewed by users by clicking the “QCs” tab.

Supplementary Figure 5: A screenshot of the interface in RNA-SeqEZPZ to view and interact with statistical report generated automatically. The left navigation bar makes it easy to move to different section of the HTML file.

Supplementary Figure 6: A screenshot of the table interface showing the sorted log2 Fold-Change, False Discovery Rate (FDR) and read counts difference between treatment and control samples. Users can type in gene names to find their expressions.

Supplementary Figure 7: A screenshot of the interface where users can highlight specific genes in the volcano plot and change their significance cut-offs.

Supplementary Figure 8: A screenshot of the interface to create UpSet plot.

Supplementary Figure 9: A screenshot of the interface to perform overlap analysis which include generation of area-proportional Euler diagram, Venn diagram up to seven groups overlaps and heatmap showing the p-values of overlap and the Jaccard similarity index.

Supplementary Figure 10: A screenshot of the interface to perform pathway analysis with gene sets from MsigDB database. Enrichments are calculated for molecular function, biological processes, cellular components gene ontologies (GO), curated and Hallmark gene sets (not shown).

Supplementary Figure 11: The clean-up interface to assist users with removing big files such as aligned and merged FASTQ files.

Supplementary Figure 12: The interface to view the report generated by Nextflow.

Supplementary Figure 13: Volcano plot of cells treated with HCI 2509 run at (A) AWRI and (B) OSC.

Supplementary Figure 14: Expression of PAX3::FOXO1 in runs at (A) AWRI and (B) OSC.

Supplementary Figure 15: PCA plot of cells treated with HCI 2509, DMSO control, iLuc control and EWSR1::FLI1 KD (iEF) cells. (A) PCA plot prior to batch adjustment, showing clear separation between conditions. (B) Batch adjustment leads to mixing of replicates across conditions, arguing against using batch correction.

Supplementary Figure 16: PCA plots of Nrf2 KO samples generated using (A) RaNA-seq and (B) RNA-SeqEZPZ. The RNA-SeqEZPZ plot shows a higher proportion of variance explained by PC1, indicating improved separation after batch correction.

Supplementary Figure 17: Volcano plots of Nrf2 KO samples generated using (A) RaNA-seq, with red dots (from left to right) highlighting Nfe2l2, Clcx1, Cxcl12, and Csf1, and (B) the corresponding volcano plot produced by RNA-SeqEZPZ.

Supplementary Figure 18: Pathway enrichment analysis of Nrf2 KO samples generated using (A) RaNA-seq and (B) RNA-SeqEZPZ. Both analyses show similar enrichment of cytokine activity. Additionally, RNA-SeqEZPZ reveals enrichment of the human NRF2 pathway among down-regulated genes.

Supplementary Figure 19: Comparative analysis results. (A) Overlap of significant genes identified using RaNA-seq, (B) Overlap analysis of significant genes from RNA-SeqEZPZ, and (C) GO molecular function enrichment using RNA-SeqEZPZ, highlighting similar functional categories between the two significant gene sets.

Supplementary Figure 20: PCA plots illustrating the impact of batch correction on a larger dataset: (A) before adjustment, (B) after adjustment, with improved separation between conditions.

Supplementary Files

Supplementary File 1: QC report for the knockdown /rescue of EWSR1::FLI1 (iEF_EF) and EWSR1::ETV4 (iEF_EE4) in the A673 cell line. Link to Supplementary Files

Supplementary File 2: Statistical report for the knockdown/rescue of EWSR1::FLI1 (iEF_EF) and EWSR1::ETV4 (iEF_EE4) in the A673 cell line. PCA plot is shown in Fig.2A. Link to Supplementary Files

Supplementary File 3: Plots generated from the interface after the analysis of EWSR1::FLI1 and EWSR1::ETV4 knockdown/rescue in the A673 cell line. Selected plots are shown in Fig.2B-D. Link to Supplementary Files

Supplementary File 4: A list of up-regulated genes from the comparison of EWSR1::FLI1 knockdown/rescue to the empty construct. Link to Supplementary Files

Supplementary File 5: Video tutorial on how to run RNA-SeqEZPZ. Link to Supplementary Files

Supplementary File 6: Report generated by RNA-SeqEZPZ-NF for analysis of EWSR1::FLI1 and EWSR1::ETV4 knockdown/rescue in the A673 cell line, containing executed commands, CPU and memory usage, providing valuable insights for efficient resource management. Link to Supplementary Files

Supplementary File 7: Statistical report of the same analysis as presented in Supplementary File 2, but ran on OSC. Link to Supplementary Files

Supplementary File 8: Nextflow report for the analysis of EWSR1::FLI1 knockdown, iLuc control, HCI 2509 and DMSO control ran on AWRI. Link to Supplementary Files

Supplementary File 9: Nextflow report for the analysis of EWSR1::FLI1 knockdown, iLuc control, HCI 2509 and DMSO control ran on OSC. Link to Supplementary Files

Supplementary File 10: QC report for the analysis of Nrf2 KO and WT mice ran using RaNA-seq. Link to Supplementary Files

Supplementary File 11: QC report for the analysis of Nrf2 KO and WT mice ran using RNA-SeqEZPZ. Link to Supplementary Files

Supplementary File 12: Raw reads QC report for Nrf2 KO and WT mice samples generated by RNA-SeqEZPZ. Link to Supplementary Files

Availability of Source Code and Requirements

Project name: RNA-SeqEZPZ

Project homepage: https://github.com/cxtaslim/RNA-SeqEZPZ

Nextflow pipeline repository: https://github.com/yzhang18/RNA-SeqEZPZ-NF

License: CC BY-NC-ND 4.0 (RNA-SeqEZPZ), CC BY-NC-ND 4.0 (RNA-SeqEZPZ-NF)

Operating system: Platform independent

Programming language: R, Nextflow, Shell

Package management: Singularity container

Hardware requirements: depends on samples analyzed

WorkflowHub: https://doi.org/10.48546/WORKFLOWHUB.WORKFLOW.1813.2 (RNA-SeqEZPZ), https://doi.org/10.48546/WORKFLOWHUB.WORKFLOW.1814.2 (RNA-SeqEZPZ-NF)

Data Availability

New data for A673 cells treated with DMSO, or seclidemstat at IC50 and IC90 have been uploaded to NCBI Gene Expression Omnibus (GEO) repository (https://www.ncbi.nlm.nih.gov/geo/) under accession number GSE306637. Additional datasets used in this study are available in GEO as follows: GSE173185 (knockdown/rescue of EWSR1::FLI1 and EWSR1::ETV4), GSE98787 and GSE94503 (knockdown/rescue of EWSR1::FLI1, iLuc control, and cells treated with DMSO or HCI 2509), GSE268944 (TTC-466 samples), and

GSE270325 (zebrafish RNA-seq). The Supplemental Files 1, 2, 5-9, and 11-12, along with the archived software and input files are stored in the GigaDB [75].

Acknowledgments

This research was partially supported by the High Performance Computing Facility at the Abigail Wexner Research Institute (AWRI), Nationwide Children's Hospital. Assistance with figures provided by the AWRI Children's Graphics Resource Group and William Clarence Ray Ph.D. We acknowledge the contributions of the Kendall, Lessnick and Theisen lab members for their valuable assistance in testing and running the pipeline during its development.

Funding

E.R.T. is grateful for support from institutional startup funds, an American Cancer Society Research Scholar Grant and RSG-22-118-01-DMC, and an Unravel Pediatric Cancer grant. G.C.K. is grateful for support from an NIH/NCI R01 CA272872 grant, an Alex's Lemonade Stand Foundation "A" Award, a V Foundation for Cancer Research V Scholar Award, a CancerFree Kids New Idea Award, and Startup Funds from The Abigail Wexner Research Institute at Nationwide Children's Hospital. The funders had no role in study design, data collection and analysis, decision to publish, or preparation of the manuscript. Further, the content is solely the responsibility of the authors and does not necessarily represent the official views of the National Institutes of Health.

Competing Interest Statement

The authors declare no competing interests.

Author Contributions

C.T, Y.Z., G.C.K, E.R.T. conceived the main idea, the framework of the pipeline and the manuscript. C.T, Y.Z., G.C.K, E.R.T drafted and improved the manuscript. C.T and Y.Z. developed and implemented the pipeline. G.R performed the RNA-seq experiments, including cell culture, RNA extraction, and library preparation. G.C.K and E.R.T revised the manuscript, supervised the development of the pipeline, and provided funding. All authors read and commented on the manuscript.

References

1. Chen J-WW, Shrestha L, Green G, Leier AA, Marquez-Lago TT. The hitchhikers' guide to RNA sequencing and functional analysis. *Brief Bioinform*. Oxford University Press; 2023; doi: 10.1093/bib/bbac529.

2. Baykal PI, Łabaj PP, Markowetz F, Schriml LM, Stekhoven DJ, Mangul S, et al.. Genomic reproducibility in the bioinformatics era. *Genome Biol*. 2024; doi: 10.1186/s13059-024-03343-2.

3. Meyerowitz-Katz G, Besançon L, Flahault A, Wimmer R. Impact of mobility reduction on COVID-19 mortality: absence of evidence might be due to methodological issues. *Sci Rep*. Sci Rep; 2021; doi: 10.1038/S41598-021-02461-2.

4. Taslim C: RNA-SeqEZPZ: A Point-and-Click Pipeline for Comprehensive Transcriptomics Analysis with Interactive Visualizations. https://workflowhub.eu/workflows/1813 (2025). Accessed 2025 Jul 22.

5. Zhang Y, Taslim C: RNA-SeqEZPZ-NF: Nextflow Pipeline for RNA-SeqEZPZ. https://workflowhub.eu/workflows/1814 (2025). Accessed 2025 Jul 22.

6. Ewels PA, Peltzer A, Fillinger S, Patel H, Alneberg J, Wilm A, et al.. The nf-core framework for community-curated bioinformatics pipelines. *Nature Biotechnology 2020 38:3*. Nature Publishing Group; 2020; doi: 10.1038/S41587-020-0439-X.

7. Chang W, Cheng J, Allaire JJ, Sievert C, Schloerke B, Xie Y, et al.. shiny: Web Application Framework for R.

8. Farrel A, Li P, Veenbergen S, Patel K, Maris JM, Leonard WJ. ROGUE: an R Shiny app for RNA sequencing analysis and biomarker discovery. *BMC Bioinformatics*. BioMed Central Ltd; 2023; doi: 10.1186/S12859-023-05420-Y/FIGURES/4.

9. Sundararajan Z, Knoll R, Hombach P, Becker M, Schultze JL, Ulas T. Shiny-Seq: advanced guided transcriptome analysis. *BMC Res Notes*. NLM (Medline); 2019; doi: 10.1186/S13104-019-4471-1/FIGURES/1.

10. Moutsopoulos I, Williams EC, Mohorianu II. bulkAnalyseR: an accessible, interactive pipeline for analysing and sharing bulk multi-modal sequencing data. *Brief Bioinform*. Oxford Academic; 2023; doi: 10.1093/BIB/BBAC591.

11. Sundararajan Z: Shiny-Seq website. https://schultzelab.shinyapps.io/Shiny-Seq/ Accessed 2024 Oct 11.

12. Farrel A: ROGUE website. https://marisshiny.research.chop.edu/ROGUE/ Accessed 2024 Dec 9.

13. Prieto C, Barrios D. RaNA-Seq: interactive RNA-Seq analysis from FASTQ files to functional analysis. *Bioinformatics*. Oxford Academic; 2020; doi: 10.1093/BIOINFORMATICS/BTZ854.

14. Zhang X, Jonassen I. RASflow: An RNA-Seq analysis workflow with Snakemake. *BMC Bioinformatics*. BioMed Central Ltd.; 2020; doi: 10.1186/S12859-020-3433-X/TABLES/2.

15. Pedersen TL, Nijs V, Schaffner T, Nantz E. A Server-Side File System Viewer for Shiny [R package shinyFiles version 0.9.3]. *CRAN: Contributed Packages*. Comprehensive R Archive Network (CRAN); 2022; doi: 10.32614/CRAN.PACKAGE.SHINYFILES.

16. Yoo AB, Jette MA, Grondona M. SLURM: Simple Linux Utility for Resource Management. *Lecture Notes in Computer Science (including subseries Lecture Notes in Artificial Intelligence and Lecture Notes in Bioinformatics)*. Springer, Berlin, Heidelberg; 2003; doi: 10.1007/10968987_3.

17. DI Tommaso P, Chatzou M, Floden EW, Barja PP, Palumbo E, Notredame C. Nextflow enables reproducible computational workflows. *Nature Biotechnology 2017 35:4*. Nature Publishing Group; 2017; doi: 10.1038/nbt.3820.

18. : June 2024 | TOP500. https://top500.org/lists/top500/2024/06/ Accessed 2024 May 23.

19. Kurtzer GM, Sochat V, Bauer MW. Singularity: Scientific containers for mobility of compute. *PLoS One*. Public Library of Science; 2017; doi: 10.1371/JOURNAL.PONE.0177459.

20. Andrews. FastQC: a quality control tool for high throughput sequence data.

21. Ewels P, Magnusson M, Lundin S, Käller M. MultiQC: summarize analysis results for multiple tools and samples in a single report. *Bioinformatics*. Oxford University Press; 2016; doi: 10.1093/bioinformatics/btw354.

22. Lee D, Sangket U. VOE: automated analysis of variant epitopes of SARS-CoV-2 for the development of diagnostic tests or vaccines for COVID-19. *PeerJ*. PeerJ Inc.; 2024; doi: 10.7717/peerj.17504.

23. Krueger F: Trim Galore. https://www.bioinformatics.babraham.ac.uk/projects/trim_galore/ (2012).

24. Ewing B, Hillier LD, Wendl MC, Green P. Base-Calling of Automated Sequencer Traces UsingPhred. I. Accuracy Assessment. *Genome Res*. Cold Spring Harbor Laboratory Press; 1998; doi: 10.1101/GR.8.3.175.

25. Dobin A, Davis CA, Schlesinger F, Drenkow J, Zaleski C, Jha S, et al.. STAR: ultrafast universal RNA-seq aligner. *Bioinformatics*. 2013; doi: 10.1093/bioinformatics/bts635.

26. Liao Y, Smyth GK, Shi W. featureCounts: an efficient general purpose program for assigning sequence reads to genomic features. *Bioinformatics*. 2014; doi: 10.1093/bioinformatics/btt656.

27. Ramírez F, Ryan DP, Grüning B, Bhardwaj V, Kilpert F, Richter AS, et al.. deepTools2: a next generation web server for deep-sequencing data analysis. *Nucleic Acids Res*. Oxford Academic; 2016; doi: 10.1093/nar/gkw257.

28. Zerbino DR, Johnson N, Juettemann T, Wilder SP, Flicek P. WiggleTools: parallel processing of large collections of genome-wide datasets for visualization and statistical analysis. *Bioinformatics*. Oxford Academic; 2014; doi: 10.1093/BIOINFORMATICS/BTT737.

29. Love MI, Huber W, Anders S. Moderated estimation of fold change and dispersion for RNA-seq data with DESeq2. *Genome Biol*. 2014; doi: 10.1186/PREACCEPT-8897612761307401.

30. Varet H, Brillet-Guéguen L, Coppée J-Y, Dillies M-A. SARTools: A DESeq2- and EdgeR-Based R Pipeline for Comprehensive Differential Analysis of RNA-Seq Data. Mills K, editor. *PLoS One*. Public Library of Science; 2016; doi: 10.1371/journal.pone.0157022.

31. Benjamini Y, Hochberg Y. Controlling the False Discovery Rate: A Practical and Powerful Approach to Multiple Testing. *Journal of the Royal Statistical Society Series B (Methodological)*. Wiley for the Royal Statistical Society; 1995; doi: 10.2307/2346101.

32. Ritchie ME, Phipson B, Wu D, Hu Y, Law CW, Shi W, et al.. limma powers differential expression analyses for RNA-sequencing and microarray studies. *Nucleic Acids Res*. Oxford Academic; 2015; doi: 10.1093/NAR/GKV007.

33. Wickham H. ggplot2: Elegant Graphics for Data Analysis. Springer-Verlag New York;

34. Larsson J, Gustafsson P. A Case Study in Fitting Area-Proportional Euler Diagrams with Ellipses Using eulerr. *Proceedings of International Workshop on Set Visualization and Reasoning*. {Edinburgh, United Kingdom}: {CEUR Workshop Proceedings}; p. 84–91.

35. Dusa A. venn: Draw Venn Diagrams.

36. Gehlenborg N. UpSetR: A More Scalable Alternative to Venn and Euler Diagrams for Visualizing Intersecting Sets.

37. Fisher RA. On the Interpretation of χ 2 from Contingency Tables, and the Calculation of P. *Journal of the Royal Statistical Society*. JSTOR; 1922; doi: 10.2307/2340521.

38. Chung NC, Miasojedow BZ, Startek M, Gambin A. Jaccard/Tanimoto similarity test and estimation methods for biological presence-absence data. *BMC Bioinformatics*. BMC; 2019; doi: 10.1186/S12859-019-3118-5.

39. Shen L, Sinai M. GeneOverlap: Test and visualize gene overlaps.

40. Yu G, Wang L-G, Han Y, He Q-Y. clusterProfiler: an R package for comparing biological themes among gene clusters. *OMICS*. 2012; doi: 10.1089/omi.2011.0118.

41. Dolgalev I. msigdbr: MSigDB Gene Sets for Multiple Organisms in a Tidy Data Format.

42. Chen J-WW, Shrestha L, Green G, Leier AA, Marquez-Lago TT. The hitchhikers' guide to RNA sequencing and functional analysis. *Brief Bioinform*. Oxford University Press; 2023; doi: 10.1093/bib/bbac529.

43. Baruzzo G, Hayer KE, Kim EJ, DI Camillo B, Fitzgerald GA, Grant GR. Simulation-based comprehensive benchmarking of RNA-seq aligners. *Nat Methods*. Nature Publishing Group; 2016; doi: 10.1038/NMETH.4106.

44. Perelo LW, Gabernet G, Straub D, Nahnsen S. How tool combinations in different pipeline versions affect the outcome in RNA-seq analysis. *NAR Genom Bioinform*. Oxford Academic; 2024; doi: 10.1093/NARGAB/LQAE020.

45. Sarantopoulou D, Brooks TG, Nayak S, Mrčela A, Lahens NF, Grant GR. Comparative evaluation of full-length isoform quantification from RNA-Seq. *BMC Bioinformatics*. BioMed Central Ltd; 2021; doi: 10.1186/S12859-021-04198-1/FIGURES/12.

46. Rapaport F, Khanin R, Liang Y, Pirun M, Krek A, Zumbo P, et al.. Comprehensive evaluation of differential gene expression analysis methods for RNA-seq data. *Genome Biol*. BioMed Central; 2013; doi: 10.1186/GB-2013-14-9-R95/TABLES/2.

47. Sangket U, Yodsawat P, Nuanpirom J, Sathapondecha P. bestDEG: a web-based application automatically combines various tools to precisely predict differentially expressed genes (DEGs) from RNA-Seq data. *PeerJ*. 2022; doi: 10.7717/peerj.14344.

48. Meyerowitz-Katz G, Besançon L, Flahault A, Wimmer R. Impact of mobility reduction on COVID-19 mortality: absence of evidence might be due to methodological issues. *Sci Rep*. Sci Rep; 2021; doi: 10.1038/S41598-021-02461-2.

49. Botvinik-Nezer R, Holzmeister F, Camerer CF, Dreber A, Huber J, Johannesson M, et al.. Variability in the analysis of a single neuroimaging dataset by many teams. *Nature*. Nature Research; 2020; doi: 10.1038/S41586-020-2314-9.

50. Baykal PI, Łabaj PP, Markowetz F, Schriml LM, Stekhoven DJ, Mangul S, et al.. Genomic reproducibility in the bioinformatics era. *Genome Biol*. 2024; doi: 10.1186/s13059-024-03343-2.

51. Moreau D, Wiebels K, Boettiger C. Containers for computational reproducibility. *Nature Reviews Methods Primers*. 2023; doi: 10.1038/s43586-023-00236-9.

52. Wratten L, Wilm A, Göke J. Reproducible, scalable, and shareable analysis pipelines with bioinformatics workflow managers. *Nature Methods 2021 18:10*. Nature Publishing Group; 2021; doi: 10.1038/S41592-021-01254-9.

53. Kim YM, Poline JB, Dumas G. Experimenting with reproducibility: a case study of robustness in bioinformatics. *Gigascience*. Oxford Academic; 2018; doi: 10.1093/GIGASCIENCE/GIY077.

54. MerkelDirk. Docker. *Linux Journal*. Belltown MediaPUB6702Houston, TX; 2014; doi: 10.5555/2600239.2600241.

55. Chang W, Cheng J, Allaire JJ, Sievert C, Schloerke B, Xie Y, et al.. shiny: Web Application Framework for R.

56. Love MI, Huber W, Anders S. Moderated estimation of fold change and dispersion for RNA-seq data with DESeq2. *Genome Biol*. 2014; doi: 10.1186/PREACCEPT-8897612761307401.

57. Wilkinson L. Exact and approximate area-proportional circular venn and euler diagrams. *IEEE Trans Vis Comput Graph*. 2012; doi: 10.1109/TVCG.2011.56.

58. Conway JR, Lex A, Gehlenborg N. A More Scalable Alternative to Venn and Euler Diagrams for Visualizing Intersecting Sets [R package UpSetR version 1.4.0]. *Bioinformatics*. Comprehensive R Archive Network (CRAN); 2019; doi: 10.1093/BIOINFORMATICS/BTX364.

59. Wickham H. ggplot2: Elegant graphics for data analysis (use R!). New York: Springer;

60. Boone MA, Taslim C, Crow JC, Selich-Anderson J, Watson M, Heppner P, et al.. Identification of a Novel FUS/ETV4 Fusion and Comparative Analysis with Other Ewing Sarcoma Fusion Proteins. *Mol Cancer Res*. Mol Cancer Res; 2021; doi: 10.1158/1541-7786.MCR-21-0354.

61. Dreher RD, Theisen ER. Lysine specific demethylase 1 is a molecular driver and therapeutic target in sarcoma. *Front Oncol*. Frontiers Media S.A.; 2023; doi: 10.3389/FONC.2022.1076581/BIBTEX.

62. Luo W, Gangwal K, Sankar S, Boucher KM, Thomas D, Lessnick SL. GSTM4 is a microsatellite-containing EWS/FLI target involved in Ewing's sarcoma oncogenesis and therapeutic resistance. *Oncogene*. Oncogene; 2009; doi: 10.1038/ONC.2009.262.

63. Kinsey M, Smith R, Lessnick SL. NR0B1 is required for the oncogenic phenotype mediated by EWS/FLI in Ewing's sarcoma. *Mol Cancer Res*. Mol Cancer Res; 2006; doi: 10.1158/1541-7786.MCR-06-0090.

64. Center OS. Ohio Supercomputer Center.

65. Theisen ER, Pishas KI, Saund RS, Lessnick SL. Therapeutic opportunities in Ewing sarcoma: EWS-FLI inhibition via LSD1 targeting. *Oncotarget*. 2016; doi: 10.18632/oncotarget.7124.

66. Johnson KM, Mahler NR, Saund RS, Theisen ER, Taslim C, Callender NW, et al.. Role for the EWS domain of EWS/FLI in binding GGAA-microsatellites required for Ewing sarcoma anchorage independent growth. *Proc Natl Acad Sci U S A*. 2017; doi: 10.1073/pnas.1701872114.

67. Kucinski J, Tallan A, Taslim C, Vontell AM, Silvius KM, Wang M, et al.. Rhabdomyosarcoma fusion oncoprotein initially pioneers a neural signature in vivo. *Cell Rep*. Cell Press; 2025; doi: 10.1016/J.CELREP.2025.115923.

68. Bayanjargal A, Taslim C, Showpnil IA, Selich-Anderson J, Crow JC, Lessnick SL, et al.. The DBD-α4 helix of EWSR1::FLI1 is required for GGAA microsatellite binding that underlies genome regulation in Ewing sarcoma. *Elife*. eLife Sciences Publications Limited; 2024; doi: 10.7554/ELIFE.95626.2.

69. Rask GC, Taslim C, Bayanjargal A, Dreher RD, Cannon M V., Selich-Anderson J, et al.. Seclidemstat (SP-2577) induces transcriptomic reprogramming and cytotoxicity in multiple fusion-positive sarcomas. *Cancer Research Communications*. 2025; doi: 10.1158/2767-9764.CRC-24-0296.

70. Zhang Y, Parmigiani G, Johnson WE. ComBat-seq: batch effect adjustment for RNA-seq count data. *NAR Genom Bioinform*. Oxford Academic; 2020; doi: 10.1093/NARGAB/LQAA078.

71. Zhang D, Rennhack J, Andrechek ER, Rockwell CE, Liby KT. Identification of an unfavorable immune signature in advanced lung tumors from Nrf2-deficient mice. *Antioxid Redox Signal*. Mary Ann Liebert Inc.; 2018; doi: 10.1089/ARS.2017.7201,.

72. Fijten R SBEFWERZS-KMCEPADLCSHK: NRF2 pathway (WP2884) . https://www.wikipathways.org/instance/WP2884 Accessed 2025 Jul 6.

73. OpenAI. ChatGPT (GPT-4,Feb 12 version)[Large language model]. OpenAI;

74. Hayward R: nf-co.re worflow image link. https://raw.githubusercontent.com/nf-core/dualrnaseq/1.0.0//docs/images/Workflow_diagram_dualrnaseq.png Accessed 2024 Sep 11.

75.

Taslim C; Zhang Y; Rask G; Kendall GC; Theisen ER (): Supplementary files for "RNA-SeqEZPZ: A Point-and-Click Pipeline for Comprehensive Transcriptomics Analysis with Interactive Visualizations" GigaScience Database. https://doi.org/10.5524/102762

Figure 1
Click here to access/download;Figure;Fig_1_2.4.2.pdf

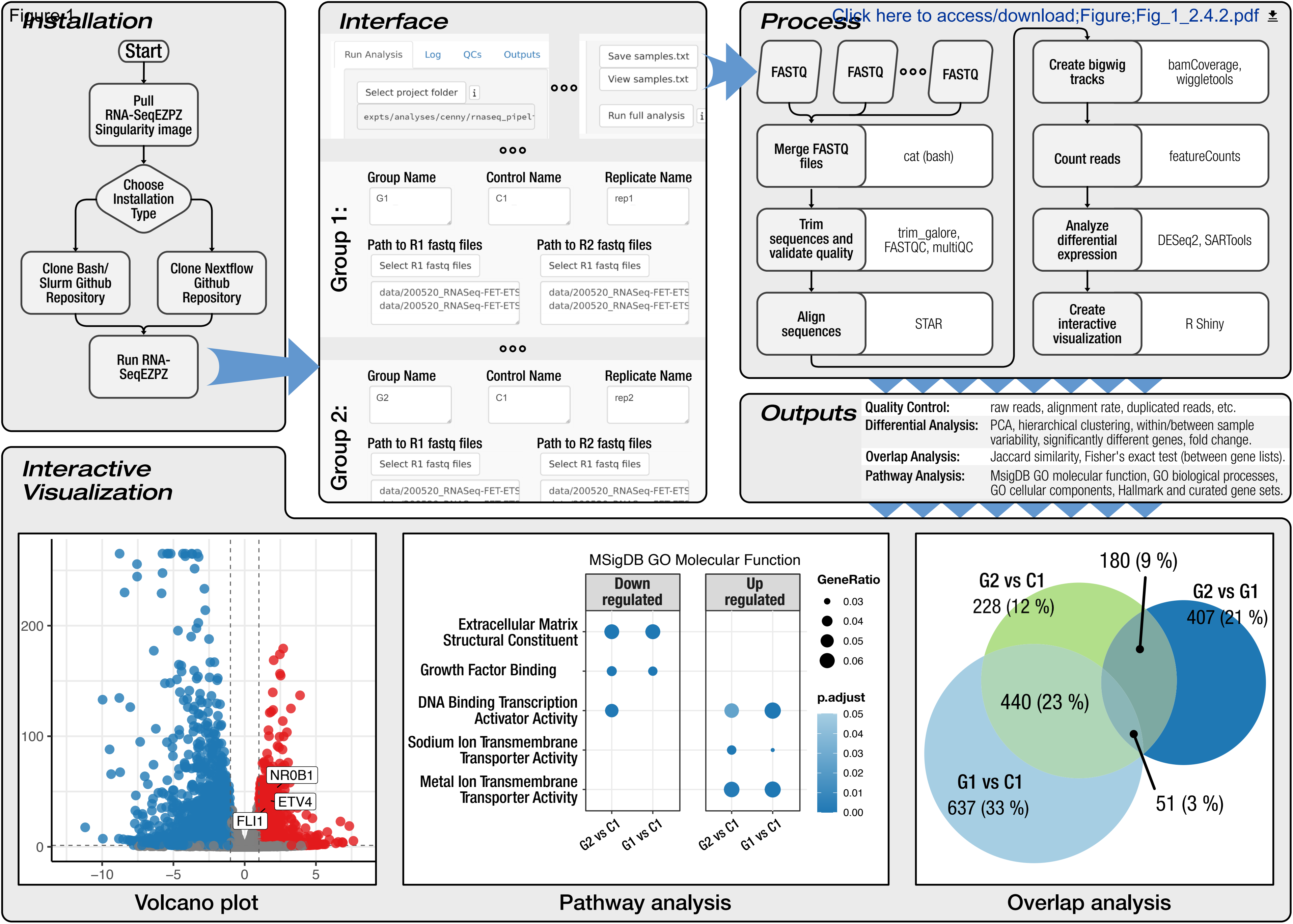

Figure 2

Click here to a[illegible]1.2.0.pdf

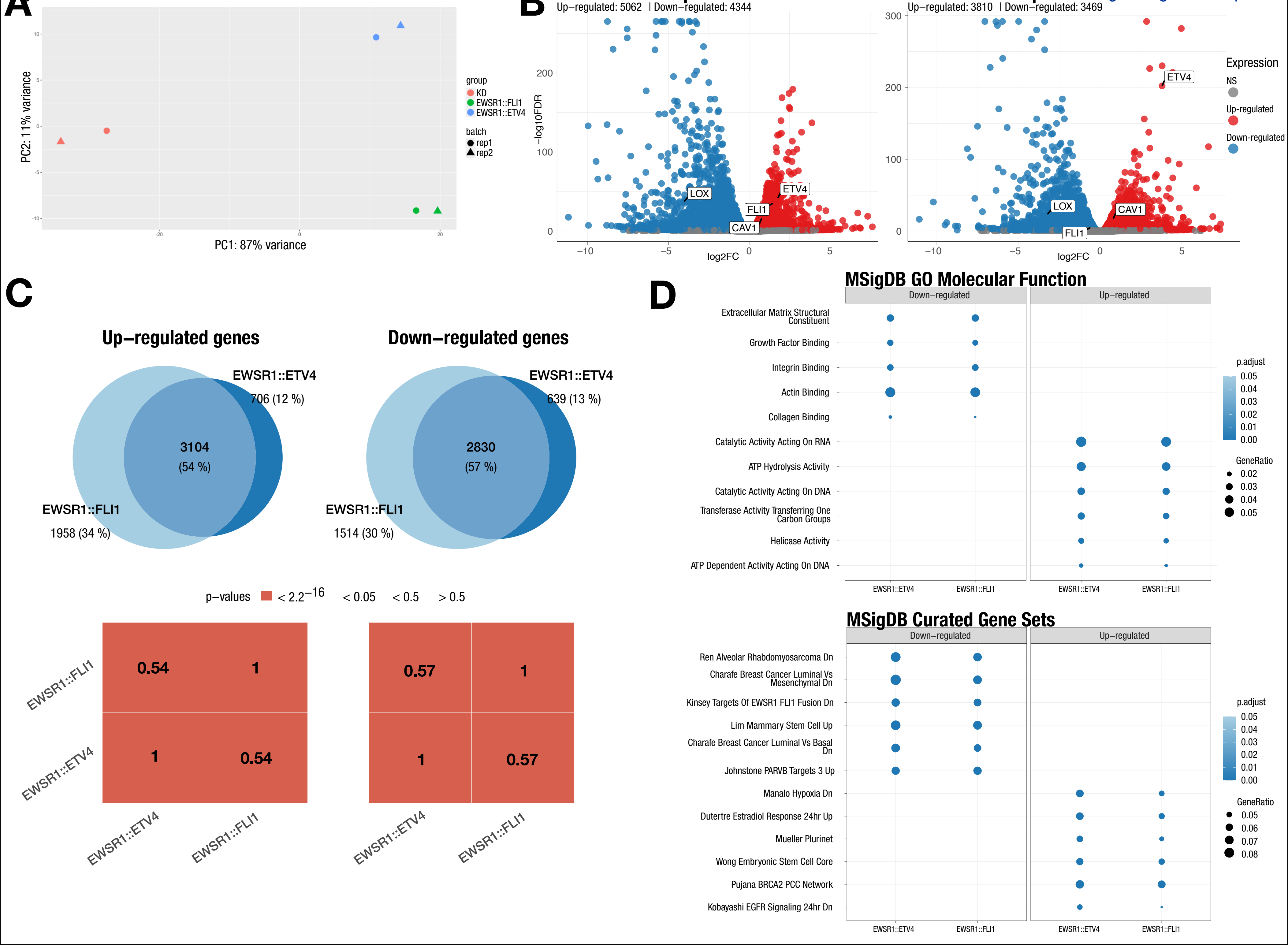

Figure 3

Click here to access/download;Figure;Fig_3_1.2.1.pdf

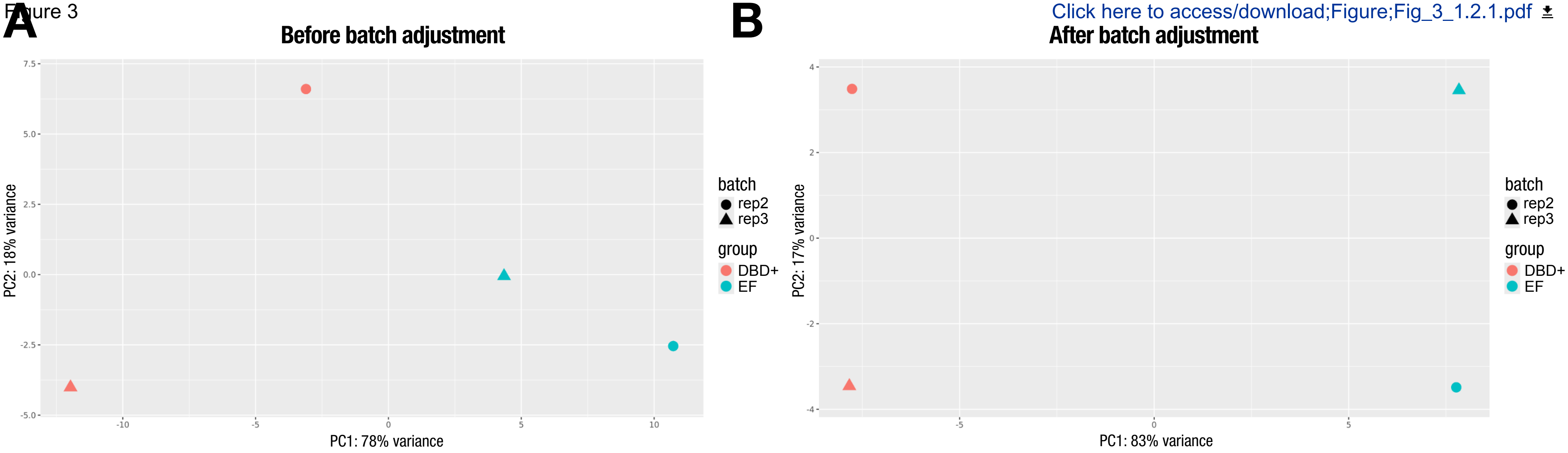

Supplementary Figure 20

Click here to access/download
Supplementary Material
Supplementary_Figure_20_PCA_seclidemstat.pdf

Supplementary Figure 1

Click here to access/download
Supplementary Material
Supplementary Figure 1.png

Supplementary Figure 2

Click here to access/download
Supplementary Material
Supplementary Figure 2.png

Supplementary Figure 3

Click here to access/download

Supplementary Material

Supplementary Figure 3.png

Supplementary Figure 4

Click here to access/download
Supplementary Material
Supplementary Figure 4.png

Supplementary Figure 5

Click here to access/download
Supplementary Material
Supplementary Figure 5.png

Supplementary Figure 6

Click here to access/download
Supplementary Material
Supplementary Figure 6.png

Supplementary Figure 7

Click here to access/download
Supplementary Material
Supplementary Figure 7.png

Supplementary Figure 8

Click here to access/download
Supplementary Material
Supplementary Figure 8.png

Supplementary Figure 9

Click here to access/download
Supplementary Material
Supplementary Figure 9.png

Supplementary Figure 10

Click here to access/download
Supplementary Material
Supplementary Figure 10.png

Supplementary Figure 11

Click here to access/download
Supplementary Material
Supplementary Figure 11.png

Supplementary Figure 12

Click here to access/download
Supplementary Material
Supplementary Figure 12.png

Supplementary Table S1

Click here to access/download
Supplementary Material
Supplementary_Table_S1_comparison_other_pipelines.docx

Click here to access/download
Supplementary Material
Supplementary_Figure_13_volcano_HCI2509.pdf

Click here to access/download
Supplementary Material
Supplementary_Figure_14_PAX3FOXO1_expr.pdf

Click here to access/download
Supplementary Material
Supplementary_Figure_15_PCA_HCI2509.pdf

Click here to access/download
Supplementary Material
Supplementary_Figure_16_PCA_Nrf2.pdf

Click here to access/download
Supplementary Material
Supplementary_Figure_17_volcano_Nrf2.pdf

Click here to access/download
Supplementary Material
Supplementary_Figure_18_pathway_Nrf2.pdf

Click here to access/download
Supplementary Material
Supplementary_Figure_19_comparative_RaNAseq_RNASeqEZPZ.pdf

Click here to access/download
Supplementary Material
Supplementary_File_3_plots_EE4.pdf

Click here to access/download
Supplementary Material
Supplementary_File_4_up_genes_EF.txt

Click here to access/download
Supplementary Material
Supplementary_File_10_Nrf2_RaNAseq_QC.pdf

cover letter
Click here to access/download;Personal Cover;CoverLetter_GigaScience.docx

August 25, 2025

GigaScience Editorial Team
Rm2102 21F Strand 50
50Bonhom Strand
Sheung Wan, Hong Kong

Dear Hongling Zhou and *GigaScience* editorial team,

We are pleased to submit our revised manuscript titled "**RNA-SeqEZPZ: A Point-and-Click Pipeline for Comprehensive Transcriptomics Analysis with Interactive Visualizations**" for consideration as a Technical Note in *GigaScience*. We added Galen Rask as a co-author because he performed the experiments that contributed data used in the validation of the pipeline for this round of revisions.

In this revised manuscript, we present a comprehensive pipeline that combines an end-to-end graphical user interface with the Nextflow workflow management system and Singularity containerization, aimed at democratizing RNA-seq data analysis for bench scientists.

We appreciate the reviewer's constructive feedback. Accordingly, we incorporated an analysis of a larger dataset generate by Mr. Rask to illustrate the improvement after correcting for batch-effects.

A point-by-point response to the reviewers' comments is included as a separate document.

We believe these revisions have strengthened the manuscript and hope it is now suitable for publication in *GigaScience*. Thank you for your continued consideration.

Sincerely,

Emily Theisen, PhD
Principal Investigator, Nationwide Children's Hospital
Assistant Professor, The Ohio State University College of Medicine

Genevieve Kendall, PhD
Principal Investigator, Nationwide Children's Hospital
Assistant Professor, The Ohio State University College of Medicine

Response to Reviewer

Reviewer 2 comment:

In Figure 3, only two replicates per condition are used to demonstrate batch adjustment. The claim that "after adjusting for batch effect, the DBD+ replicates cluster together, separating from EWSR1::FLI1 samples" is not convincing. A larger or more appropriate dataset would better support this conclusion.

We thank the reviewer for the valuable suggestion. In response, we have added an analysis of a larger dataset to better demonstrate the effect of batch adjustment. This dataset comprises three conditions, each with three biological replicates. The following sentences have been added to lines 343-350 in reference to a new Supplementary Figure 20:

To further demonstrate the benefit of batch correction on a larger dataset, we reanalyzed RNA-seq data from A673 Ewing sarcoma cells reported in "Seclidemstat blocks the transcriptional function of multiple FET-fusion oncoproteins"[67]. Cells were treated with vehicle (DMSO) or seclidemstat at IC50 or IC90, and RNA-seq was performed in biological triplicate. These samples are available in the GEO (GSE306637). Seclidemstat is currently in clinical trials for FET-rearranged sarcomas (NCT03600649). Prior to batch correction, IC50 and IC90 samples were intermixed. Following batch correction, distinct clusters emerge yielding clear dose specific clusters and increasing the variance explained by PC1 by 21% (Supplementary Figure 20).

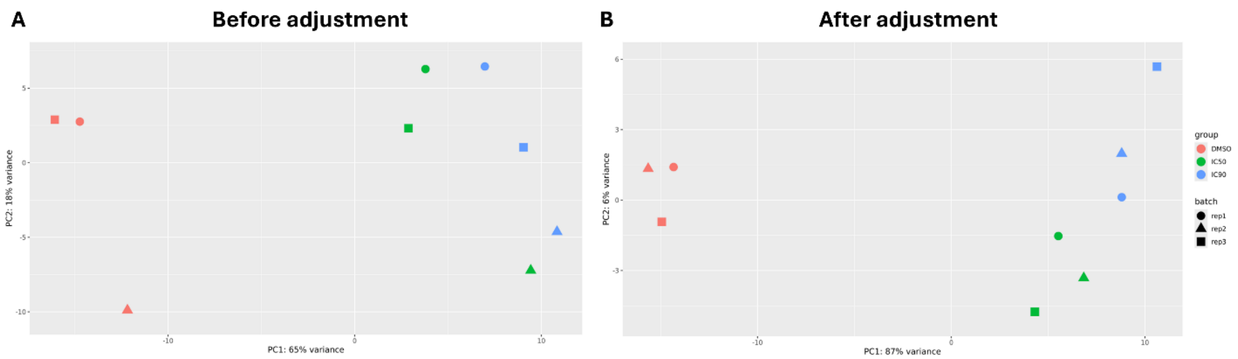

Supplementary Figure 20: PCA plots illustrating the impact of batch correction on a larger dataset: (A) before adjustment, (B) after adjustment, with improved separation between conditions.
