# Supplementary material for: RNA-SeqEZPZ: a point-and-click pipeline for comprehensive transcriptomics analysis with interactive visualizations
Source: Gigascience. 2025 Nov 12;15:giaf133. doi: 10.1093/gigascience/giaf133 (PMC12857227; doi:10.1093/gigascience/giaf133)
Supplement: giaf133_Reviewer_1_Report_Original_Submission — Unitsa Sangket, Ph.D. -- 5/2/2025 [file giaf133_reviewer_1_report_original_submission.pdf]

Reviewer Report

Title: RNA-SeqEZPZ: A Point-and-Click Pipeline for Comprehensive Transcriptomics Analysis with Interactive Visualizations

Version: Original Submission **Date:** 5/2/2025

Reviewer name: Unitsa Sangket, Ph.D.

Reviewer Comments to Author:

This research presents a well-designed and powerful program for comprehensive transcriptomics analysis with interactive visualizations. The tool is conceptually strong and user-friendly, requiring only raw reads in FASTQ format to initiate the analysis, with no need for manual quality checks. However, a limitation is that the software must be installed manually, which typically requires access to a high-performance computing (HPC) system and support from a system administrator for installation and server maintenance. As such, non-technical users may find it difficult to install and operate the program independently.With appropriate revisions based on the comments below, the manuscript has the potential to be significantly improved.- Page 8, line 158-160 "DESeq2 was selected based on findings by Rapaport et al. (2013)40,which demonstrated its superior specificity and sensitivity as well as good control of false positiveerrors." The findings in the paper titled "bestDEG: a web-based application automatically combines various tools to precisely predict differentially expressed genes (DEGs) from RNA-Seq data" (https://peerj.com/articles/14344) show that DESeq2 achieves higher sensitivity than other tools when applied to newer human RNA-Seq datasets. This finding should be included in the manuscript. For example, DESeq2 was selected based on findings by Rapaport et al. (2013)â�´â�°, which demonstrated its superior specificity and sensitivity as well as good control of false positiveerrors. Additionally, recent findings from the bestDEG study (cite bestDEG) further support the higher sensitivity of DESeq2 than other tools when applied to newer human RNA-Seq datasets.- Page 6, line 124-125 "Raw reads quality control are then performed using 125 FASTQC18 and QC reports are compiled using MultiQC19." The quality of the trimmed reads can be assessed using FastQC, as demonstrated and summarized in the paper titled "VOE: automated analysis of variant epitopes of SARS-CoV-2 for the development of diagnostic tests or vaccines for COVID-19." (https://peerj.com/articles/17504/) (Page 4, in last paragraph ""(1) Per base sequence quality (median value of each base greater than 25), (2) per sequence quality (median quality greater than 27), (3) perbase N content (N base less than 5% at each read position) and (4) adapter content (adapter sequences at each position less than 5% of all reads)". This point should be mentioned in the manuscript, including the cutoff values for each FastQC metrics used in RNA-SeqEZPZ, as these thresholds may vary. For example, the quality of the trimmed FASTQ reads was assessed based on the four FastQC metrics, as summarized by Lee et al. (2024). The cutoffs for RNA-SeqEZPZ were set as follows: the median value of each base must be greater than [x], the median quality score must be above [y], the percentage of N bases at each read position must be less than [z]%, and the proportion of adapter sequences at each position must be below [xx]% of all reads.- The programs used for counts table creation and alignment process should be mentioned in the manuscript.- The default cutoffs for

FDR and logâ,, fold change, as well as instructions on how to modify these thresholds, should be clearly stated in the manuscript.

Level of Interest

Please indicate how interesting you found the manuscript: Choose an item.

Quality of Written English

Please indicate the quality of language in the manuscript: Choose an item.

Declaration of Competing Interests

Please complete a declaration of competing interests, considering the following questions:

- Have you in the past five years received reimbursements, fees, funding, or salary from an organisation that may in any way gain or lose financially from the publication of this manuscript, either now or in the future?
- Do you hold any stocks or shares in an organisation that may in any way gain or lose financially from the publication of this manuscript, either now or in the future?
- Do you hold or are you currently applying for any patents relating to the content of the manuscript?
- Have you received reimbursements, fees, funding, or salary from an organization that holds or has applied for patents relating to the content of the manuscript?
- Do you have any other financial competing interests?
- Do you have any non-financial competing interests in relation to this paper?

If you can answer no to all of the above, write 'I declare that I have no competing interests' below. If your reply is yes to any, please give details below.

I declare that I have no competing interests.

I agree to the open peer review policy of the journal. I understand that my name will be included on my report to the authors and, if the manuscript is accepted for publication, my named report including any attachments I upload will be posted on the website along with the authors' responses. I agree for my report to be made available under an Open Access Creative Commons CC-BY license (http://creativecommons.org/licenses/by/4.0/). I understand that any comments which I do not wish to be included in my named report can be included as confidential comments to the editors, which will not be published.

Choose an item.

To further support our reviewers, we have joined with Publons, where you can gain additional credit to further highlight your hard work (see: https://publons.com/journal/530/gigascience). On publication of this paper, your review will be automatically added to Publons, you can then choose whether or not to claim your Publons credit. I understand this statement.

Yes Choose an item.
