# Supplementary material for: RNA-SeqEZPZ: a point-and-click pipeline for comprehensive transcriptomics analysis with interactive visualizations
Source: Gigascience. 2025 Nov 12;15:giaf133. doi: 10.1093/gigascience/giaf133 (PMC12857227; doi:10.1093/gigascience/giaf133)
Supplement: giaf133_Reviewer_2_Report_Original_Submission — Yang Yang -- 5/6/2025 [file giaf133_reviewer_2_report_original_submission.pdf]

Reviewer Report

Title: RNA-SeqEZPZ: A Point-and-Click Pipeline for Comprehensive Transcriptomics Analysis with Interactive Visualizations

Version: Original Submission **Date:** 5/6/2025

Reviewer name: Yang Yang

Reviewer Comments to Author:

The manuscript describes RNA-SeqEZPZ, an automated RNA-Seq analysis pipeline with a user-friendly point-and-click interface. It aims to make comprehensive transcriptomics analyses more accessible to researchers who lack extensive bioinformatics skills by addressing common issues with standardization and usability that arise from using in-house scripts. The pipeline's main features are the use of a Singularity container to simplify software installation and a Nextflow version to support scalability across different computing environments like clouds and clusters. However, I'm not sure if this manuscript fits the journal's scope in its current form. It seems to be just an integration of existing tools without offering new methods or findings.Major comments:1. The manuscript mentions several existing RNA-Seq pipelines, such as ENCODE, nf-core, ROGUE, Shiny-Seq, bulkAnalyseR, Partekâ„¢ flow, RaNA-Seq, and RASflow. A more detailed comparison of RNA-SeqEZPZ with these tools is needed, especially regarding specific features, performance metrics, and ease of use. For example, it would be helpful to compare the computational resources required by each pipeline or the statistical methods used for differential expression analysis.2. The manuscript emphasizes reproducibility through Singularity containers and Nextflow. However, it would be stronger if it included a more rigorous demonstration of reproducibility. This could involve running the pipeline on multiple datasets and comparing the results, or providing a detailed protocol for other researchers to reproduce the findings.3. The manuscript highlights the scalability and portability of RNA-SeqEZPZ due to its Nextflow version. It would be useful to include specific examples of how the pipeline has been used in different computing environments (e.g., cloud, cluster) and to provide performance data to demonstrate its scalability.4. The point-and-click interface is a key feature, but the manuscript could benefit from a more detailed description of the interface and its functionalities. Including screenshots or a video demonstration would be valuable for potential users.5. The manuscript shows the effects of batch adjustment using a public dataset. It would be beneficial to expand this section with a discussion of the limitations of batch adjustment methods and to provide guidance on when and how to apply them.

Level of Interest

Please indicate how interesting you found the manuscript: Choose an item.

Quality of Written English

Please indicate the quality of language in the manuscript: Choose an item.

Declaration of Competing Interests

Please complete a declaration of competing interests, considering the following questions:

- Have you in the past five years received reimbursements, fees, funding, or salary from an organisation that may in any way gain or lose financially from the publication of this manuscript, either now or in the future?
- Do you hold any stocks or shares in an organisation that may in any way gain or lose financially from the publication of this manuscript, either now or in the future?
- Do you hold or are you currently applying for any patents relating to the content of the manuscript?
- Have you received reimbursements, fees, funding, or salary from an organization that holds or has applied for patents relating to the content of the manuscript?
- Do you have any other financial competing interests?
- Do you have any non-financial competing interests in relation to this paper?

If you can answer no to all of the above, write 'I declare that I have no competing interests' below. If your reply is yes to any, please give details below.

I declare that I have no competing interests

I agree to the open peer review policy of the journal. I understand that my name will be included on my report to the authors and, if the manuscript is accepted for publication, my named report including any attachments I upload will be posted on the website along with the authors' responses. I agree for my report to be made available under an Open Access Creative Commons CC-BY license (http://creativecommons.org/licenses/by/4.0/). I understand that any comments which I do not wish to be included in my named report can be included as confidential comments to the editors, which will not be published.

Choose an item.

To further support our reviewers, we have joined with Publons, where you can gain additional credit to further highlight your hard work (see: https://publons.com/journal/530/gigascience). On publication of this paper, your review will be automatically added to Publons, you can then choose whether or not to claim your Publons credit. I understand this statement.

Yes Choose an item.
